# Larger forest patches have greater per-area productivity

**DOI:** 10.1038/s41559-026-03075-5

**Published:** 2026-05-15

**Authors:** Yibiao Zou, Gabriel Reuben Smith, Thomas Lauber, Joe Wan, Haozhi Ma, Noel Gorelick, Constantin M. Zohner, Thomas W. Crowther

**Affiliations:** 1 Swiss Federal Institute for Forest, Snow and Landscape Research WSL, Birmensdorf, Switzerland; 2 BRANCH Institute, Zug, Switzerland; 3 Institute of Integrative Biology, ETH Zürich (Swiss Federal Institute of Technology), Zurich, Switzerland; 4 Institute of Ecology and Evolutionary Biology, National Taiwan University, Taipei, Taiwan; 5 Google Switzerland GmbH, Zurich, Switzerland; 6 Remote Sensing Laboratories, University of Zürich, Zurich, Switzerland; 7 Institute for Future Initiatives, University of Tokyo (UTokyo), Tokyo, Japan; 8 King Abdullah University of Science and Technology (KAUST), Thuwal, Saudi Arabia

**Keywords:** Ecosystem ecology, Forest ecology

## Abstract

Forest fragmentation could reduce carbon sequestration beyond losses caused by declining forest area alone, if smaller patches are intrinsically less productive per unit area than larger ones. Here we analyse 17 million forest patches across the conterminous USA and show that per-area net primary productivity increases systematically with patch size. A hectare embedded within a ~100,000 km^2^ forest is, on average, 38% more productive than an isolated hectare under comparable environmental conditions. Counterfactual analyses indicate that existing fragmentation has already reduced total annual forest productivity across the conterminous USA by 0.16 GtC yr^−1^ or 14% relative to an upper-bound configuration of large, contiguous forests. Random forest models identify patch size as a stronger predictor of net primary productivity than topographic and soil variables. Extending the analysis globally with coarser-resolution data, we find consistent positive relationships between patch size and per-area productivity for both net and gross primary productivity. These results demonstrate that forest fragmentation can reduce carbon sequestration without net forest-area loss, highlighting the need to account for fragmentation—not just forest cover—in climate mitigation strategies.

## Abstract (web summary)

Using remote sensing data, spatial modelling and counterfactual scenarios, this analysis reveals a positive relationship between forest patch size and productivity. This implies that preserving many small forest patches may not offset the carbon losses from larger contiguous forests.

## Main

Forests cover 31% of the Earth’s land surface^1^ and absorb roughly a quarter of anthropogenic carbon emissions each year^2–4^. Forests moderate local temperatures^5^, provide habitat to terrestrial biodiversity^6–8^ and support human livelihoods^9^. Globally, forests store approximately 861 Pg of carbon in soil, plant biomass, dead wood and litter^10^. However, these important ecosystem services are increasingly threatened by land use and global change^10,11^. Since 1990, 420 million ha of forest have been lost to deforestation^1^, with further impacts on the integrity of remaining forest.

Forest loss increasingly fragments forested landscapes into smaller, more isolated patches with disproportionately high edge exposure^12,13^. Compared with large, contiguous forests, these fragments often support fewer species due to habitat loss^14^ and store less biomass because edges experience harsher microclimates and elevated tree mortality^15–18^. Consequently, fragmentation can drive forest degradation, where forest cover persists but biomass stocks decline^19^. Decades of theoretical and empirical work, therefore, suggest that key ecosystem functions should differ systematically between large and small forest patches^12,15,16^. However, existing evidence has focused primarily on standing carbon stocks^20^ or structural attributes, typically at local or regional scales^21,22^. By contrast, the implications of fragmentation for forest productivity—carbon sequestration rates and fluxes (for example, net primary productivity (NPP) and gross primary productivity (GPP))—and their scaling with patch size across landscapes remain poorly quantified at broad spatial extents^23^.

This gap has direct policy relevance because efforts to protect forests and maintain their carbon sink capacity focus primarily on total forest area^1,24^. Such area-based approaches implicitly assume that a hectare in a large, contiguous forest patch is functionally equivalent to a hectare in a small, isolated patch. If, however, forest productivity depends on patch size, then current policy frameworks risk overlooking the carbon losses associated with forest fragmentation by treating several small patches as interchangeable with one large patch of the same total area. For example, ‘zero net deforestation’ commitments—widely adopted by companies, governments and non-governmental organizations—are typically defined by net change in forest area, allowing forest clearance or conversion in one location if compensated by regrowth or reforestation elsewhere^25,26^. This approach has been criticized for its conceptual and practical limitations, including ambiguous definitions, implementation challenges and failing to account for ecological integrity^25–29^. Under such frameworks, landscapes can become increasingly fragmented without violating headline targets if total forest area remains stable, even though configuration changes may still reduce ecosystem functions such as carbon uptake and storage. These dynamics underscore the need to empirically determine how forest productivity scales with patch size—and whether conserving forest extent alone is sufficient to preserve carbon sequestration capacity.

**Fig. 1:**
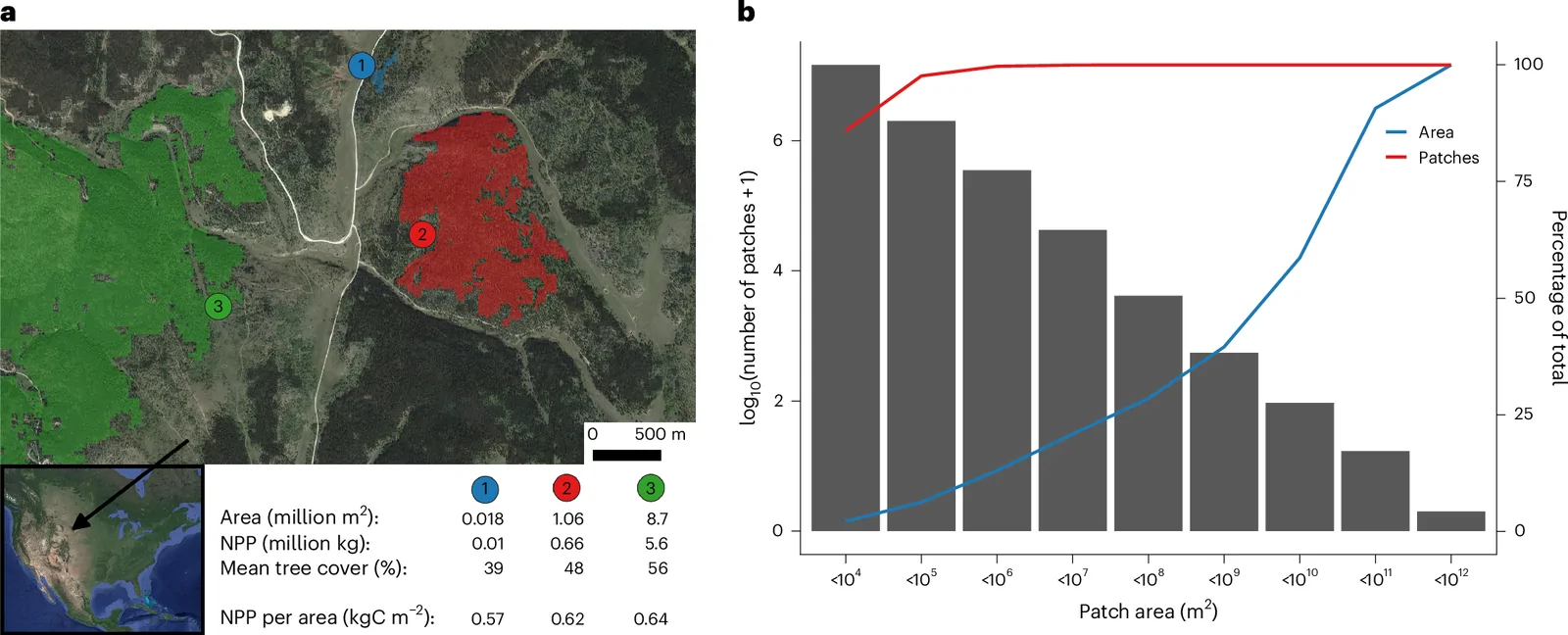
Forest patch delineation and size distribution across the CONUS. **a**, Three example patches illustrating our delineation and measurement of forest patch polygons across the CONUS based on high-resolution (30 m) forest^24^ and NPP^35^ data products. Non-focal patch polygons are rendered in grey. **b**, Distribution of forest patches of varying sizes across the CONUS (*n* = 16,959,582). Map data in **a** ©2026 Google, INEGI; imagery ©2026 NASA GS (2011) 6020. Source data

Here we quantify forest productivity and patch size across the conterminous USA (CONUS) by analysing 17 million forest patches (Fig. 1a and Supplementary Fig. 1). As observed in other regions^30^, patch sizes follow a heavy-tailed Pareto distribution, where the vast majority of patches are small (<10^4^ m^2^) and collectively contribute little to total forest area (Fig. 1b). We then estimate how forest productivity varies with patch size, explicitly testing the magnitude and significance of the patch-size effect while controlling for spatial and environmental heterogeneity. Finally, we extend the analysis globally using coarser-resolution productivity products to assess whether the observed scaling relationship generalizes beyond CONUS.

## Results and discussion

### Superlinear scaling of productivity with patch size

**Fig. 2:**
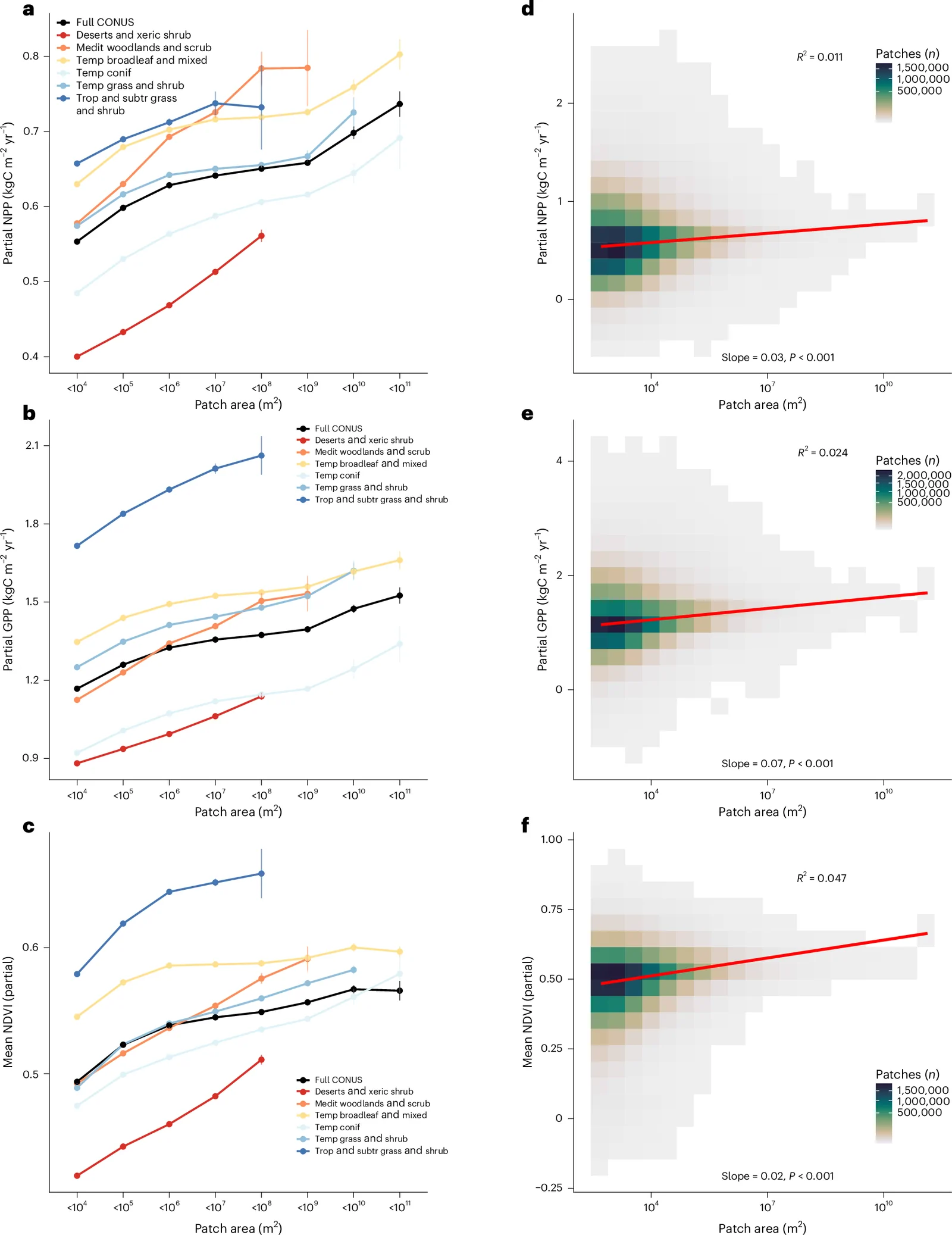
Biome-specific scaling of forest productivity with patch size. **a**–**c**, Biome-specific annual NPP per square metre (**a**), annual GPP per square metre (**b**) and mean NDVI (**c**) in forest patches (*n* = 16,913,643 for full CONUS), controlling for intrabiome variation in socio-environmental conditions and spatial autocorrelation (Methods). Data are presented as mean values ± standard error of the mean. We do not examine biomes with fewer than 100,000 forest patches, nor do we render points for patch-size classes with fewer than three observations. **d**–**f**, Annual NPP per square metre (**d**), annual GPP per square metre (**e**) and mean NDVI (**f**), controlling for socio-environmental conditions and spatial autocorrelation as in **a**–**c**, of forest patches in the full CONUS rendered as a function of patch size, with a fitted regression line for visualization purposes (*n* = 16,918,882). Values can be outside the bounds of the measurement because they are partial residuals. Slopes correspond to the estimated fixed-effect coefficient of log_10_(patch size) from linear mixed-effects models, and statistical significance was assessed using two-sided Wald tests of the regression coefficient. While model *R*^2^ reflects predictive performance, our primary goal is not to forecast productivity—which is already available from geospatial products—but to statistically test an a priori biological hypothesis: that per-area productivity scales with patch size. Thus, the regression slopes and corresponding *P* values in **d**–**f** are the key metrics for inference. conif, coniferous; grass, grassland; Medit, Mediterranean; shrub, shrubland; subtr, subtropical; temp, temperate; trop, tropical. Source data

Using a spatial random effect approach to control for large-scale socio-environmental variation and spatial autocorrelation (Methods), we discovered positive relationships between forest patch size and mean normalized difference vegetation index (NDVI), as well as predicted annual NPP and GPP per square metre (Fig. 2). Within biomes, residual spatial autocorrelation was minimal, with Moran’s *I* always below 0.09 (Supplementary Table 1).

If annual NPP per unit area (NPP per square metre) were independent of patch size, then total annual NPP would increase linearly with patch area. Under such linear scaling, a hectare of forest in a small patch would have the same NPP as a hectare in a large patch. Instead, we observe ‘superlinear’ scaling—under comparable socio-environmental conditions, larger patches exhibited higher NPP, GPP and NDVI per unit area (Fig. 2). As a result, forest productivity increases more than proportionally with patch size.

We used the fitted models to quantify the expected difference in annual NPP per square metre between socio-environmentally analogous patches in the smallest and largest patch-size classes (restricting comparisons to classes with ≥3 patches per biome; Table 1). Across CONUS, predicted productivity increased by 38% from the smallest to the largest patches. Biome-specific effects varied, with a comparatively small increase in tropical or subtropical grassland and shrubland (~14%) and the strongest increase in temperate coniferous forests (45%).

**Table 1:** Biome-specific comparisons of model-predicted annual NPP per square metre for two socio-environmentally equivalent forest patches varying only in size

Biome	Size comparison (m^2^)	Productivity (kgNPP m^−^^2^ yr^−1^)	Percent increase
Full CONUS	10^4^ versus 10^11^	0.58 versus 0.80	37.83%
Deserts and xeric shrubland	10^4^ versus 10^8^	0.42 versus 0.52	23.80%
Mediterranean woodlands and scrub	10^4^ versus 10^9^	0.61 versus 0.82	35.54%
Temperate broadleaf and mixed forests	10^4^ versus 10^11^	0.66 versus 0.88	34.13%
Temperate coniferous forests	10^4^ versus 10^11^	0.51 versus 0.74	44.57%
Temperate grassland and shrubland	10^4^ versus 10^10^	0.60 versus 0.78	30.02%
Tropical and subtropical grassland and shrubland	10^4^ versus 10^8^	0.68 versus 0.77	14.21%

The two sizes reflect the smallest and largest size classes observed within the biome, excluding size classes with fewer than three forest patches. In all cases the model term being adjusted to generate the predictions is statistically significant.

### Counterfactual comparison

To quantify the potential consequences of fragmentation on forest productivity at a continental scale, we conducted a counterfactual comparison that holds total forest area constant while varying the size–productivity relationship across three landscape configurations. Using the empirically estimated relationship between per-area NPP and patch size, we computed total annual NPP across CONUS under (1) a least-fragmented scenario, in which all forest area was assigned the per-area productivity of the largest patch-size class (Table 1), (2) the realized configuration, based on observed patch-specific productivity and (3) a most-fragmented scenario, in which all forest area was assigned the per-area productivity of the smallest patch-size class (Table 1).

**Fig. 3:**
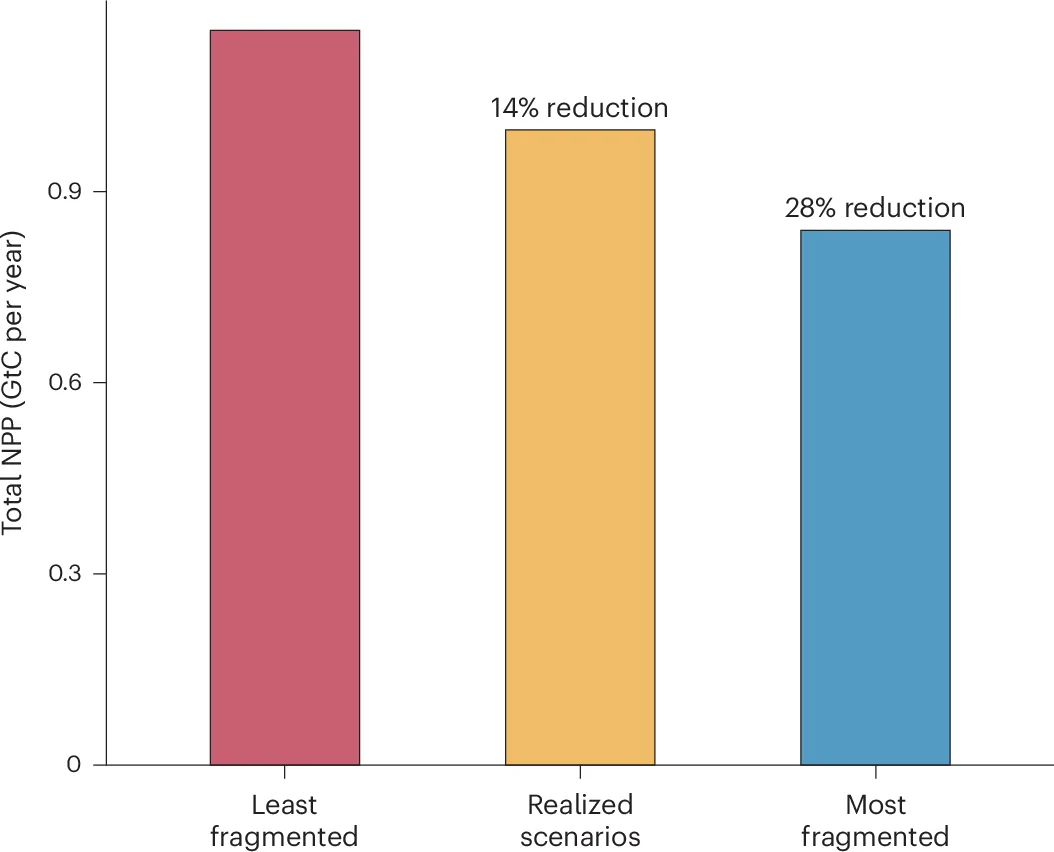
Counterfactual effects of forest fragmentation on total annual NPP across CONUS. Bars show total forest NPP (GtC yr^−1^) under three fragmentation scenarios while holding total forest area constant. ‘Least fragmented’ represents an upper-bound configuration in which all forest area is assumed to function as large, contiguous patches and is assigned a per-area productivity of 0.80 kgC m^−^^2^ yr^−1^, corresponding to the mean NPP of the largest patch-size class (~10^11^ per square metre; Table 1). ‘Realized’ represents the observed landscape configuration, where total NPP is computed by summing patch-specific area multiplied by each patch’s empirically estimated per-area NPP. ‘Most fragmented’ represents a lower-bound configuration in which all forest area is assumed to function as small, highly fragmented patches and is assigned a per-area productivity of 0.58 kgC m^−^^2^ yr^−1^, corresponding to the mean NPP of the smallest patch-size class (~10^4^ per square metre; Table 1). Percentage labels indicate reductions in total NPP relative to the least-fragmented scenario, highlighting the potential carbon sequestration losses attributable to fragmentation alone. Source data

Under the realized configuration, total forest NPP was approximately 14% (or 0.16 GtC yr^−1^) lower than in the least-fragmented scenario (Fig. 3), indicating that current fragmentation already reduces continental-scale carbon uptake relative to an upper-bound configuration dominated by large, contiguous forests. Under the most-fragmented scenario, total NPP declined by 28% (or 0.32 GtC yr^−1^) relative to the least-fragmented scenario, illustrating the magnitude of potential productivity losses attributable to fragmentation alone. Together, these results demonstrate that forest configuration substantially influences forest productivity, and that maintaining or restoring large, contiguous forest patches could increase carbon uptake without increasing total forest area.

### Testing for importance of patch-size effects

To test whether forest patch size explains variation in productivity beyond local environmental conditions, we trained random forest models on ~240,000 randomly sampled forest pixels across CONUS, using NPP as the response variable and a common set of climatic, topographic and soil covariates^31–34^. To isolate the independent contribution of patch size, we fit two otherwise identical models: one including log_10_-transformed patch size as an additional predictor and one excluding it.

**Fig. 4:**
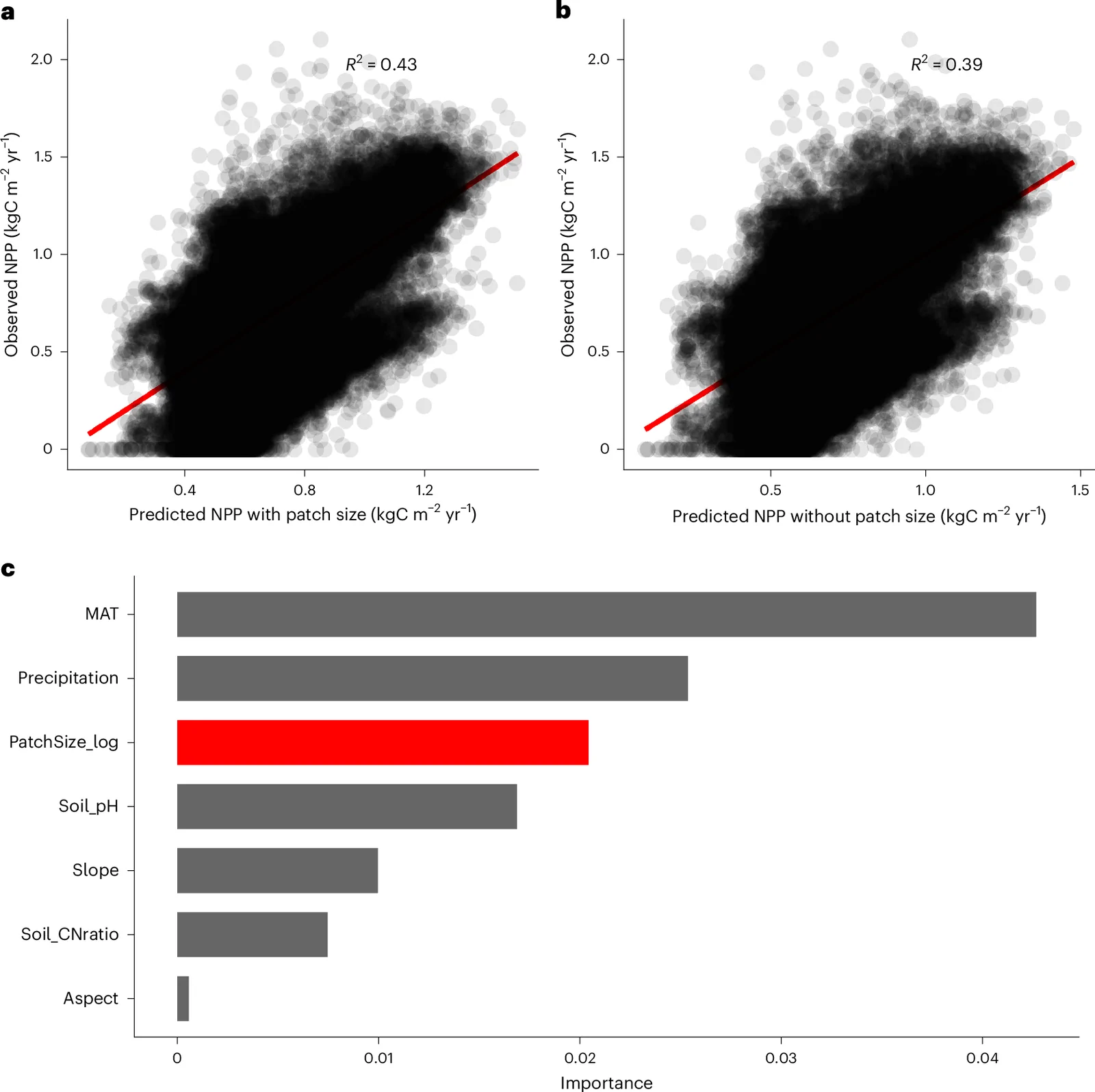
Contribution of forest patch size to predicting forest productivity. **a**, Observed versus predicted NPP from a random forest model that includes forest patch size (log-transformed) together with climatic, topographic and soil covariates. The red line shows the fitted linear relationship, and *R*^2^ indicates overall model explanatory power. **b**, As in **a**, but for an otherwise identical random forest model excluding patch size as a predictor. Model performance is reduced relative to **a**, indicating that patch size contributes independent explanatory power beyond environmental covariates. **c**, Permutation-based variable importance from the random forest model shown in **a**. Forest patch size (log_10_-transformed; highlighted in red) ranks among the most influential predictors of NPP, comparable in magnitude to key climatic and edaphic variables. Random forest models are trained on a dataset of ~240,000 randomly sampled forest pixels across the CONUS. MAT, mean annual temperature; PatchSize_log, log_10_-transformed forest patch size; Soil_CNratio, soil carbon-to-nitrogen ratio. Source data

Including patch size improved predicative performance (coefficient of determination, *R*^2^ = 0.43; Fig. 4a) compared with the model without patch size (*R*^2^ = 0.39; Fig. 4b), indicating that patch size adds explanatory power beyond environmental controls alone. Permutation-based variable importance further ranked patch size among the most influential predictors of NPP, comparable in magnitude to mean annual temperature and precipitation, and exceeding several edaphic and topographic variables (Fig. 4c). These results indicate that forest patch size captures a distinct component of productivity variation that is not fully explained by local environmental gradients.

### Addressing potential limitations

One potential limitation is that our NPP and GPP products draw on NDVI^35^, and we also find higher NDVI in larger patches (Fig. 2 and Supplementary Table 1). This shared dependence on optical reflectance could, in principle, accentuate the observed trends. To test whether the relationship is driven by Landsat-based reflectance, we repeated the analysis using vegetation optical depth (VOD)^36^, a passive-microwave proxy for canopy water content, productivity and biomass^37–39^ that is independent of optical reflectance. To match the 9-km VOD resolution, we restricted the CONUS sample to patches ≥81 km^2^. Despite this substantially coarser scale and the reduced sample, the patch-size signal persisted: larger patches exhibited higher mean VOD per area, and patch size explained a substantial fraction of variation in VOD (*R*^2 ^= 0.19). These results support the interpretation that the observed scaling of productivity with patch size is not simply a by-product of shared Landsat reflectance (Supplementary Fig. 2 and Supplementary Methods).

### Potential mechanisms

**Extended Data Fig. 1:**
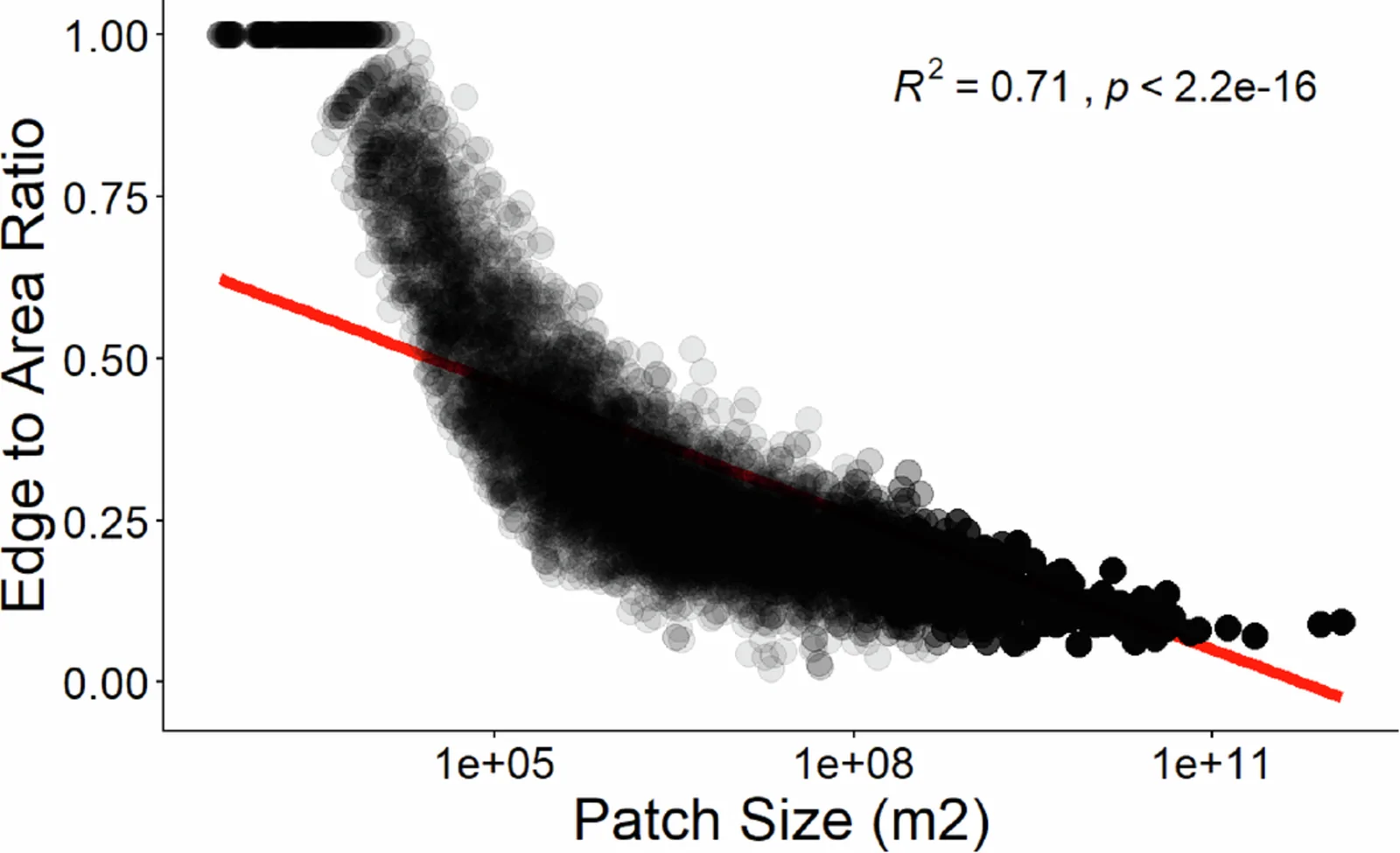
Relationship between forest patch size (m^2^, log scale) and the edge-to-area ratio, a geometric measure of edge exposure, across forest patches in the conterminous United States. Larger patches exhibit a sharply lower proportion of edge per unit area, reflecting the fundamental geometry of fragmentation. The fitted regression line (red) highlights a strong negative relationship (R^2^ = 0.71, p < 2.2 × 10^−16^), indicating that small patches are disproportionately dominated by edge environments. This scaling relationship provides a mechanistic link between fragmentation, elevated edge influence, and reduced per-area productivity in small forest patches. Source data

**Extended Data Fig. 2:**
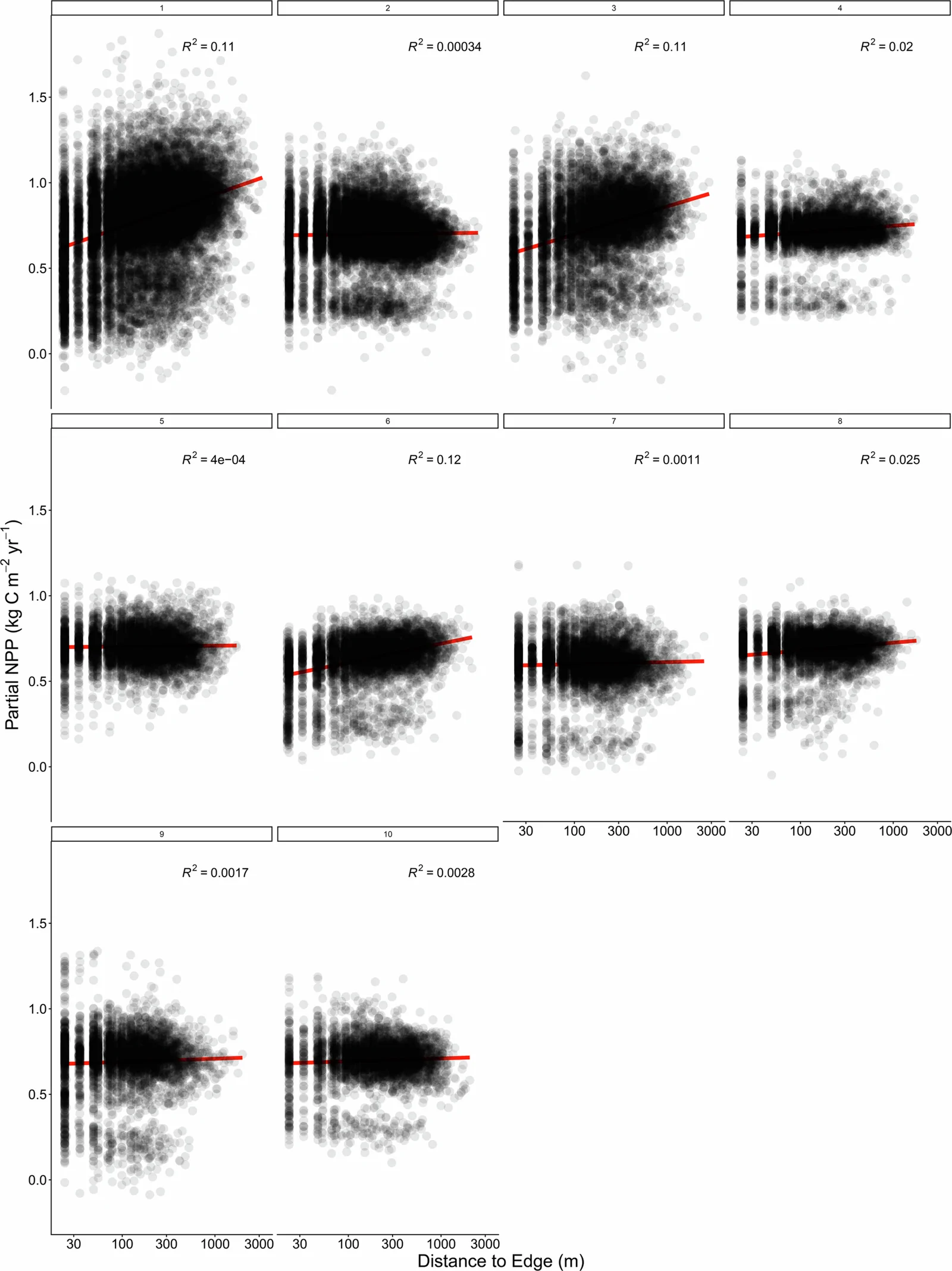
Scatterplots show the relationship between topography-detrended annual NPP (kg C m⁻^2^ yr^−1^) and distance to forest edge (m) for each of the 10 largest forest patches in the conterminous United States (panels 1–10). Each point represents a forest pixel; red lines indicate linear fits shown for visualization. NPP values were detrended with respect to topographic covariates to isolate edge-related effects within patches. Source data

**Extended Data Fig. 3:**
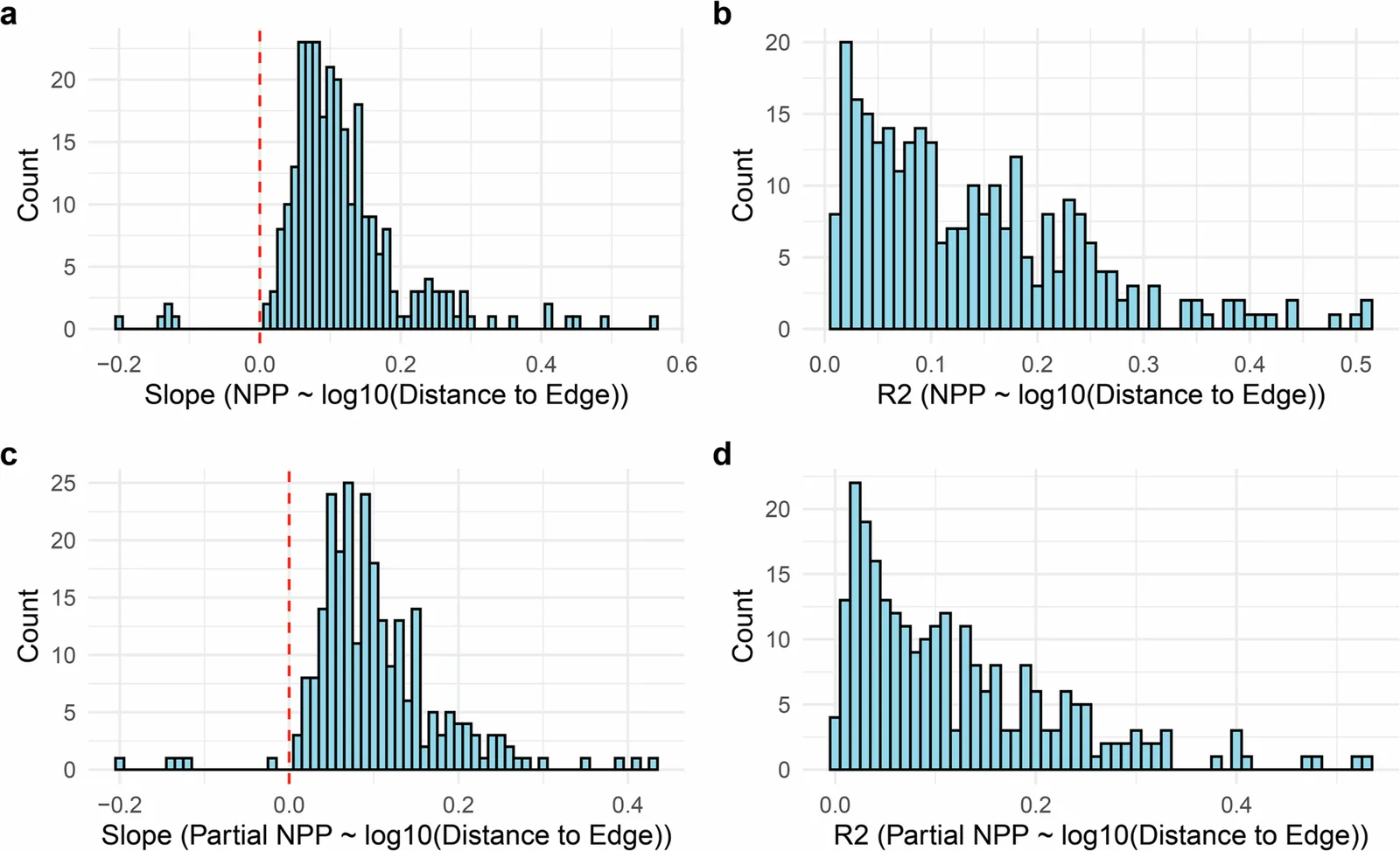
Histograms summarize the slope and coefficient of determination (R^2^) from patch-level regressions of forest productivity against log_10_(distance to forest edge) for all forest patches with ≥50 sampled pixels across the conterminous United States. Panels **a**–**b** show results using raw annual NPP, while panels **c**–**d** show results after detrending NPP for topographic variation (slope, elevation, and aspect). Red dashed lines in panels a and c indicate zero slope. Across patches, estimated slopes are generally positive, indicating higher NPP in interior forest pixels than edge pixels. Source data

A central mechanism linking forest patch size to productivity is the disproportionate influence of forest edges in small patches (Extended Data Table 1). Geometrically, smaller patches have much higher edge-to-area ratios than larger ones, so a larger fraction of their area is exposed to edge-associated conditions. Consistent with this expectation, edge exposure declines sharply with increasing patch size across CONUS (*R*^2^ = 0.71; Extended Data Fig. 1), indicating that large patches are increasingly dominated by interior forest environments. Analyses of productivity gradients within individual large patches further show that NPP generally increases with distance from the forest edge, both before and after detrending for topography (Extended Data Figs. 2and 3), although the strength of this relationship varies among patches. Together, these patterns suggest that edge-related stress, including hotter, drier and more variable microclimates^12,15,16,40^, reduces productivity near forest edges. Although localized positive edge effects have been reported in some temperate systems^41,42^, these effects are typically confined to within ~30 m of the edge and are unlikely to be detectable at the spatial resolution of our analysis. At the landscape scales examined here, negative edge effects, therefore, provide a parsimonious explanation for higher productivity in larger patches.

A complementary mechanism is biodiversity. Island biogeography theory predicts that larger patches support higher species richness than smaller, more isolated ones^43–45^ (Extended Data Table 1). If elevated biodiversity supports higher and/or more stable plant productivity, as suggested by biodiversity–ecosystem functioning research^46–48^, then larger forest patches should, on average, support greater vegetation density and higher NPP, consistent with our results. In plant communities, such gains can arise through sampling effects, where diverse communities have a higher probability of including highly productive species^49^, and through complementarity or compensatory dynamics among species^50^. Animal biodiversity can further enhance plant productivity by sustaining key interactions, including pollination and seed dispersal that facilitate recruitment, and by regulating herbivory through trophic controls^51^. Conversely, local extinctions following habitat loss and isolation^14^ may erode these biodiversity-mediated benefits in small patches, contributing to their lower productivity.

**Extended Data Fig. 4:**
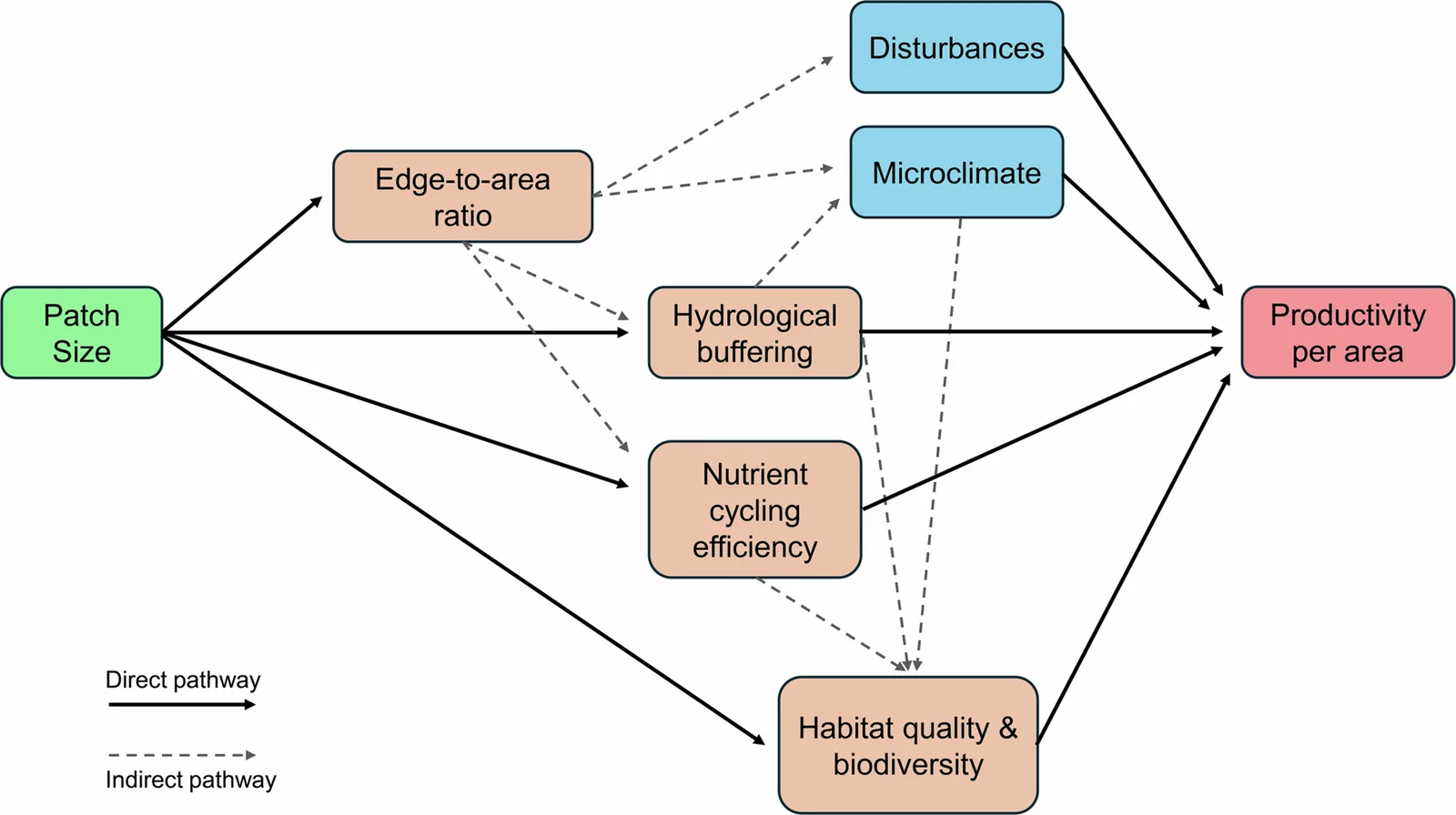
Conceptual pathways linking forest patch size to per-area productivity. The diagram summarizes direct and indirect mechanisms by which forest patch size influences productivity per unit area. Larger patches reduce the edge-to-area ratio, limiting exposure to edge-related microclimatic stress and disturbances^12,13,15,16,56^. Patch size also directly enhances hydrological buffering^76,77^, nutrient cycling efficiency^78–80^, and increases habitat quality and biodiversity^43–48^, which together regulate moisture availability, nutrient retention, and ecosystem functioning. Dashed arrows indicate indirect or cascading pathways mediated by edge effects, microclimate, and disturbance, while solid arrows represent direct effects. Collectively, these pathways provide a mechanistic framework linking forest fragmentation to reduced productivity in small patches.

The superlinear scaling we observe likely reflects the combined influence of multiple mechanisms (Extended Data Table 1 and Extended Data Fig. 4) that jointly increase mean NDVI and per-area NPP and GPP in large patches relative to small ones. Although our spatial random-effects approach accounts for spatial variation in socio-environmental conditions, we cannot exclude additional drivers operating at finer spatial scales. Quantifying the relative contribution of candidate mechanisms, including edge microclimates, demography and biodiversity, will require targeted analyses and field studies. Because edge metrics and edge delineation are scale-dependent^52^, it will also be important to test how patch-size scaling changes with spatial resolution, especially as very high-resolution satellite data become increasingly available^53^. Until these mechanisms are better constrained, process-based models are unlikely to reproduce the observed superlinear scaling and may, therefore, tend to underestimate the carbon-sequestration consequences of forest fragmentation

### Generality of patch size–productivity relationship

**Fig. 5:**
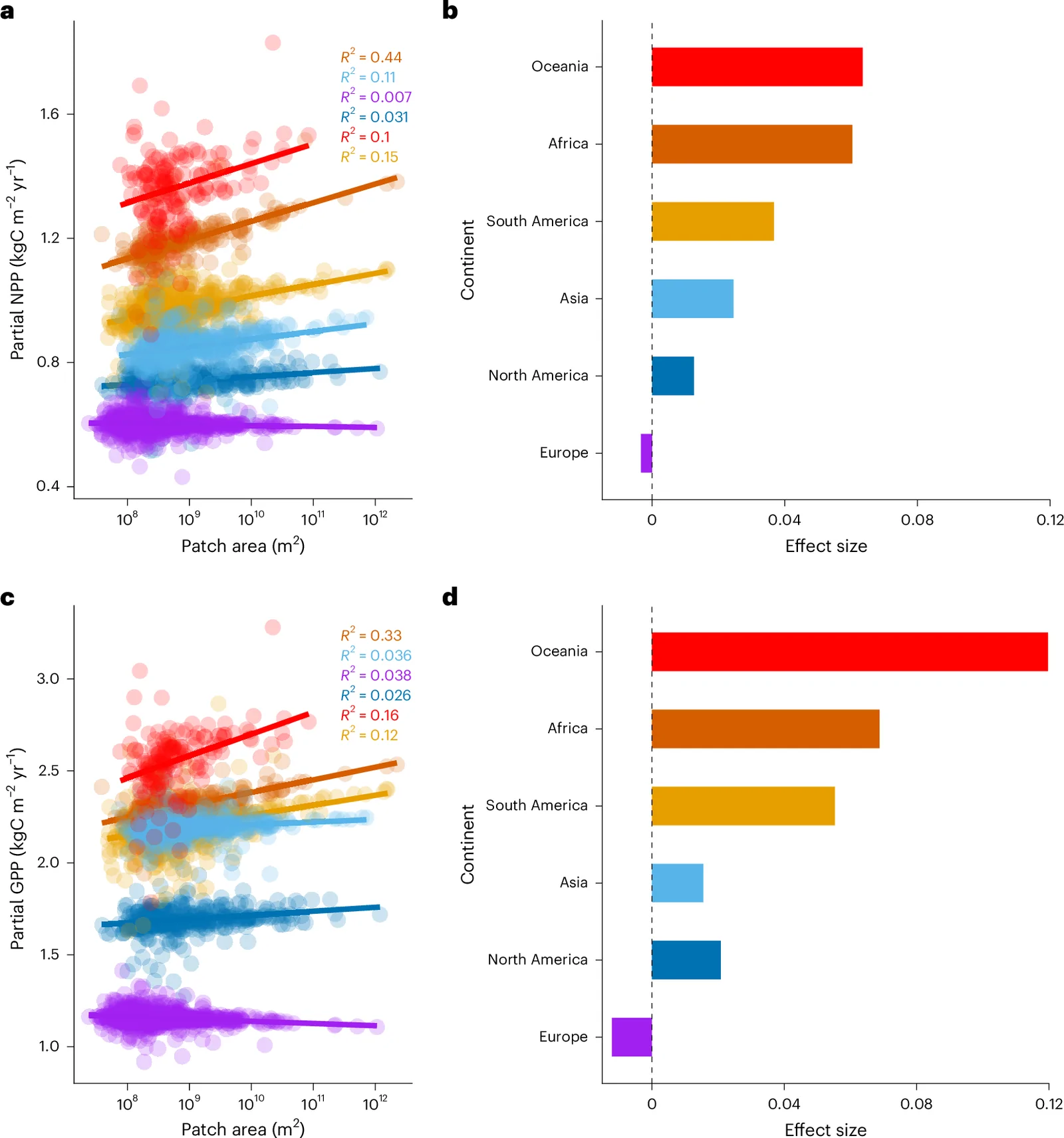
Patch size–productivity relationships across global forest patches. **a**, Relationships between partial NPP (MODIS NPP, 500-m resolution) and forest patch size for large forest patches worldwide (*n* = 2,818). Points show partial residuals from biome-specific mixed-effects models after controlling for broad environmental variation and spatial clustering; solid lines indicate fitted linear trends. Colours denote continents the same as in **b** and **d**, and annotated *R*^2^ values indicate the variance explained by patch size within each continent. **b**, Estimated effect sizes (standardized slopes) of log_10_(patch size) on NPP for each continent, with dashed vertical line indicating zero effect. **c**, As in **a**, but for MODIS GPP (500-m resolution). **d**, Corresponding effect sizes of log_10_(patch size) on GPP across continents. *P* values for all effect sizes shown in **b** and **d** are <0.001, where statistical significance was assessed using two-sided Wald tests of the regression coefficient. Source data

To assess whether the patch size–productivity relationship extends beyond the CONUS, we applied the same linear mixed-effects framework to global forest patches using 500-m moderate-resolution imaging spectroradiometer (MODIS) NPP and GPP products^54^. Across major forest biomes, we observed broadly consistent positive relationships between patch size and per-area productivity (Fig. 5 and Supplementary Figs. 3–8), indicating that superlinear scaling is a widespread feature of forest landscapes at broad spatial scales. The strength of this relationship varied among continents, with higher explanatory power in tropical-dominated continents, such as South America, Africa, Asia and Oceania (*R*^2^ ≈ 0.10–0.44), than in temperate regions including Europe and North America (*R*^2^ ≈ 0.007–0.031). Weak negative relationships emerged in a small number of regions, including temperate forests in Europe, tropical dry forests in South America and temperate broadleaf and mixed forests in Oceania, suggesting that in these systems factors other than fragmentation—such as land-use history or disturbance regimes^55^—may exert stronger controls on productivity. The mechanisms underlying these exceptions remain uncertain, highlighting the need for targeted regional studies to identify the conditions under which patch size ceases to enhance forest productivity.

The stronger patch size–productivity relationships observed in tropical regions likely reflect the heightened sensitivity of tropical forests to fragmentation. Tropical forests can experience particularly strong and spatially extensive edge effects^12,13,15,16,56^, including elevated temperatures, increased vapour pressure deficit and higher tree mortality, which can penetrate far into forest interiors and suppress carbon uptake in small patches (Extended Data Table 1). In addition, tropical forests may exhibit strong area–species–productivity relationships^43–48^: larger patches tend to support higher species richness and functional diversity, which enhance productivity through niche complementarity and more efficient resource use. By contrast, productivity in temperate forests is more strongly constrained by climate and may exhibit weaker or more localized edge responses^41,42^, reducing the relative influence of patch size. Together, these mechanisms may explain why patch size explains substantially more variation in NPP in tropical-dominated regions than in temperate ones.

### Conclusion

We show that annual per-area productivity increases with patch size and, consequently, that total productivity scales superlinearly with patch size. Further research is needed to evaluate the relative contributions of the mechanisms that may underlie this pattern. Nonetheless, our observational findings indicate that, in terms of carbon uptake, a hectare in a large, contiguous patch is more productive than a hectare in a small, isolated patch. As a consequence, preserving or restoring many small forest patches may not fully offset the loss of a single large patch of the same size. This highlights the limitations of area-only ‘net zero deforestation’ frameworks and the need for approaches that also account for forest configuration and ecosystem integrity^25^.

## Methods

### Geospatial dataset

Using Google Earth Engine^57^, we performed a ‘four-connected’ operation to vectorize the 30-m resolution 2010 forest cover raster map produced by Hansen et al.^24^, who measured forest cover by applying machine learning to high-resolution Landsat imagery. We classified pixels with 30% or greater tree canopy cover as ‘forest’, following ref. ^30^. Here ‘patch’ denotes a contiguous forested area (≥30% canopy; four-connected) from the Hansen map; we do not use ‘patch’ to mean disturbance gaps^58^. To ensure that no forest patches were inappropriately truncated by political borders, we first ran this operation across the North American continent rather than only the CONUS, delineating approximately 30 million forest patches in all. We calculated, within each one, the total productivity in 2010 based on the Landsat 30-m resolution remotely sensed dataset produced by Robinson et al.^35^; as this dataset covers only the CONUS, downstream filtering restricted our analysis to this region, which we recalculated. Beyond the availability of high-resolution productivity data, we selected the CONUS as the main study region for this work because its forests represent a significant portion of global temperate forest carbon sequestration, contributing substantially to global carbon sinks and climate regulation^59–61^. Furthermore, the diversity of forest types across CONUS provides a broad ecological context to assess the relationship between patch size and productivity, making findings widely generalizable^62,63^. Given its history of extensive land-use changes, forest fragmentation and ongoing restoration efforts, CONUS offers unique insights into how landscape structure affects productivity, with direct implications for global forest management policies^64,65^. To ensure that cloud contamination or data processing errors in the underlying Landsat NDVI dataset used in ref. ^35^ did not affect our findings, we filtered out pixels that used ≥25% gap-filled 16-day composites to calculate annual productivity. While even our quality-controlled Landsat NDVI and downstream data products are unlikely to be perfectly error-free, we are not aware of any reason why the errors should be correlated with forest patch size such that they would falsely produce our results. We assigned patches to different biomes following the modal biome categorization of pixels within the patch, following ref. ^63^.

We filtered out patches in which the percentage of total area for which we had productivity measurements was less than 75% or in which total productivity was calculated to be zero, as this indicates incomplete data product coverage. To calculate the total productivity of patches with at least 75% but less than 100% coverage of the productivity map, we calculated the per-unit area productivity of the covered portion, then multiplied it by the total area of the patch. We were left with 16,918,882 forest patches covering 1,472,585 km^2^ for use in downstream analyses, which we carried out using R^66^ and tidyverse^67^.

### Linear mixed-effects model framework

To quantify how productivity scales with patch size, we partitioned CONUS into 1° × 1° grid cells and fit linear mixed-effects models (R package lme4^68^) within each biome and for CONUS as a whole. For patch *i* in cell *j*, we modelled the outcome ${y}_{{ij}}$ (annual NPP per square metre, GPP per square metre, NDVI or other proxy for productivity) as

1 $${y}_{{ij}}={\beta }_{0}+{\beta }_{1}\times {\log }_{10}\left({\mathrm{PatchSize}}_{{ij}}\right)+{\mu }_{j}+{\varepsilon }_{{ij}\,}$$

where *β*_0_ is the fixed intercept, *β*_1_ is the fixed effect of log_10_-transformed patch size, ${\mu }_{j}$ is a cell-specific random intercept and ${\varepsilon }_{{ij}}$ is the residual. The random intercept absorbs broad socio-environmental differences shared by patches in the same cell (for example, regional climate, soils, potential vegetation, age structure, land-use history, management), so ${\beta }_{1}$ is identified by within-cell contrasts—that is, how per-area productivity changes with patch size after holding large-scale context constant. This simultaneously reduces spatial confounding and accounts for spatial clustering (non-independence) of nearby patches, which follows the logic of panel-data models in econometrics, where fixed or random effects are used to control for unobserved, location-specific variation (for example, country-level fixed effects^69^). We chose mixed-effects models because they (1) separate fixed and random components to target the within-cell estimand while capturing between-cell heterogeneity, (2) partially pool cell intercepts to stabilize estimates in sparsely sampled cells (more efficient than fitting thousands of fixed intercepts), (3) handle highly unbalanced sample sizes across cells and (4) explicitly model spatial clustering, reducing pseudo-replication.

### Outcome definition and transformation

In modelling annual NPP per square metre rather than total annual NPP, we sought to divide out the trivial linear relationship between quantity of area measured and total productivity. Though performing regressions of the form *y*/*x* ~ *x* has the potential to produce spurious correlations, we point out that if *y* and *x* are always positive (as in our case) and uncorrelated, such spurious correlations would have to be negative, not positive, as *y*/*x* should shrink as *x* grows. To calculate *P* values, we used the analysis of variance function in the car R package^70^ with type II sum of squares, which tests each main effect after accounting for all other main effects but without assuming interaction terms.

### Residual spatial autocorrelation

To test whether problematic spatial autocorrelation remained, we used the moranfast R package^71^ to calculate Moran’s *I* of model residuals within each biome. Because the calculation time of Moran’s *I* scales quadratically with dataset size, it is unfortunately not possible to calculate for the full 17-million-patch CONUS dataset. However, Moran’s *I* calculated within each biome indicated that only very minor positive spatial autocorrelation remained (<0.09, on a scale of 0–1, where zero is perfectly spatially uncorrelated and 1 is perfectly positively spatially autocorrelated; Supplementary Table 1).

### Counterfactual comparison (carbon consequences)

To quantify the potential consequences of forest fragmentation for aggregate carbon uptake, we conducted a counterfactual comparison that isolates the contribution of the patch size–productivity relationship while holding total forest area and broad environmental context constant. This analysis builds directly on the estimated relationship between forest patch size and per-area productivity and is intended to assess the magnitude of carbon sequestration differences attributable to landscape configuration rather than forest extent.

We first estimated mean per-area NPP (kgC m^−^^2^ yr^−1^) for different patch-size classes using our spatially explicit mixed-effects models (Table 1). These estimates provide empirical benchmarks for productivity associated with large, contiguous forest patches and small, highly fragmented patches under observed environmental conditions.

Using these benchmarks, we constructed three hypothetical landscape scenarios for the CONUS: (1) least-fragmented scenario—all forested area is assumed to function as large, contiguous patches and is assigned a uniform per-area productivity equal to the mean NPP of the largest observed patch-size class (~10^11^ m^2^); (2) realized scenario—total NPP is calculated using the observed landscape configuration by summing, across all patches, the product of each patch’s area and its empirically estimated per-area NPP; and (3) most-fragmented scenario—all forested area is assumed to function as small, highly fragmented patches and is assigned a uniform per-area productivity equal to the mean NPP of the smallest observed patch-size class (~10^4^ m^2^).

In all scenarios, total forest area is held constant, and differences in total annual NPP, therefore, arise solely from differences in assumed per-area productivity associated with forest patch size. Total NPP for each scenario was calculated by multiplying per-area productivity by forest area and summing across all patches, with results expressed in gigatons of carbon per year (GtC yr^−1^).

This counterfactual framework does not represent a literal historical or future landscape trajectory, but rather brackets the potential upper and lower bounds of carbon sequestration associated with forest configuration alone. By comparing the realized landscape to these bounding scenarios, we estimate the magnitude of carbon uptake that may be gained or lost due to fragmentation independently of changes in total forest cover.

### Random forest model for showing the importance of patch size

To evaluate the relative importance of forest patch size in explaining spatial variation in forest productivity, we implemented a random forest regression analysis as a complementary, non-parametric approach. This analysis was designed to assess whether patch size contributes explanatory power beyond commonly recognized climatic, topographic and edaphic controls, without making causal claims.

We randomly sampled 239,633 forest pixels across the CONUS (see Supplementary Fig. 9 for spatial distribution of sampled pixels), restricted to pixels classified as forest (cover ≥30%) in the 2010 Hansen forest cover product. For each sampled pixel, we extracted annual NPP (kgC m^−^^2^ yr^−1^) and a suite of environmental covariates that are known as important predictors of NPP^31–34^, including mean annual temperature, mean annual precipitation, slope, aspect, soil pH and soil carbon-to-nitrogen ratio (Supplementary Table 2, variance inflation factor < 3 for all covariates). Each pixel was also assigned the log_10_-transformed area of the forest patch to which it belonged, allowing patch size to be evaluated alongside local environmental predictors.

We fit random forest regression models using the ranger package in R, with NPP as the response variable. Two otherwise identical models were trained: one including log_10_(patch size) as a predictor and one excluding patch size. Explanatory power was quantified using the coefficient of determination (*R*^2^) between observed and predicted NPP.

To assess the relative contribution of each predictor, we computed permutation-based variable importance, which measures the increase in prediction error when a given predictor is randomly permuted while all others are held constant. This approach accounts for nonlinear effects and interactions among predictors and provides a robust ranking of variable importance without assuming a specific functional form. Patch size importance was evaluated relative to climatic, topographic and soil variables to determine whether landscape configuration contributes meaningfully to explaining forest productivity patterns at the pixel scale.

### Assessing the role of forest edges

To assess whether forest edge effects may help explain the link between forest fragmentation and reduced productivity, we conducted a set of complementary geometric and pixel-based analyses using the same dataset employed in the random forest models, comprising ~240,000 randomly sampled forest pixels across the CONUS.

First, we quantified the geometric relationship between forest patch size and edge exposure. For each delineated forest patch, we calculated the edge-to-area ratio, defined as the length of patch boundary divided by patch area. This metric provides a scale-independent measure of the proportion of forest area influenced by edges. We then examined the relationship between patch size (m^2^, log_10_-transformed) and edge-to-area ratio across all forest patches using ordinary least squares regression (Extended Data Fig. 1), allowing us to assess how edge dominance scales with fragmentation due to geometry alone.

Second, to test for within-patch edge effects on productivity, we analysed the relationship between forest productivity and distance to the nearest forest edge at the pixel level. For the ten largest forest patches in CONUS, we regressed annual NPP (kgC m^−^^2^ yr^−1^) against log_10_(distance to edge, m) for all forest pixels within each patch (Extended Data Fig. 2). To isolate edge-related effects from confounding environmental gradients, NPP values were detrended prior to analysis by removing the effects of topographic covariates (slope, elevation and aspect) using linear regression; residuals from these models were used as topography-adjusted NPP. Linear fits are shown for visualization only.

Third, to generalize edge effects across the landscape, we repeated the pixel-level regression of productivity against log_10_(distance to edge) for all forest patches containing at least 50 sampled pixels. For each patch, we estimated the slope and coefficient of determination (*R*^2^) of the relationship using both raw annual NPP and topography-detrended NPP. The distributions of regression slopes and *R*^2^ values across patches are summarized as histograms (Extended Data Fig. 3). Positive slopes indicate higher productivity in interior forest pixels relative to edge pixels. Red dashed lines denote zero slope, providing a reference for the directionality of edge effects.

Together, these analyses test whether (1) smaller patches are geometrically dominated by edge environments and (2) forest edges are associated with reduced productivity relative to interiors at spatial scales relevant to our analysis, thereby linking fragmentation to declines in per-area forest productivity.

### Global large-patch analysis (NPP and GPP)

To test whether the patch-size–productivity relationship observed in CONUS holds more broadly, we extended the analysis to global forests. Global forest patches (Supplementary Fig. 10) were delineated on Google Earth Engine using the same four-connected operation on the 2010 Hansen forest cover map (≥30% canopy) and assigned to biomes based on the classic Olson class^62,63^ within each patch.

We analysed two productivity metrics at the global scale: MODIS-derived NPP and MODIS-derived GPP, using globally available annual products^54,72–74^ harmonized to the study year 2010. MODIS NPP and GPP are derived from optical sensors at 500-m resolution. We applied an edge-exclusion filter by removing NPP and GPP pixels with <95% forest cover to reduce contamination from non-forest areas. This resulted in a dataset with 2,818 large forest patches. All products were quality-filtered using standard quality flags and aggregated to patch means after removing edge pixels.

The same linear mixed-effects model specification was applied globally, fitting models both within each biome with at least 50 large patches and across all global forests. The fixed effect was log_10_-transformed patch size (m^2^), and the random intercept was the 1° × 1° global grid cell containing the patch centroid, thereby controlling for broad-scale environmental differences and spatial clustering. Type II tests were used to assess the patch-size effect, and Moran’s I was computed within each biome to quantify residual spatial autocorrelation. For comparability across biomes, we standardized patch-size effects by holding all other predictors at their biome-specific means and removing random effects from predictions (Fig. 5 and Supplementary Figs. 3–8).

### Reporting summary

Further information on research design is available in the Nature Portfolio Reporting Summary linked to this article.

## Supplementary information

Supplementary Information Supplementary Methods, Figs. 1–12 and Tables 1 and 2.

Reporting Summary

Peer Review File

Supplementary Table Summaries of NPP, GPP, NDVI and tree cover models, with p-value and beta coefficient for log10-transformed patch size, as well as marginal (not including variation explained by the spatial random effect) R^2^ for the model and Moran’s I of residuals for each individual biome and each analysis. The full CONUS dataset is too large for computation of Moran’s I.

## Source data

Source Data Fig. 1 Statistical source data.

Source Data Fig. 2 Statistical source data.

Source Data Fig. 3 Statistical source data.

Source Data Fig. 4 Statistical source data.

Source Data Fig. 5 Statistical source data.

Source Data Extended Data Fig. 1 Statistical source data.

Source Data Extended Data Fig. 2 Statistical source data.

Source Data Extended Data Fig. 3 Statistical source data.

## Figures and Tables

**Extended Data Table 1:**
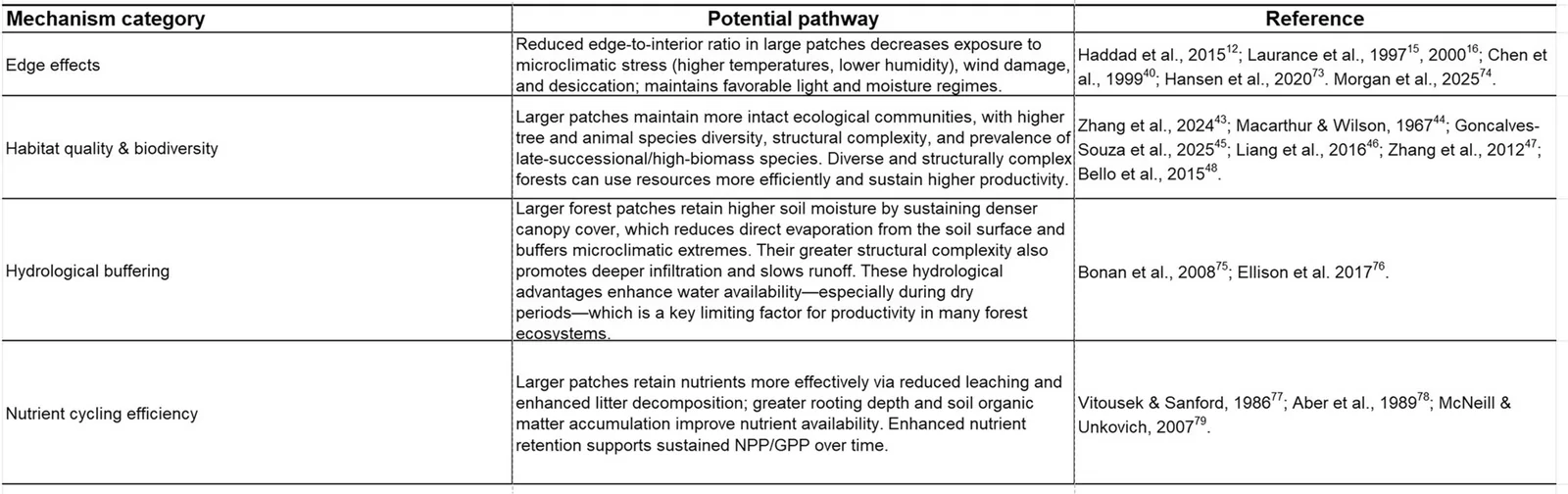
Potential mechanisms and pathways underlying the superlinear scaling of productivity with forest patch size

Summary of hypothesized pathways through which larger forest patches may sustain higher net primary productivity (NPP) and gross primary productivity (GPP) per unit area. Mechanisms shown include reduced edge exposure, greater habitat quality and biodiversity, stronger hydrological buffering, and more efficient nutrient cycling. References provide representative empirical and theoretical support for each pathway.

## Data Availability

The datasets owned by the authors and used in the analyses herein are available via Zenodo at https://doi.org/10.5281/zenodo.19134763 (ref. ^75^). Source data are provided with this paper.

## Code availability

The R code used in the analyses herein is available via Zenodo at https://doi.org/10.5281/zenodo.19134763 (ref. ^75^).
