# Supplementary material for: Larger forest patches have greater per-area productivity
Source: Nat Ecol Evol. 2026 May 15;10(7):1254–64. doi: 10.1038/s41559-026-03075-5 (PMC13649605; doi:10.1038/s41559-026-03075-5)
Supplement: Supplementary file 1 [file 41559_2026_3075_MOESM1_ESM.pdf]

nature ecology & evolution

Supplementary information https://doi.org/10.1038/s41559-026-03075-5

Larger forest patches have greater per-area productivity

In the format provided by the authors and unedited

Supplementary Methods

Independent productivity proxy: Vegetation Optical Depth (VOD)

To address potential dependence between Landsat-based productivity metrics (NPP, GPP, NDVI) and Landsat-based patch size—arising from their shared reliance on Landsat-based optical reflectance—we repeated the analysis using Vegetation Optical Depth (VOD)[1], a microwave-derived proxy for canopy water content and biomass that is physically and algorithmically independent of Landsat-based optical products. We used a 9-km resolution VOD product[2] and calculated annual means for the same study year as the Landsat-based data. Because the native resolution is coarse, we restricted the sample to forest patches in CONUS with area ≥ 81,000,000 m^2 (81 km^2), ensuring that each retained patch spanned at least one full VOD pixel. For patches overlapping multiple pixels, we excluded pixels falling on patch boundaries (with forest cover <95%) and then calculated the mean VOD value from the interior pixels.

We re-fit the linear mixed-effects model used in the main text, with annual mean VOD as the response variable. The fixed effect was $\log_{10}$-transformed patch size (m^2), and the random intercept was the 1° × 1° grid cell containing the patch centroid, controlling for broad-scale spatial heterogeneity (e.g., climate, soils, management, forest age) and accounting for spatial autocorrelation among nearby patches. Type II tests from the car R package were used to assess the size effect, and Moran's I was calculated to evaluate residual spatial autocorrelation. For biome-level visualization, we removed random effects to display within-cell relationships between patch size and mean VOD (Fig. S2).

Power law fitting and relating total productivity to α

To derive a power-law relationship describing the distribution of patch sizes, we used the method of White *et al*[3]. First, we generated the cumulative distribution function (CDF) of our dataset by ranking its values in ascending order then using the Kaplan-Meier estimate to calculate the probability that a given observation was less than or equal to each value. We used linear regression

to fit a linearization of the Pareto CDF to this CDF of our data[3], then derived α, the Pareto shape parameter, from the negative slope.

To quantify the distinct link between forest patch size distribution and productivity created by the superlinear scaling relationship, we derived a closed-form expression describing the total productivity of set of forest patches following a bounded Pareto distribution. We first calculated annual total NPP per patch as $x\,(\beta_0 + \beta_1 \log_e x)$, where x is patch size in m^2, β_0 is the linear regression intercept, and β_1 is the coefficient of the patch size-annual NPP m^{-2} relationship divided by $\log_e 10$ to account for the change of base in the logarithm. Then, we used the probability density function for the bounded Pareto distribution, $f_A(x) = \frac{\alpha k^{\alpha} x^{-(\alpha+1)}}{1-\left(\frac{k}{\ell}\right)^{\alpha}}$, where α is the shape parameter, k is the minimum patch size, and ℓ is the maximum patch size, to calculate an area-weighted average of annual forest NPP. This yielded the following integral expression:

$$\frac{\int_k^{\ell} (\beta_0 + \beta_1 \log_e x)\, x f_A(x) dx}{\int_k^{\ell} x f_A(x) dx} = \beta_0 - \frac{\beta_1}{1-\alpha} + \beta_1 \log_e k + \frac{\beta_1 \log_e\left(\frac{\ell}{k}\right)}{1-\left(\frac{k}{\ell}\right)^{1-\alpha}} \tag{2}$$

This expression describes a weighted average of annual NPP over the entire distribution of forest patch sizes and is independent of the total forested area. It can therefore be used to estimate the distinct effect on total NPP per year of changing the Pareto shape parameter, α, without changing the total quantity of forested area.

By doing so, we were able to calculate that superlinear scaling of productivity with forest size allows α to influence total annual NPP by itself, even if total forested area stays the same (Fig. S11b; Equation 1). In fact, the current α value, 0.69, positions the CONUS such that additional forest fragmentation is by itself expected to drive increasingly large marginal losses in total NPP (Fig. S11b). We estimate, accordingly, that a 50% decrease in α (*i.e.* less fragmentation) would raise total annual NPP by about 3%, corresponding to a yearly gain of about 45 Mt carbon[4], but that a 50% increase in α (*i.e.* more fragmentation) across the CONUS would reduce total annual NPP by over 13%, corresponding to a yearly loss of approximately 207 Mt carbon[4]. This is expected even in the absence of any change in total forested area.

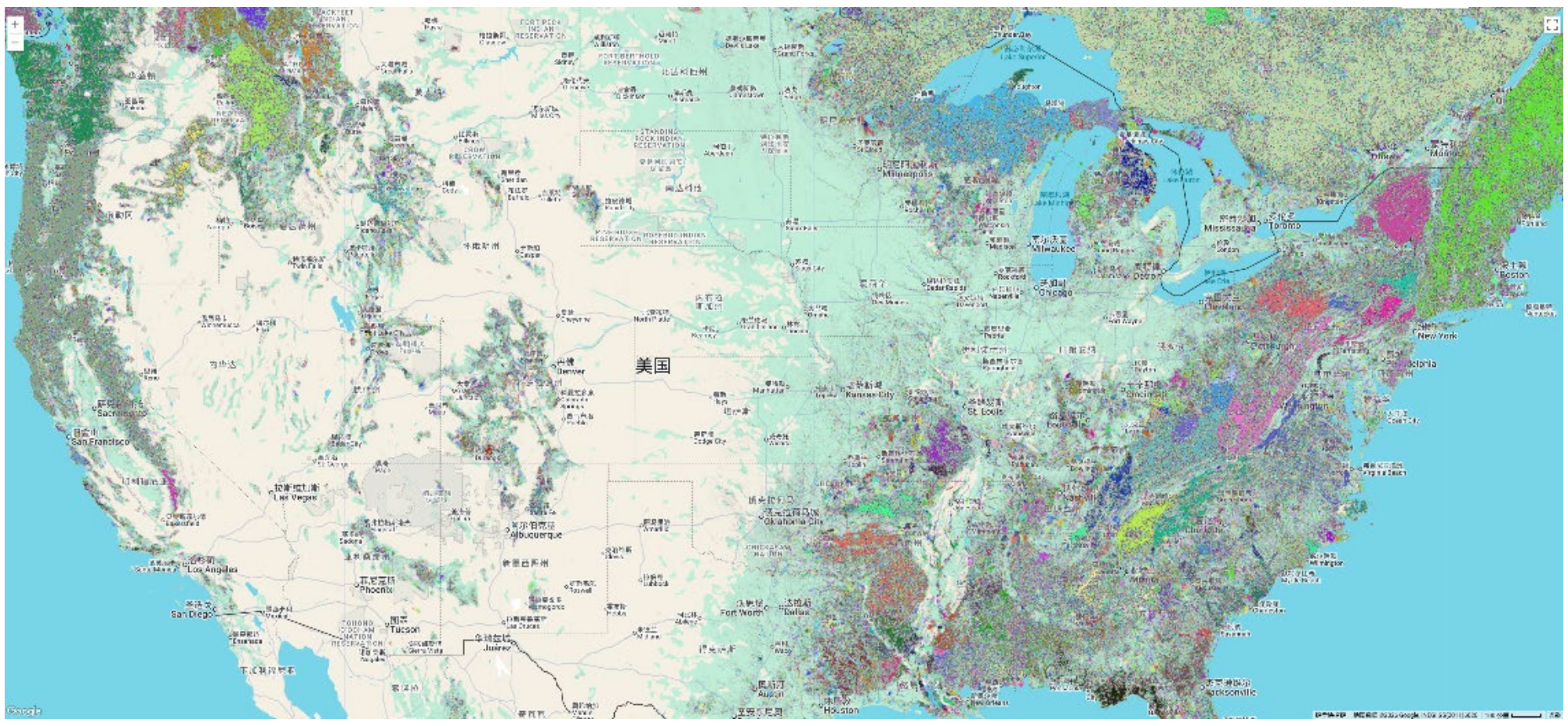

Supplementary Figure 1: Spatial distribution of forest patches across the conterminous United States (CONUS) derived from Google Earth Engine. Each color denotes an individual forest patch. Map data ©2026 Google.

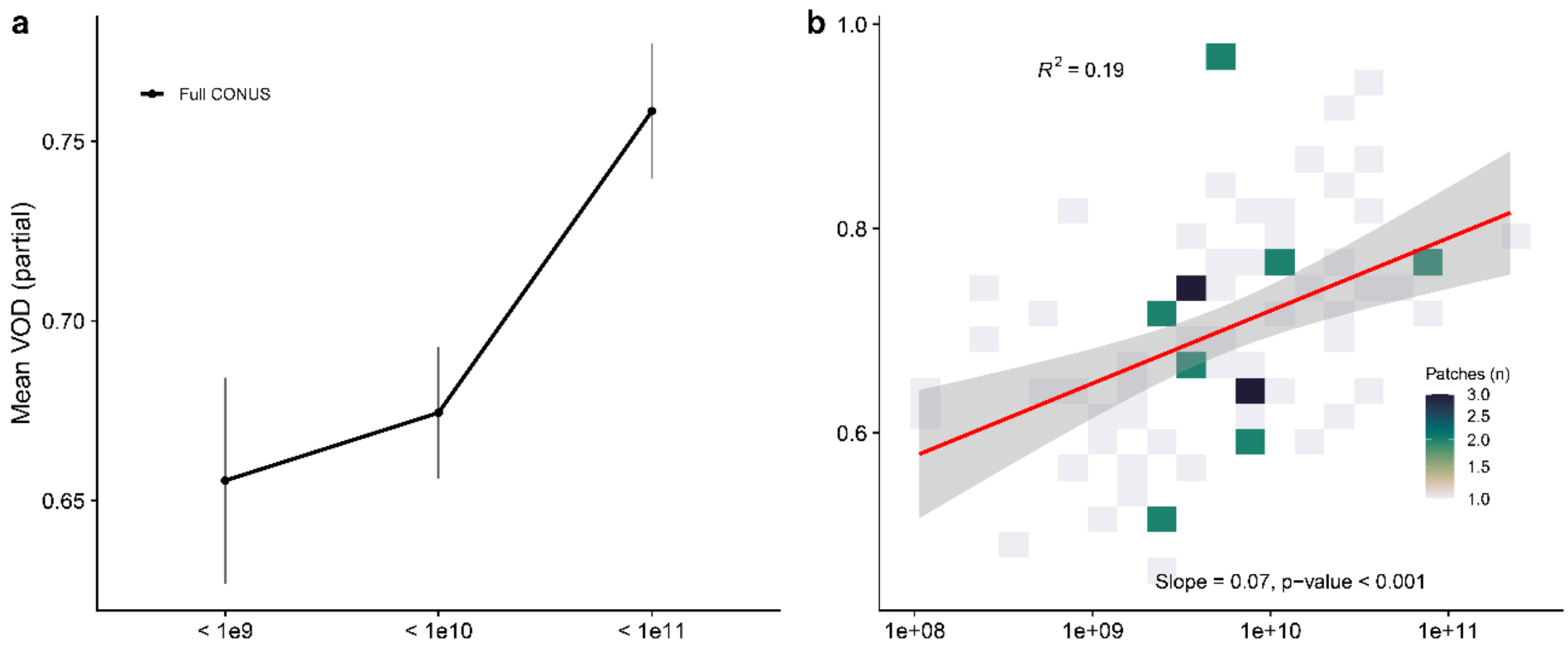

Supplementary Figure 2: Biome-specific a) mean VOD in large forest patches (size > 81000000 m^2, n = 73 for full CONUS), controlling for intrabiome variation in socio-environmental conditions and spatial autocorrelation (Methods). Error bars represent standard errors. We do not examine biomes with fewer than 50 forest patches (only the full CONUS fulfils this condition), nor do we render points for patch size classes with fewer than three observations. Here, e refers to base ten used in the logarithm. b) mean VOD, controlling for socio-environmental conditions and spatial autocorrelation as in panels a, of forest patches in the full CONUS rendered as a function of patch size, with a fitted

regression line for visualization purposes (n = 73). Values can be outside the bounds of the measurement because they are partial residuals.

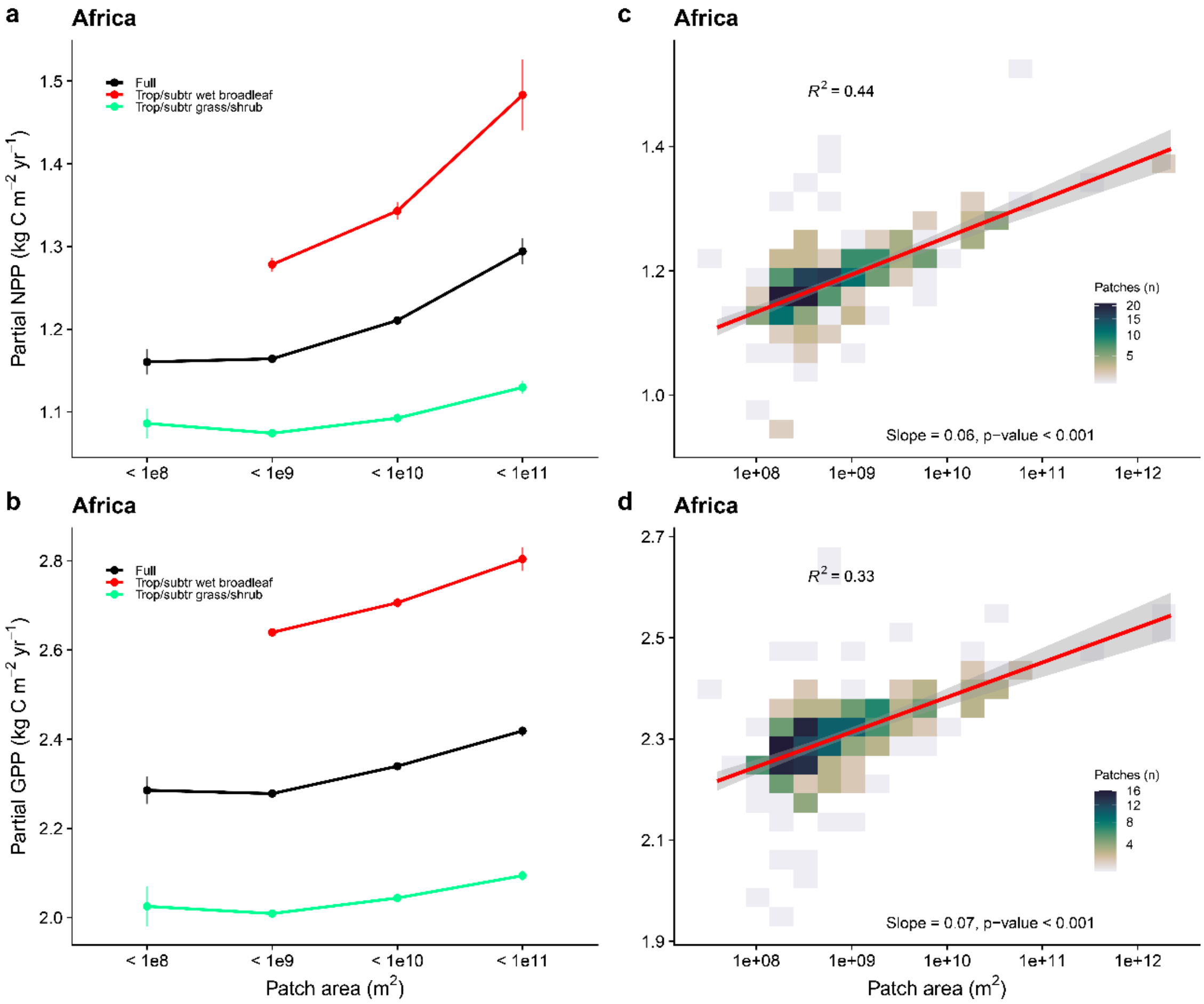

Supplementary Figure 3: Biome-specific a) annual NPP m-2, and b) annual GPP m-2 in large forest patches (n = 231 for full Africa), controlling for intrabiome variation in socio-environmental conditions and spatial autocorrelation (Methods). Data are presented as mean values +/- SEM. We do not examine biomes with fewer than 50 forest patches, nor do we render points for patch size classes with fewer than three observations. Here, e refers to base ten used in the logarithm. c) Annual NPP m-2, d) annual GPP m-2, controlling for socio-environmental conditions and spatial autocorrelation as in panels a – b, of forest patches in the full Africa rendered as a function of patch size, with a fitted regression line for visualization purposes (n = 231). The shaded regions around the linear regression line represent the 95% confidence interval for the predicted mean values. Slopes correspond to the estimated fixed-effect coefficient of $\log_{10}$(patch size) from linear mixed-effects models, and statistical significance was assessed using two-sided Wald tests of the regression coefficient. Values can be outside the bounds of the measurement because they are partial residuals.

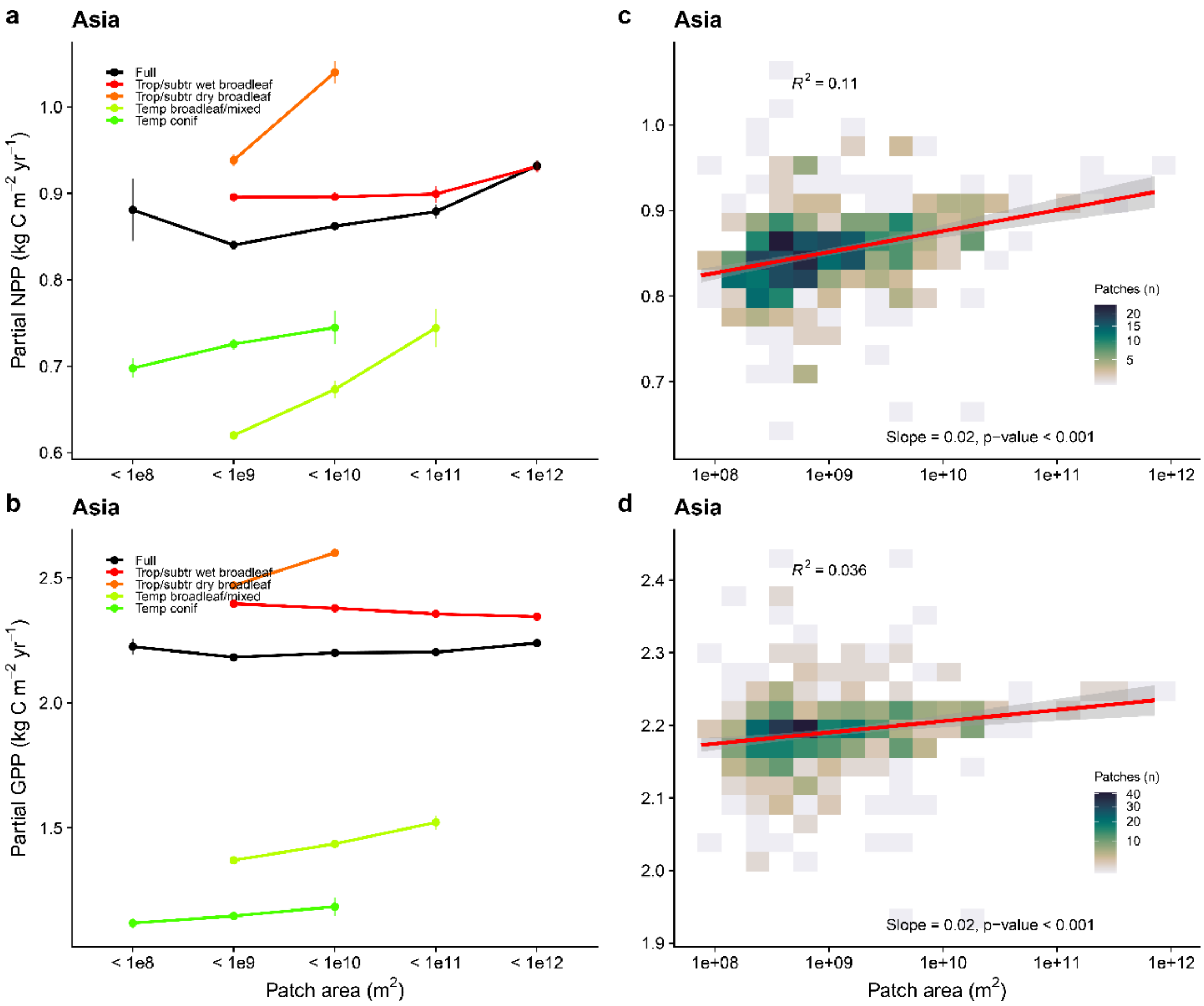

Supplementary Figure 4: Biome-specific a) annual NPP m-2, and b) annual GPP m-2 in large forest patches (n = 495 for full Asia, excluding Russia), controlling for intrabiome variation in socio-environmental conditions and spatial autocorrelation (Methods). Data are presented as mean values +/- SEM. We do not examine biomes with fewer than 50 forest patches, nor do we render points for patch size classes with fewer than three observations. Here, e refers to base ten used in the logarithm. c) Annual NPP m-2, d) annual GPP m-2, controlling for socio-environmental conditions and spatial autocorrelation as in panels a – b, of forest patches in the full Asia rendered as a function of patch size, with a fitted regression line for visualization purposes (n = 495). The shaded regions around the linear regression line represent the 95% confidence interval for the predicted mean values. Slopes correspond to the estimated fixed-effect coefficient of $\log_{10}$(patch size) from linear mixed-effects models, and statistical significance was assessed using two-sided Wald tests of the regression coefficient. Values can be outside the bounds of the measurement because they are partial residuals.

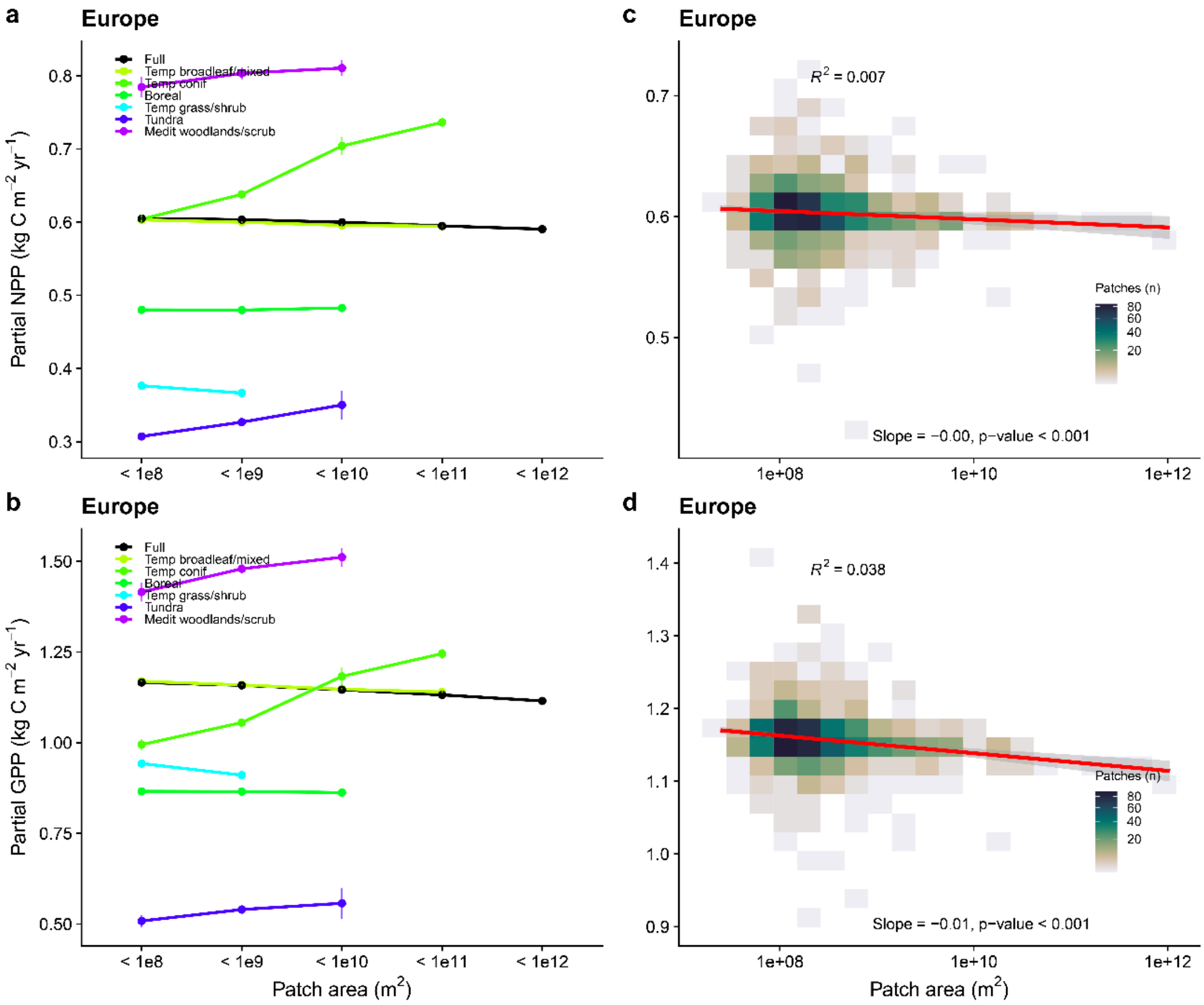

Supplementary Figure 5: Biome-specific a) annual NPP m-2, and b) annual GPP m-2 in large forest patches (n = 930 for full Europe, including Russia), controlling for intrabiome variation in socio-environmental conditions and spatial autocorrelation (Methods). Data are presented as mean values +/- SEM. We do not examine biomes with fewer than 50 forest patches, nor do we render points for patch size classes with fewer than three observations. Here, e refers to base ten used in the logarithm. c) Annual NPP m-2, d) annual GPP m-2, controlling for socio-environmental conditions and spatial autocorrelation as in panels a – b, of forest patches in the full Europe rendered as a function of patch size, with a fitted regression line for visualization purposes (n = 930). The shaded regions around the linear regression line represent the 95% confidence interval for the predicted mean values. Slopes correspond to the estimated fixed-effect coefficient of $\log_{10}$(patch size) from linear mixed-effects models, and statistical significance was assessed using two-sided Wald tests of the regression coefficient. Values can be outside the bounds of the measurement because they are partial residuals.

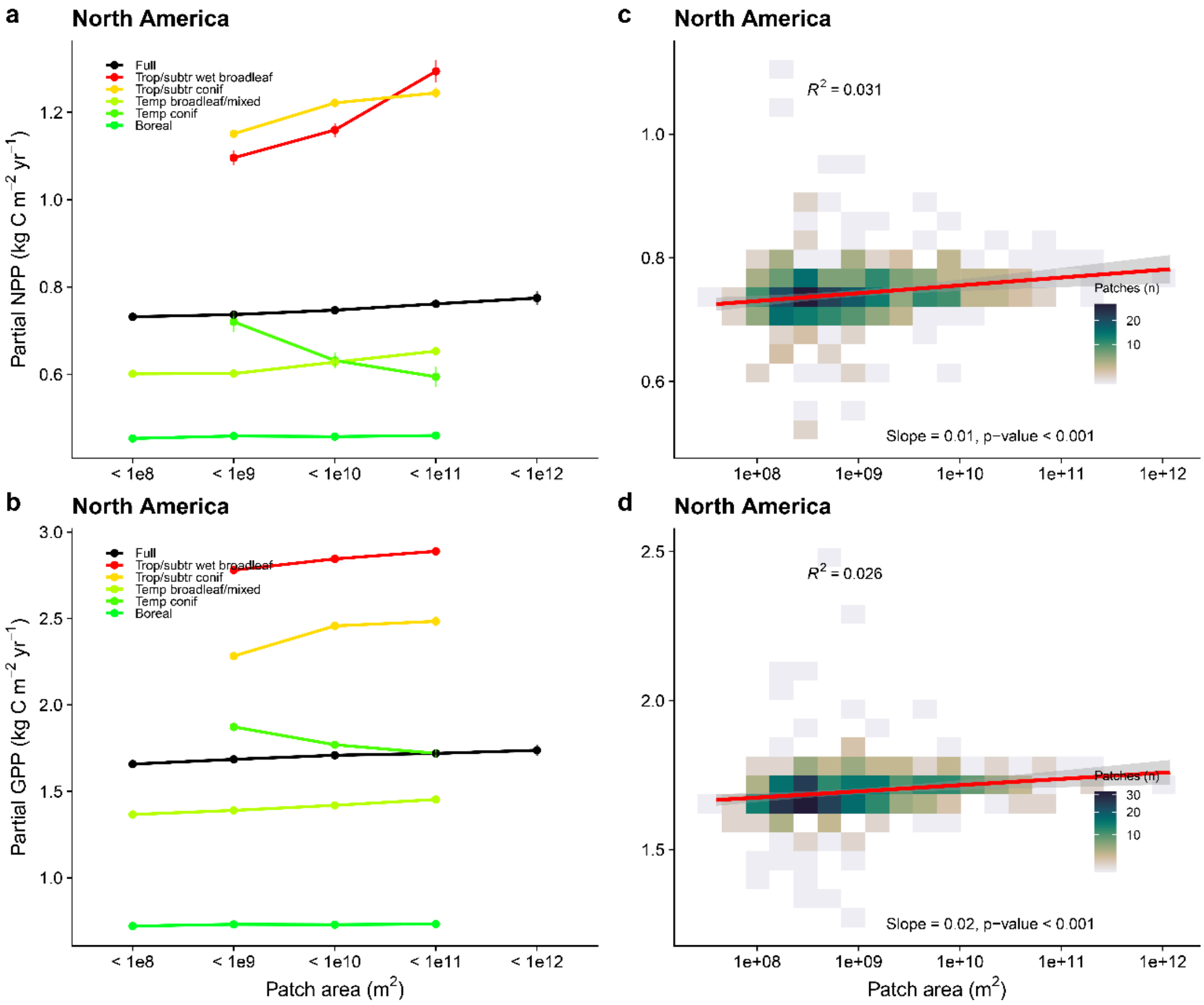

Supplementary Figure 6: Biome-specific a) annual NPP m-2, and b) annual GPP m-2 in large forest patches (n = 381 for full North America), controlling for intrabiome variation in socio-environmental conditions and spatial autocorrelation (Methods). Data are presented as mean values +/- SEM. We do not examine biomes with fewer than 50 forest patches, nor do we render points for patch size classes with fewer than three observations. Here, e refers to base ten used in the logarithm. c) Annual NPP m-2, d) annual GPP m-2, controlling for socio-environmental conditions and spatial autocorrelation as in panels a – b, of forest patches in the full North America rendered as a function of patch size, with a fitted regression line for visualization purposes (n = 381). The shaded regions around the linear regression line represent the 95% confidence interval for the predicted mean values. Slopes correspond to the estimated fixed-effect coefficient of $\log_{10}$(patch size) from linear mixed-effects models, and statistical significance was assessed using two-sided Wald tests of the regression coefficient. Values can be outside the bounds of the measurement because they are partial residuals.

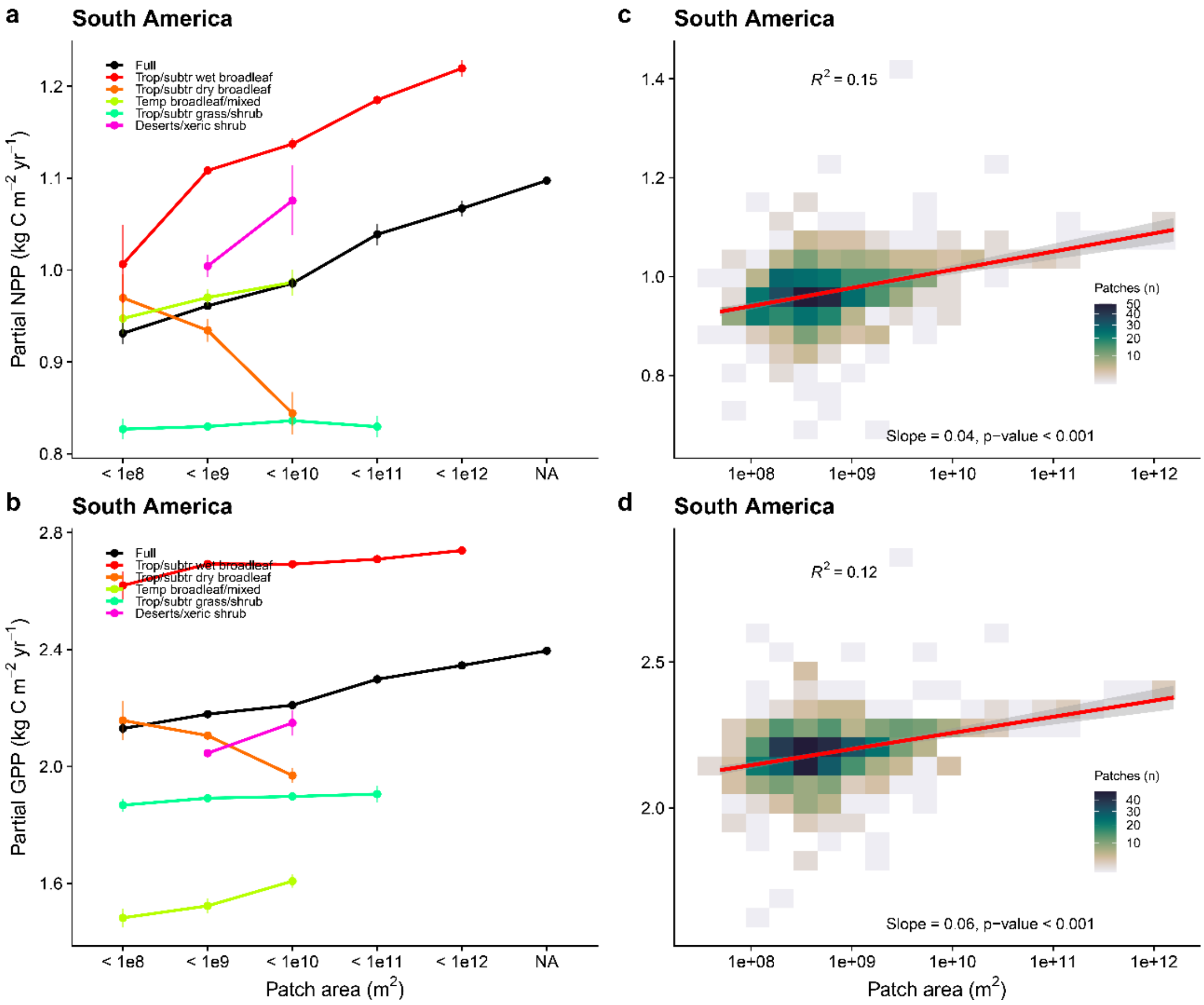

Supplementary Figure 7: Biome-specific a) annual NPP m-2, and b) annual GPP m-2 in large forest patches (n = 625 for full South America), controlling for intrabiome variation in socio-environmental conditions and spatial autocorrelation (Methods). Data are presented as mean values +/- SEM. We do not examine biomes with fewer than 50 forest patches, nor do we render points for patch size classes with fewer than three observations. Here, e refers to base ten used in the logarithm. c) Annual NPP m-2, d) annual GPP m-2, controlling for socio-environmental conditions and spatial autocorrelation as in panels a – b, of forest patches in the full South America rendered as a function of patch size, with a fitted regression line for visualization purposes (n = 625). The shaded regions around the linear regression line represent the 95% confidence interval for the predicted mean values.Slopes correspond to the estimated fixed-effect coefficient of $\log_{10}$(patch size) from linear mixed-effects models, and statistical significance was assessed using two-sided Wald tests of the regression coefficient. Values can be outside the bounds of the measurement because they are partial residuals.

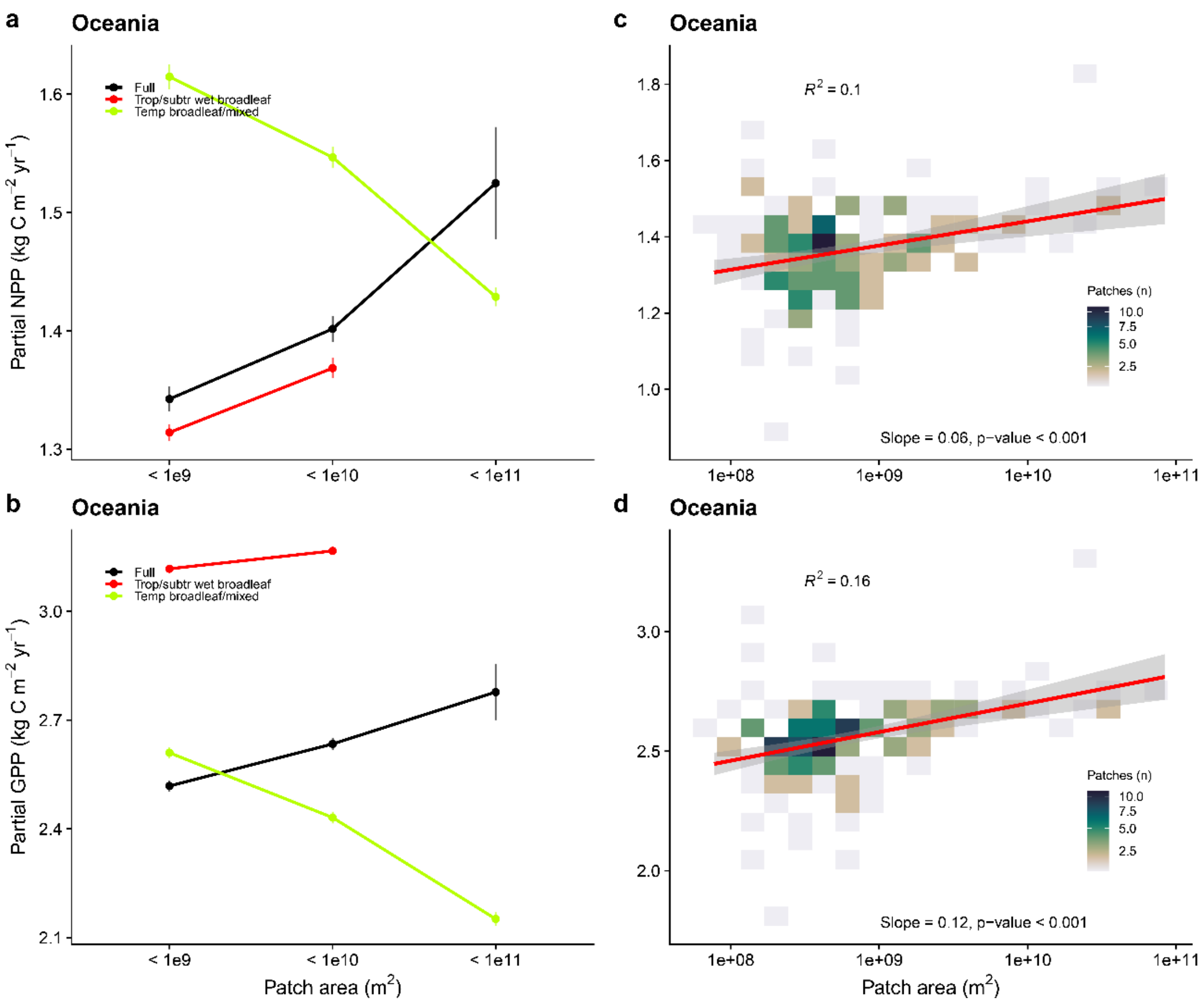

Supplementary Figure 8: Biome-specific a) annual NPP m-2, and b) annual GPP m-2 in large forest patches (n = 156 for full Oceania), controlling for intrabiome variation in socio-environmental conditions and spatial autocorrelation (Methods). Data are presented as mean values +/- SEM. We do not examine biomes with fewer than 50 forest patches, nor do we render points for patch size classes with fewer than three observations. Here, e refers to base ten used in the logarithm. c) Annual NPP m-2, d) annual GPP m-2, controlling for socio-environmental conditions and spatial autocorrelation as in panels a – b, of forest patches in the full Oceania rendered as a function of patch size, with a fitted regression line for visualization purposes (n = 156). The shaded regions around the linear regression line represent the 95% confidence interval for the predicted mean values. Slopes correspond to the estimated fixed-effect coefficient of $\log_{10}$(patch size) from linear mixed-effects models, and statistical significance was assessed using two-sided Wald tests of the regression coefficient. Values can be outside the bounds of the measurement because they are partial residuals.

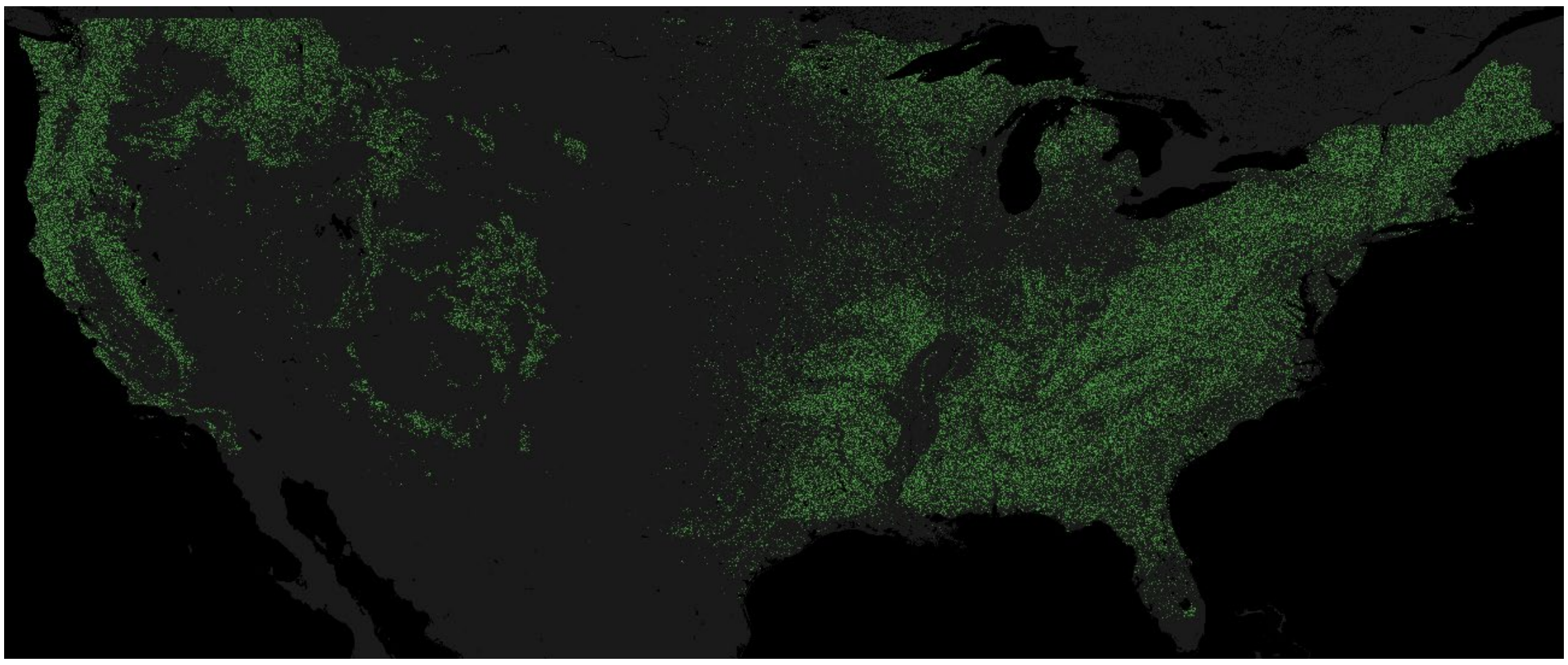

Supplementary Figure 9: Green points show the locations of the randomly sampled 239,633 30 m × 30 m forest pixels across the CONUS used in the random forest models and edge-effect analyses. Sampling spans all major forested regions, ensuring broad geographic and environmental coverage for model inference.

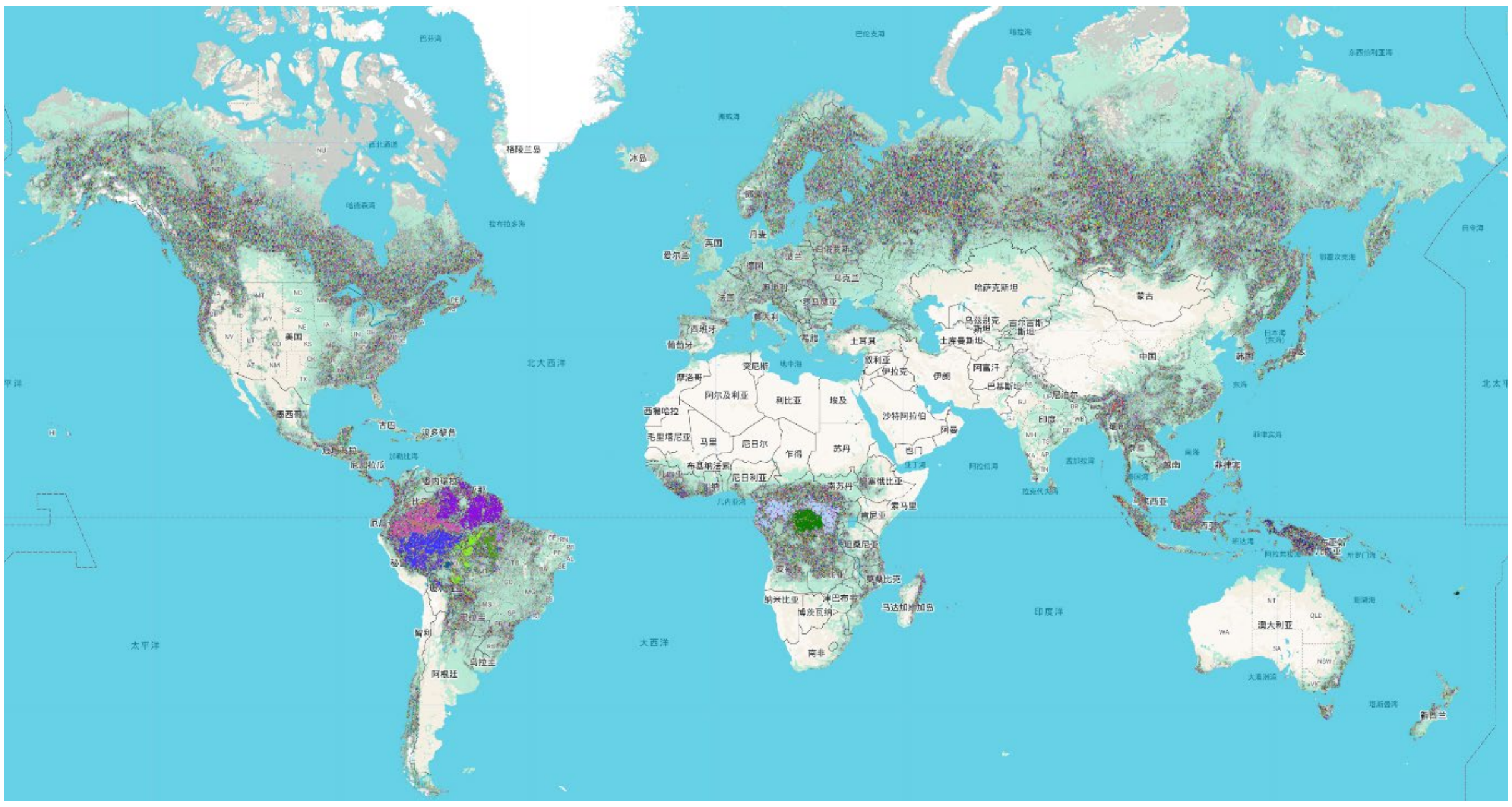

Supplementary Figure 10: Global distribution of forest patches derived from Google Earth Engine. Each color represents an individual forest patch. Patches shown are filtered according to the criteria described in *Methods* for the global analysis. Map data ©2026 Google.

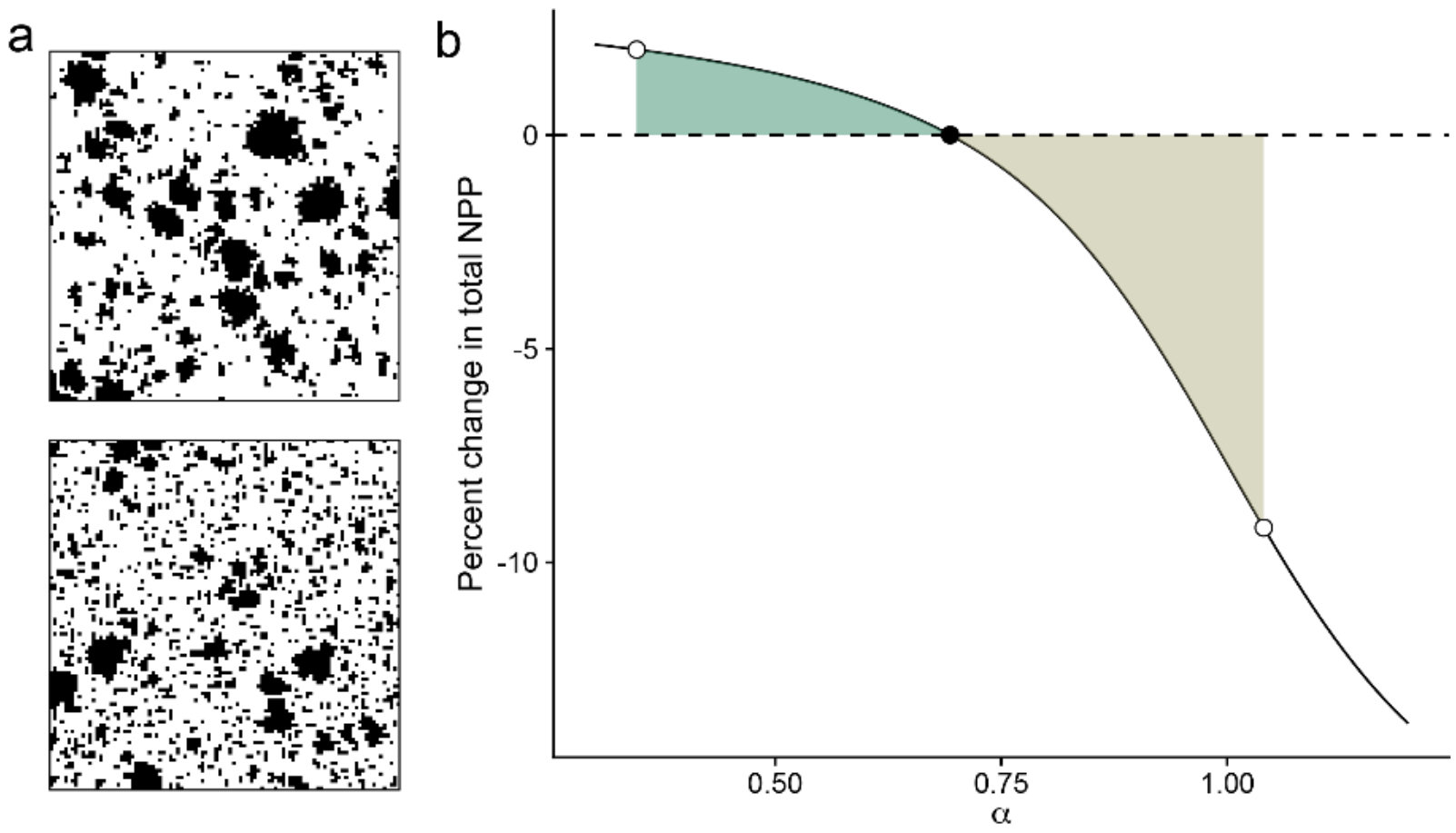

Supplementary Figure 11: a) Two hypothetical landscapes with the same total quantity of filled pixels (25%), but with patch sizes drawn from Pareto distributions with differing α values. The distribution of the top landscape has the same α as the full CONUS, 0.69, while the bottom has an α 50% larger (1.04). On a log–log plot, the slope of the patch-size CCDF equals −α; larger α indicates a steeper decline in large patches and, given a positive size–productivity slope, a lower total NPP at fixed area. b) Relationship of the Pareto shape parameter, α, to total NPP when the total quantity of forested area remains constant. The filled point represents the observed α value for the full CONUS, 0.69, while the empty points represent this α raised or lowered by 50%.

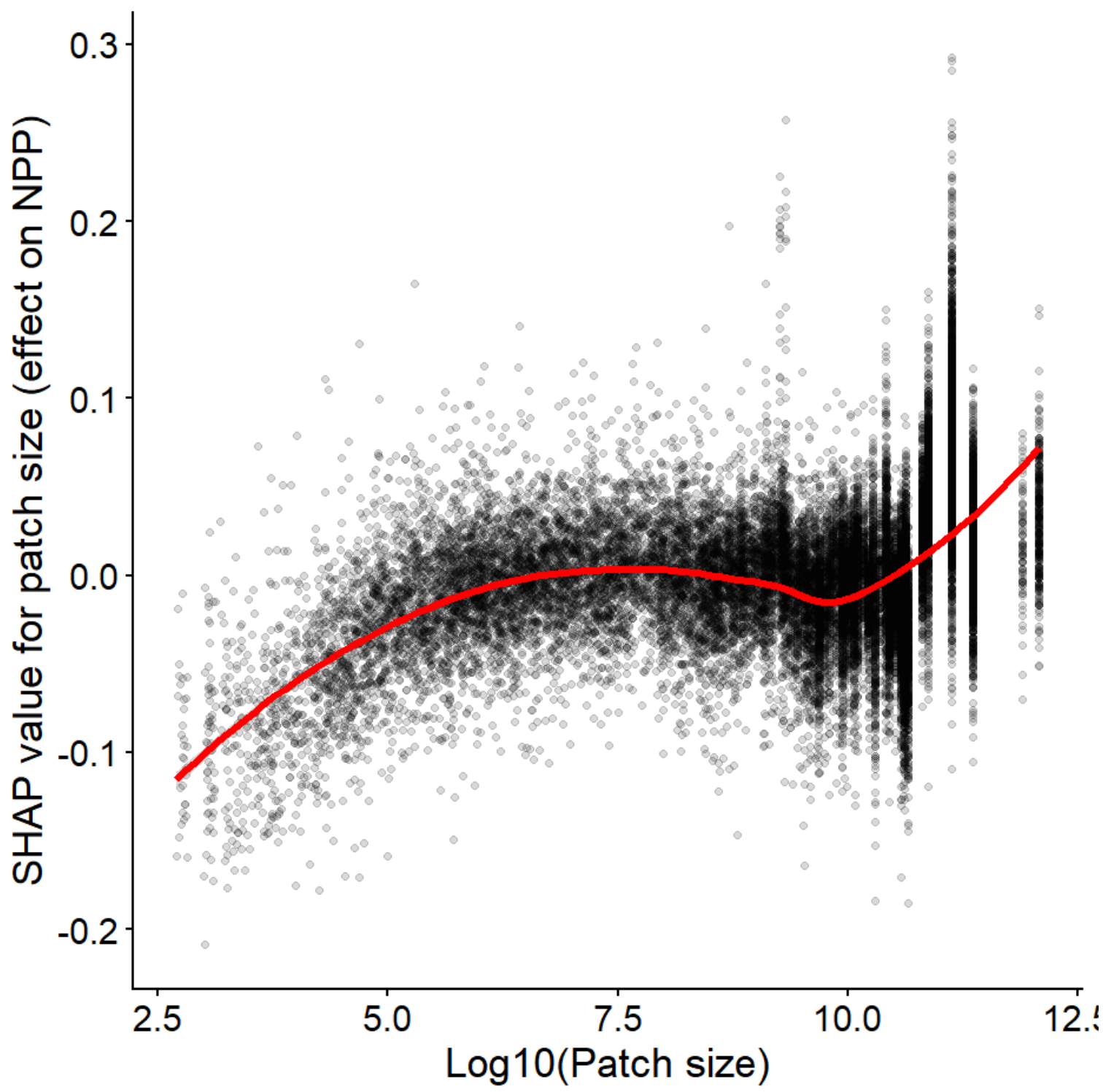

Supplementary Figure 12: SHAP (SHapley Additive exPlanations) values for $\log_{10}$-transformed forest patch size from the random forest model predicting net primary productivity (NPP), while

controlling for climatic, topographic, and soil covariates. Each point represents a forest pixel, with SHAP values indicating the contribution of patch size to the model's NPP prediction relative to the mean prediction. Positive SHAP values indicate higher predicted NPP attributable to larger patch size, whereas negative values indicate lower predicted NPP associated with smaller patches. The red curve shows a smoothed trend, highlighting a nonlinear but overall positive effect of patch size on productivity after accounting for environmental heterogeneity, consistent with superlinear scaling of forest productivity with patch size.

Legend for **Supplementary Table 1**, external .xlsx file. Summaries of NPP, GPP, NDVI and tree cover models, with p-value and beta coefficient for log10-transformed patch size, as well as marginal (not including variation explained by the spatial random effect) R^2 for the model and Moran's I of residuals for each individual biome and each analysis. The full CONUS dataset is too large for computation of Moran's I.

Supplementary Table 2: 6 environmental covariates used in the random forest analysis, with category, source and resolution.

Category	Variable	Source	Res at equator
Climate	Annual Mean Temperature	[5]	900 m
	Annual Precipitation	[5]	900 m
Soil	Soil C:N ratio (0-5 cm, mean)	[6]	250 m
	Soil pH (0-5 cm, mean)	[6]	250 m
Topography	Aspect Sine	[7]	900 m
	Slope	[7]	900 m

1 Owe, M., Jeu, R. d. & Walker, J. A methodology for surface soil moisture and vegetation optical depth retrieval using the microwave polarization difference index. *IEEE Transactions on Geoscience and Remote Sensing* **39**, 1643-1654 (2001). https://doi.org:10.1109/36.942542

2 Hu, D. *et al.* A global daily seamless 9 km vegetation optical depth (VOD) product from 2010 to 2021. *Earth Syst. Sci. Data* **17**, 2849-2872 (2025). https://doi.org:10.5194/essd-17-2849-2025

3 White, E. P., Enquist, B. J. & Green, J. L. ON ESTIMATING THE EXPONENT OF POWER-LAW FREQUENCY DISTRIBUTIONS. *Ecology* **89**, 905-912 (2008). https://doi.org:https://doi.org/10.1890/07-1288.1

4 Robinson, N. P. *et al.* Terrestrial primary production for the conterminous United States derived from Landsat 30 m and MODIS 250 m. *Remote Sensing in Ecology and Conservation* **4**, 264-280 (2018). https://doi.org:https://doi.org/10.1002/rse2.74

5 Karger, D. N. *et al.* Climatologies at high resolution for the earth's land surface areas. *Scientific Data* **4**, 170122 (2017). https://doi.org:10.1038/sdata.2017.122

6 Hengl, T. *et al.* SoilGrids250m: Global gridded soil information based on machine learning. *Plos One* **12**, e0169748 (2017). https://doi.org:10.1371/journal.pone.0169748

7 Amatulli, G. *et al.* A suite of global, cross-scale topographic variables for environmental and biodiversity modeling. *Scientific Data* **5**, 180040 (2018). https://doi.org:10.1038/sdata.2018.40
