# Supplementary material for: Larger forest patches have greater per-area productivity
Source: Nat Ecol Evol. 2026 May 15;10(7):1254–64. doi: 10.1038/s41559-026-03075-5 (PMC13649605; doi:10.1038/s41559-026-03075-5)
Supplement: Supplementary file 2 [file 41559_2026_3075_MOESM2_ESM.pdf]

nature portfolio

Corresponding author(s): Yibiao Zou

Last updated by author(s): Mar 20, 2026

Reporting Summary

Nature Portfolio wishes to improve the reproducibility of the work that we publish. This form provides structure for consistency and transparency in reporting. For further information on Nature Portfolio policies, see our Editorial Policies and the Editorial Policy Checklist.

Statistics

For all statistical analyses, confirm that the following items are present in the figure legend, table legend, main text, or Methods section.

n/a	Confirmed	
☐	☒	The exact sample size (*n*) for each experimental group/condition, given as a discrete number and unit of measurement
☐	☒	A statement on whether measurements were taken from distinct samples or whether the same sample was measured repeatedly
☐	☒	The statistical test(s) used AND whether they are one- or two-sided<br>*Only common tests should be described solely by name; describe more complex techniques in the Methods section.*
☐	☒	A description of all covariates tested
☐	☒	A description of any assumptions or corrections, such as tests of normality and adjustment for multiple comparisons
☐	☒	A full description of the statistical parameters including central tendency (e.g. means) or other basic estimates (e.g. regression coefficient) AND variation (e.g. standard deviation) or associated estimates of uncertainty (e.g. confidence intervals)
☐	☒	For null hypothesis testing, the test statistic (e.g. *F*, *t*, *r*) with confidence intervals, effect sizes, degrees of freedom and *P* value noted<br>*Give P values as exact values whenever suitable.*
☒	☐	For Bayesian analysis, information on the choice of priors and Markov chain Monte Carlo settings
☒	☐	For hierarchical and complex designs, identification of the appropriate level for tests and full reporting of outcomes
☐	☒	Estimates of effect sizes (e.g. Cohen's *d*, Pearson's *r*), indicating how they were calculated

Our web collection on statistics for biologists contains articles on many of the points above.

Software and code

Policy information about availability of computer code

Data collection	Remote sensing data are from different sources. The tree cover products are from Hansen et al., (2013 science). The NPP and GPP data for Continental US are from MODIS produced by Robinson et al. (2018). These are all properly cited in the paper.
Data analysis	The analyses were run in R [R Core Team (2021) R: a language and environment for statistical computing. R Foundation for Statistical Computing, Vienna, Austria. http://www.R-project.org. Accessed 17 Jan 2021] and Google Earth Engine (https://earthengine.google.com). The code used for this study is available at https://github.com/Yibiaozou/PatchSize_Productivity as well as at https://doi.org/10.5281/zenodo.19134763.

For manuscripts utilizing custom algorithms or software that are central to the research but not yet described in published literature, software must be made available to editors and reviewers. We strongly encourage code deposition in a community repository (e.g. GitHub). See the Nature Portfolio guidelines for submitting code & software for further information.

Data

Policy information about availability of data

All manuscripts must include a data availability statement. This statement should provide the following information, where applicable:

- Accession codes, unique identifiers, or web links for publicly available datasets
- A description of any restrictions on data availability
- For clinical datasets or third party data, please ensure that the statement adheres to our policy

The datasets owned by the authors and used in the analyses herein are publicly available via Zenodo at https://doi.org/10.5281/zenodo.19134763. All other datasets are open source cited properly in the paper.

Research involving human participants, their data, or biological material

Policy information about studies with human participants or human data. See also policy information about sex, gender (identity/presentation), and sexual orientation and race, ethnicity and racism.

Reporting on sex and gender	n/a
Reporting on race, ethnicity, or other socially relevant groupings	n/a
Population characteristics	n/a
Recruitment	n/a
Ethics oversight	n/a

Note that full information on the approval of the study protocol must also be provided in the manuscript.

Field-specific reporting

Please select the one below that is the best fit for your research. If you are not sure, read the appropriate sections before making your selection.

☐ Life sciences ☐ Behavioural & social sciences ☒ Ecological, evolutionary & environmental sciences

For a reference copy of the document with all sections, see nature.com/documents/nr-reporting-summary-flat.pdf

Ecological, evolutionary & environmental sciences study design

All studies must disclose on these points even when the disclosure is negative.

Study description	Observational, large-scale geospatial study testing whether forest productivity scales with forest patch size. We quantified patch size and productivity across ~17 million forest patches in CONUS, then extended the analysis globally using coarser-resolution products. Main analyses used linear mixed-effects models; complementary analyses included counterfactual scenarios, random forests, and edge-effect tests.
Research sample	The main sample comprised 16,918,882 forest patches in the conterminous United States derived from the 2010 Hansen 30-m forest cover map (forest defined as ≥30% canopy cover), linked to Landsat-based NPP/GPP data. Additional analyses used ~239,633 randomly sampled forest pixels across CONUS and 2,818 large global forest patches using MODIS NPP/GPP.
Sampling strategy	This was not an experimental study. For the patch-level analyses, all qualifying forest patches were included after applying predefined quality filters. For random forest and edge-effect analyses, forest pixels were randomly sampled across CONUS. Sample size was determined by full available coverage plus computational feasibility for complementary analyses.
Data collection	All data were obtained from existing remote-sensing and derived geospatial products in Google Earth Engine and processed in R. No new field data were collected.
Timing and spatial scale	The study year was 2010. Spatial resolution was 30 m for the CONUS forest-cover and Landsat productivity analyses, 500 m for global MODIS analyses, and 9 km for the VOD robustness test. The main study extent was CONUS, with a global extension for large-patch analyses.
Data exclusions	Patches were excluded if <75% of area had valid productivity coverage or if calculated total productivity was zero. Additional quality filtering removed pixels with excessive gap filling in Landsat NDVI products; global analyses also excluded non-forest-contaminated MODIS pixels.

Reproducibility	All datasets owned by the authors and all analysis code to reproduce the main results are publicly available via Zenodo. Analyses were conducted with scripted workflows in R.
Randomization	No experimental allocation was involved. Randomization applied only to the ~240,000 forest pixels selected for the random forest and edge-effect analyses.
Blinding	Blinding was not relevant because this was an observational analysis of pre-existing geospatial datasets with automated processing and model-based inference.

Did the study involve field work? ☐ Yes ☒ No

Reporting for specific materials, systems and methods

We require information from authors about some types of materials, experimental systems and methods used in many studies. Here, indicate whether each material, system or method listed is relevant to your study. If you are not sure if a list item applies to your research, read the appropriate section before selecting a response.

Materials & experimental systems

n/a	Involved in the study
☒	☐ Antibodies
☒	☐ Eukaryotic cell lines
☒	☐ Palaeontology and archaeology
☒	☐ Animals and other organisms
☒	☐ Clinical data
☒	☐ Dual use research of concern
☒	☐ Plants

Methods

n/a	Involved in the study
☒	☐ ChIP-seq
☒	☐ Flow cytometry
☒	☐ MRI-based neuroimaging

Plants

Seed stocks	n/a
Novel plant genotypes	n/a
Authentication	n/a
