# Supplementary material for: Larger forest patches have greater per-area productivity
Source: Nat Ecol Evol. 2026 May 15;10(7):1254–64. doi: 10.1038/s41559-026-03075-5 (PMC13649605; doi:10.1038/s41559-026-03075-5)
Supplement: Supplementary file 3 [file 41559_2026_3075_MOESM3_ESM.pdf]

nature portfolio

Peer Review File

Larger forest patches have greater per-area productivity

Corresponding Author: Mr Yibiao Zou

Version 0:

Decision Letter:

3rd December 2024

*Please ensure you delete the link to your author homepage in this e-mail if you wish to forward it to your co-authors.

Dear Mr Zou,

Thank you for your patience while your Article entitled "Superlinear scaling of productivity with patch size across American forests" was under review. We have now received three referee reports, which are provided below. As you will see, the reviewers find your work of potential interest but have raised quite substantial concerns that must be addressed. In light of these comments, we cannot accept the manuscript for publication but would be very interested in considering a revised version that addresses the reviewers' criticisms.

We hope that you will find the reviewers' comments useful as you decide how to proceed. We are committed to providing a fair and constructive peer-review process. Please do not hesitate to contact us if there are specific requests from the reviewers that you believe are technically impossible or unlikely to yield a meaningful outcome. This said, please bear in mind that we will be reluctant to approach the referees again in the absence of major revisions, including additional analyses based on other productivity data as recommended by Reviewer 2, and it will be important to address Reviewer 3's concerns on the counterfactual models.

Regarding Reviewer 3's comments on the use of the term "[forest] patch" in this study, please consider whether it should be replaced by something more appropriate in the text. For example, we wonder whether the title should be changed to "Superlinear scaling of productivity with forest size across the United States" or similar. Perhaps this could also help you to clarify the novelty of your work to the reviewers.

If revising your manuscript:

* Please highlight all changes in the manuscript text file in Microsoft Word format.

* Include a “Response to reviewers” document detailing, point-by-point, how you addressed each referee comment. If no action was taken to address a point, you must provide a compelling argument. This response will be sent back to the referees along with the revised manuscript.

* If you have not done so already we suggest that you begin to revise your manuscript so that it conforms to our Article format instructions at http://www.nature.com/natecolevol/info/final-submission. Refer also to any guidelines provided in this letter.

* Include a revised version of any required reporting checklist. It will be available to referees (and, potentially, statisticians) to aid in their evaluation if the manuscript goes back for peer review. A revised checklist is essential for re-review of the paper.

Please use the link below to submit a revised paper:

Link Redacted

Note: This URL links to your confidential home page and associated information about manuscripts you may have submitted, or that you are reviewing for us. If you wish to forward this email to co-authors, please delete the link to your homepage.

If you wish to submit a suitably revised manuscript we would hope to receive it within 6 months. If you cannot send it within this time, please let us know. We will be happy to consider your revision so long as nothing similar has been accepted for publication at Nature Ecology & Evolution or published elsewhere.

Nature Ecology & Evolution is committed to improving transparency in authorship. As part of our efforts in this direction, we are now requesting that all authors identified as 'corresponding author' on published papers create and link their Open Researcher and Contributor Identifier (ORCID) with their account on the Manuscript Tracking System (MTS), prior to acceptance. This applies to primary research papers only. ORCID helps the scientific community achieve unambiguous attribution of all scholarly contributions. You can create and link your ORCID from the home page of the MTS by clicking on 'Modify my Springer Nature account'. For more information please visit please visit www.springernature.com/orcid.

Please do not hesitate to contact me if you have any questions or would like to discuss the required revisions further.

Thank you for the opportunity to review your work.

[redacted]

Reviewers' comments:

Reviewer #1 (Remarks to the Author):

The paper examines the impact of forest fragmentation on carbon sequestration, highlighting that large contiguous forest patches exhibit higher mean canopy cover, vegetation reflectance, and annual net primary productivity per unit area compared to smaller, isolated patches. The study emphasized the potential risk associated with "net zero deforestation" policies, and the overlooked carbon loss from fragmentation.
The observed evidence, statistical approach, and validation process appear robust and convincing. These elements collectively strengthen the reliability of the study's findings and its overall scientific rigor. I recommend a major revision, as several weaknesses need to be addressed before the manuscript is ready for publication.

Major comments:
The underlying mechanism behind this superlinear scaling remains unclear. While a systematic quantification of the contributions of different drivers may be beyond the scope of this study, it would be beneficial to clearly outline the potential mechanisms and pathways that could explain this phenomenon.
The rationale for selecting the conterminous United States (CONUS) as the study area to address this topic should be elaborated. Is the forest in CONUS considered a significant carbon sink globally, and how does it compare to other regions? The choice of CONUS as the study area may not be fully justified if it is solely based on the availability of data, such as 30-m GPP and NPP. Clarifying why CONUS is particularly suitable for addressing this topic beyond data availability would strengthen the study's rationale.
Additionally, it would be helpful to clarify whether the findings are specific to CONUS or if they could be generalized to forests in other parts of the world, particularly those with different ecological or climatic conditions.
Please provide more details about the spatial random effect approach, specifically how they control for large-scale socio-environmental variation, why linear mixed-effects models were chosen, and how they isolate patch size effects from other variables, which is vital for this study.

Minor comments:
L15–17: This sentence is somewhat difficult to follow, particularly the phrase "beyond those encompassed by loss of forested area." Consider rephrasing for clarity, perhaps by explicitly stating the additional consequences of fragmentation beyond the direct removal of forests and their carbon storage.
L24–25: The phrase "this superlinear scaling relationship contributes nearly one-quarter of total annual forest productivity" is unclear. It's difficult to interpret how a relationship "contributes" to productivity. Consider rephrasing to clarify whether this means that large patches account for 25% of productivity or if fragmentation reduces productivity by this amount.
L37: I recommend avoiding the abbreviation of "carbon" as "C" here and throughout the text.
L51: Instead of "the scale of the resulting carbon cycle impacts," consider specifying "the scale of the resulting carbon sequestration impacts" to make the statement more precise and aligned with the context of the study.
L75: The term "Features" is unclear in this context.
L114: Why was the grassland biome excluded from the analysis? Clarifying the rationale.
Line 147: The phrase "a plausible causal element" is ambiguous. Could you clarify what is meant by this?
L180: The statement "Interior forest differs abiotically from edge area, which tends to be hotter and dryer" is somewhat confusing. Does this mean that edge forests are hotter and drier? If so, could you provide more explanation on why this occurs?
L180-196: Please elaborate on why some forests exhibit positive edge effects, while others show negative effects.
Lines 197–198: The statement "large forest patches should have a greater diversity of species than small ones" requires clarification. Are you referring to forest plant species or animal species (related to the habitat)? If it is the latter, it would be helpful to explain how a greater diversity of animal species contributes to increased vegetation NPP. For example, does it involve ecological interactions like pollination, seed dispersal, or herbivory regulation? Providing more context would make this argument clearer and more robust.
Line 212: The term "potential drivers" is vague. Could you specify what these drivers refer to?

L273: Please elaborate on linear mixed effects models, and why they can control for large-scale scio-environmental variation.
L301: Specify what is “C cycle consequences”. Is it the carbon consequence or NPP?

Reviewer #1 (Remarks on code availability):

The author has shared their GitHub repository, but more information is needed to fully understand the connection between the code and the manuscript.

Reviewer #2 (Remarks to the Author):

The manuscript entitled "Superlinear scaling of productivity with patch size across American forests " attempt to demonstrate a greater productivity per area (m2) associated with increases in tree patch sizes. They used the biome-specific grouped NPP/GPP/NDVI/tree cover as a function of patch size to show a positive relationship between productivity per area unit and patch size, terming superlinear scaling of productivity. However, the positive relationships except for cover in Fig. 2h almost disappeared when looking into the relationships established at the level of individual patches (Fig. 2e-g), which actually can better reflect the real pattern. This makes the key result shaky. Moreover, the finding of the lower productivity per area in smaller forest patches compared to large patches is well known and linked to “edge effects” of forest fragmentation (see relevant refs below). In addition, I have important concerns regarding the dependence of productivity (NPP/GPP/NDVI/tree cover calculated based on Landsat reflectance bands) on patch size quantified by Landsat bands as well in the linear mixed model, while the authors have mentioned this issue (line282-287). I suggest using independent productivity data sources (e.g., VOD).

It is not clear how to control for superlinear scaling in the mixed models.
I struggled to understand the relationship established between the power law parameter of α and productivity, does a higher α indicate a faster decrease in the number of large patches? and thus accompanied with a greater loss of NPP? It is not clear what this means in the current format.

Haddad, N. M. et al. Habitat fragmentation and its lasting impact on Earth’s ecosystems. Sci. Adv. 1, 1–10 (2015).
Chaplin-Kramer, R. et al. Degradation in carbon stocks near tropical forest edges. Nat. Commun. 6, 1–6 (2015).
Brinck, K. et al. High resolution analysis of tropical forest fragmentation and its impact on the global carbon cycle. Nat. Commun. 8, (2017).
Fischer, R. et al. Accelerated forest fragmentation leads to critical increase in tropical forest edge area. Sci. Adv. 1, (2021).

Specific comments:
L94 The units for GPP/NPP should be complete.
L259 remove raster
L266 please add subheadings to the section of Analytical workflow, which is now very confusing to read.
L287 specify Type II
L288 but the explanatory power (R2) can determine how importance of each predictor (e.g., patch size) contributes to the predicted productivity.
L385 add quantification information for the fitting lines on the right panel, m-2 to m-2.
L397 the quantification information is missing in figure 3b, it should be clearly clarified how the models run with and without the superlinear scaling being controlled in the method (line 301-308?). It is now difficult to understand this point or the design.
L402 B) to b)
L412 please elaborate on the window size of tree patches on the left panel and add b) on the right.

Reviewer #3 (Remarks to the Author):

The current paper, “Superlinear scaling of productivity with patch size across American forests", studies the impact of forest patch size on productivity and, thus, the influence of forest fragmentation. The authors have invested an enormous effort to analyse 17 million forest patches (a forest patch means here a forest, not a gap or disturbed area in a forest) in the conterminous United States. While the study offers many interesting insights, the authors could better point out its novelty compared to existing studies, consider alternative explanations (as the mere forest size might not be the primary driver of productivity) and reconsider their counterfactual modelling approach.
As the reviewer’s expert opinion on the counterfactual modelling component was asked for, I will start with this aspect. Counterfactual models can be empirical (1) or optimisation (2)- or agent-based (3). Counterfactual models are widespread in providing causal inference in land system science (4). However, caution is warranted when using empirical approaches, as here, the counterfactuals might not be independent of the observed empirical data (5,6), so the effect to be analysed may still be blurred. This aspect appears to apply to the current study. The authors replaced the original forest patch size with the mean patch size in log10 space for the corresponding biome to remove the impact of the patch size on productivity. However, as one may expect that the average patch sizes will vary across biomes with large average gap sizes representing, on average, also large forest patches, the empirical patch size and productivity relation does still influence the results. Thus, the effect to be analysed may not have been eliminated from the intended counterfactual model.

I want to add another modelling aspect here: The study uses a 1-degree by 1-degree grid (which covers well > 1 million hectares) to account for the compound impact of a bundle of variables not explicitly addressed by the model (using a random effect model). Aside from climate, this random effect shall account for differences in the socio-economic context, including the impacts of forest management and land-use change dynamics. However, considering such large grid cells are less suitable to account for differences in often small managed (inherently low density) or leftover forests (remnants which are necessarily heavily degraded) and extensive undisturbed contiguous natural forests (natural forests dominate the USA forest cover by size).

This brings me to suggest that one might also use an alternative explanation for the results found. The study might also show the impact of the forest type and quality and the influence of the (human) disturbance or management on the canopy cover (much lower in smaller-sized forests) on productivity, but less the impact of forest size. One may expect that extensive contiguous forests are commonly largely undisturbed and thus more productive.

The novelty aspect could also be justified better, given that previous publications have found similar results, e.g. ref. (7,8). In addition, one could perhaps better point out that a patch is a still-standing forest and not a forest gap caused by disturbances; see ref. (9) for using "patch" to refer to a disturbed forest area.

References

1. Groom, B., Palmer, C. & Sileci, L. Carbon emissions reductions from Indonesia's moratorium on forest concessions are cost-effective yet contribute little to Paris pledges. PNAS 119; 10.1073/pnas.2102613119 (2022).
2. Babatunde, K. A., Begum, R. A. & Said, F. F. Application of computable general equilibrium (CGE) to climate change mitigation policy: A systematic review. Renewable and Sustainable Energy Reviews 78, 61–71; 10.1016/j.rser.2017.04.064 (2017).
3. Knoke, T. et al. Trends in tropical forest loss and the social value of emission reductions. Nat Sustain 6, 1373–1384; 10.1038/s41893-023-01175-9 (2023).
4. Magliocca, N. R., Dhungana, P. & Sink, C. D. Review of counterfactual land change modeling for causal inference in land system science. J. Land Use Sci. 18, 1–24; 10.1080/1747423X.2023.2173325 (2023).
5. Hoover, K. D. Econometrics as observation: the Lucas critique and the nature of econometric inference. Journal of Economic Methodology 1, 65–80; 10.1080/13501789400000006 (1994).
6. Moos, K. A. The Facts and the Values of the Lucas Critique. Review of Political Economy 31, 1–25; 10.1080/09538259.2019.1586363 (2019).
7. Ma, L., Shen, C., Lou, D., Fu, S. & Guan, D. Ecosystem carbon storage in forest fragments of differing patch size. Sci Rep 7, 13173; 10.1038/s41598-017-13598-4 (2017).
8. Islam, M., Deb, G. P. & Rahman, M. Forest fragmentation reduced carbon storage in a moist tropical forest in Bangladesh: Implications for policy development. Land Use Policy 65, 15–25; 10.1016/j.landusepol.2017.03.025 (2017).
9. Maroschek, M., Seidl, R., Poschlod, B. & Senf, C. Quantifying patch size distributions of forest disturbances in protected areas across the European Alps. Journal of Biogeography 51, 368–381; 10.1111/jbi.14760 (2024).

Version 1:

Decision Letter:

21st November 2025

*Please ensure you delete the link to your author homepage in this e-mail if you wish to forward it to your co-authors.

Dear Yibiao,

Your manuscript entitled "Superlinear scaling of productivity with forest patch size" has now been seen by two of the original reviewers (one of whom also assessed your response to the missing reviewer). As you will see from the reports below, the reviewers find the manuscript improved in revision, but some concerns remain, particularly about the conterfactual models and the strength of the conclusions to be derived from them. We therefore invite you to revise your manuscript taking into account all the reviewers' comments.

We are committed to providing a fair and constructive peer-review process. Do not hesitate to contact us if there are specific requests from the reviewers that you believe are technically impossible or unlikely to yield a meaningful outcome. However, I must note that Reviewer #3's concerns on the current counterfactual approach (or at least its fit to the aims of the study) are important and we ask that you consider the alternative modelling approach outlined in their report.

When revising your manuscript:

* Please provide the manuscript in Microsoft Word format and highlight all changes in the same file or a copy.

* Include a "Response to reviewers" document detailing, point-by-point, how you addressed each reviewer comment. If no action was taken to address a point, you must provide a compelling argument. This response will be sent back to the reviewers along with the revised manuscript.

* If you have not done so already please begin to revise your manuscript so that it conforms to our Article format instructions at http://www.nature.com/natecolevol/info/final-submission. Refer also to any guidelines provided in this letter.

* Include a revised version of any required reporting checklist. It will be available to referees (and, potentially, statisticians) to

aid in their evaluation if the manuscript goes back for peer review. A revised checklist is essential for re-review of the paper.

* Extended Data Figures - please ensure that any supplementary figures and tables that are crucial to the manuscript’s conclusions are converted into Extended Data figures and tables to increase visibility of these data. Extended Data figures and tables are online-only (present in the online PDF and full-text HTML versions of the paper), peer-reviewed display items that provide essential background to the article but are not included in the main article due to space constraints. A maximum of ten Extended Data display items (figures and tables) is permitted.

We hope to receive your revised manuscript within three months. If you cannot send it within this time, please let us know. We will be happy to consider your revision so long as nothing similar has been accepted for publication at Nature Ecology & Evolution or published elsewhere. Please use the link below to submit your revised manuscript and related files:

Link Redacted

Note: This URL links to your confidential home page and associated information about manuscripts you may have submitted, or that you are reviewing for us. If you wish to forward this email to co-authors, please delete the link to your homepage.

Nature Ecology & Evolution is committed to improving transparency in authorship. As part of our efforts in this direction, we are now requesting that all authors identified as ‘corresponding author’ on published papers create and link their Open Researcher and Contributor Identifier (ORCID) with their account on the Manuscript Tracking System (MTS), prior to acceptance. ORCID helps the scientific community achieve unambiguous attribution of all scholarly contributions. You can create and link your ORCID from the home page of the MTS by clicking on ‘Modify my Springer Nature account’. For more information please visit please visit www.springernature.com/orcid.

We look forward to seeing the revised manuscript.

[redacted]

****

Reviewers' comments:

Reviewer #2 (Remarks to the Author):

Thank you for the substantial revisions. The authors have basically addressed my concerns in the revised manuscript. I believe that the result is robust with regard to the positive relationships between NPP/GPP and forest patch sizes, although this relationship is much clearer at the biome-level than at the patch level.
Regarding potential mechanisms, this table 2 added to the understanding of the empirical relationship NPP-patch, where respiration might be worth mentioning due to the lack of mechanism analysis. The patch size is an indirect factor that affects carbon uptake by forcing these direct processes such as changes in hydrological activities/nutrients, microclimate conditions that affect carbon cycles. The edge effect is also an indirect factor. A pathway depicting these direct and indirect relationships between these variables would be thus helpful. Moreover, it would be better to refine the highlighted points, as the issue of fragmentation-induced carbon loss has been overused in existing literatures. btw, the x-axis titles are missing in the figure 2 and the similar ones.

**Comments on the responses to Reviewer #1

The authors properly addressed the major weaknesses raised. They summarised several aspects of the potential mechanisms underlying the superliner scaling and clarified the rationale behind using CONUS to exploit the relationships between carbon sinks and patch sizes. They further strengthened the superliner scaling relationships by extending the forest extents from CONUS to a global scale. This is a worthwhile test. Here, additional analysis is suggested, including quantitative information that explains the superliner scaling effects or shows the contribution that superliner scaling makes to future studies. For example, a potential test could be conducted to see if a model including the superliner effect could improve the prediction of forest carbon sequestration. This is because, at this stage, the section on mechanism is highly narrative-based, making it seem very trivial compared to the primary documentation of the superliner relationship. I further suggest including these relevant maps/figures (such as global forest cover, patch distributions and biome divisions, etc) in addition to the extended figs.5-10 in the supplementary documents to make it easier to interpret these results visually.

Reviewer #3 (Remarks to the Author):

The revision of the paper, “Superlinear scaling of productivity with patch size across American forests", addressed many of my comments nicely, while the consistency of the conclusions and the choice of the counterfactual still do not convince the reviewer fully.
The choice of a counterfactual is always subjective and depends on what one would like to investigate. Here, the authors

use a $\log_{10}$(patch size) coefficient to create a counterfactual and interpret this as an average within-cell causal effect of increasing patch size on per-area productivity. The aim is to quantify the extra carbon uptake attributable to the superlinear size-productivity relationship. This is scientifically interesting and, of course, not wrong. However, by using the average effect of increasing patch size, this counterfactual does not remove the effect of the patch size on the productivity fully, as the cumulative NPP is still the one affected by patch size. Consequently, the counterfactual does not solely refer to NPP values without any fragmentation. A counterfactual to support the policy statements of the study, i.e. that the NPP loss incurred by fragmentation shall be considered in future forest protection policies, requires a counterfactual without any impact of fragmentation.
The simplest such counterfactual would be the maximum NPP of 0.8 kg m-2 for large and unfragmented forests. Compared to this counterfactual, the highest fragmentation and the smallest patches are associated with an NPP of 0.58 kg m-2 (Tab. 1), which we shall interpret as a reduction of NPP by 27.5% (1-0.58/0.8). This number already constitutes a strong difference compared with the reported increase of NPP by 37.83% by comparing maximum with minimum productivity. However, the demonstrated consideration assumes that all forests would be maximally fragmented, while very small forest patches cover only around 1% of the total analysed area (Fig. 1b). When taking the area covered by the different patch sizes (blue line in Fig. 1b), one may roughly find a total reduction of NPP by 13% caused by fragmentation effects. However, my consideration is not exact, as I read the numbers needed from Figures 1b and 2a. Nevertheless, I would consider this type of effect interesting to inform policy concerning the impact of fragmentation, which is quite different compared to the effect of 32% as resulting from the author's counterfactual. It would be more suitable to support the policy statements of the study.
The main policy paper cited as a justification for the study's policy statements is Lyons-White et al. (2020), where I struggle to find the direct connection to forest fragmentation issues. It could be made more explicit how Lyons-White's study applies to the study under consideration.
Finally, the authors find some negative relationships between forest patch size and productivity, for example, in the South American dry forest. How can those be explained?
References
Lyons-White J, Pollard EH, Catalano AS, Knight AT (2020) Rethinking zero deforestation beyond 2020 to more equitably and effectively conserve tropical forests. One Earth 3:714–726. https://doi.org/10.1016/j.oneear.2020.11.007

Version 2:

Decision Letter:

11th March 2026

Dear Yibiao,

Thank you for submitting your revised manuscript "Larger forest patches exhibit greater per-area productivity in the U.S. and worldwide" (NATECOLEVOL-24103030B). In light of Reviewer 3's comments and our careful examination of the response to Reviewer 1's previous comments, we find that the paper has sufficiently improved in revision. Therefore, we will be happy in principle to publish it in Nature Ecology & Evolution, pending minor revisions and compliance with our editorial and formatting guidelines.

We are now performing detailed checks on your paper and will send you a checklist detailing our editorial and formatting requirements in about a week. Please do not upload the final materials and make any revisions until you receive this additional information from us.

Thank you again for your interest in Nature Ecology & Evolution. Please do not hesitate to contact me if you have any questions.

[redacted]

Reviewer #3 (Remarks to the Author):

Dear authors,

thank you for integrating all my remaining comments. I have no further suggestions and recomend acceptance of the informative study.

The reviewer

Open Access This Peer Review File is licensed under a Creative Commons Attribution 4.0 International License, which permits use, sharing, adaptation, distribution and reproduction in any medium or format, as long as you give appropriate credit to the original author(s) and the source, provide a link to the Creative Commons license, and indicate if changes were made.
In cases where reviewers are anonymous, credit should be given to 'Anonymous Referee' and the source.
The images or other third party material in this Peer Review File are included in the article's Creative Commons license, unless indicated otherwise in a credit line to the material. If material is not included in the article's Creative Commons license and your intended use is not permitted by statutory regulation or exceeds the permitted use, you will need to obtain permission directly from the copyright holder.
To view a copy of this license, visit https://creativecommons.org/licenses/by/4.0/

RESPONSE TO REVIEWER 1

[Comment 1]
The paper examines the impact of forest fragmentation on carbon sequestration, highlighting that large contiguous forest patches exhibit higher mean canopy cover, vegetation reflectance, and annual net primary productivity per unit area compared to smaller, isolated patches. The study emphasized the potential risk associated with "net zero deforestation" policies, and the overlooked carbon loss from fragmentation.
The observed evidence, statistical approach, and validation process appear robust and convincing. These elements collectively strengthen the reliability of the study's findings and its overall scientific rigor. I recommend a major revision, as several weaknesses need to be addressed before the manuscript is ready for publication.
Response 1: We thank the reviewer for the positive and constructive feedback on our paper. We also very much appreciate the concerns raised by the reviewer and believe we were able to address all of them as outlined below.

[Comment 2]
The underlying mechanism behind this superlinear scaling remains unclear. While a systematic quantification of the contributions of different drivers may be beyond the scope of this study, it would be beneficial to clearly outline the potential mechanisms and pathways that could explain this phenomenon.
Response 2: We have added discussion of two major potential mechanisms and pathways (i.e., edge effects and biodiversity) in lines 219-266. We have also added one table (Table 2) to outline four potential mechanisms and pathways that could generate this pattern.

[Comment 3]
The rationale for selecting the conterminous United States (CONUS) as the study area to address this topic should be elaborated. Is the forest in CONUS considered a significant carbon sink globally, and how does it compare to other regions? The choice of CONUS as the study area may not be fully justified if it is solely based on the availability of data, such as 30-m GPP and NPP. Clarifying why CONUS is particularly suitable for addressing this topic beyond data availability would strengthen the study's rationale.
Response 3: We fully agree with the reviewer's perspective that our choice of study region requires further justification. We have provided the extra justification in lines 277-284:" Beyond the availability of high-resolution productivity data, we selected the CONUS as the main study region for this work because its forests represent a significant portion of global temperate forest carbon sequestration, contributing substantially to global carbon sinks and climate regulation[35-37]. Furthermore, the diversity of forest types across CONUS provides a broad ecological context to assess the relationship between patch size and productivity, making findings widely generalizable[38,39]. Given its history of extensive land-use changes, forest fragmentation, and ongoing restoration efforts, CONUS offers unique insights into how landscape structure affects productivity, with direct implications for global forest management policies[40,41]".

[Comment 4]
Additionally, it would be helpful to clarify whether the findings are specific to CONUS or if they could be generalized to forests in other parts of the world, particularly those with different ecological or climatic conditions.
Response 4: To assess generality beyond CONUS, we extended the analysis to global forests, focusing on large patches to harmonize pixel–patch scale across products (the coarsest resolution of satellite product in use is 9km). Applying the same mixed-effects framework to NPP, GPP, and VOD (vegetation optical depth, a passive-microwave proxy for canopy water/biomass), we observed positive size–productivity relationships across major forest biomes worldwide, suggesting that the scaling pattern is a general property of forest

landscapes at broad spatial scales (Extended Data Figs. 3–10). We have included these analysis and results in the main text lines 187-196, 407-428.

[Comment 5]
Please provide more details about the spatial random effect approach, specifically how they control for large-scale socio-environmental variation, why linear mixed-effects models were chosen, and how they isolate patch size effects from other variables, which is vital for this study.
Response 5: We have provided more clarification for the spatial random effect approach in lines 302-321.

[Comment 6]
L15–17: This sentence is somewhat difficult to follow, particularly the phrase "beyond those encompassed by loss of forested area." Consider rephrasing for clarity, perhaps by explicitly stating the additional consequences of fragmentation beyond the direct removal of forests and their carbon storage.
Response 6: We have rephrased this sentence as “If smaller patches sequester less carbon per hectare than larger ones, fragmentation can depress carbon uptake beyond losses due to reduced area, yet most policies still prioritize hectares over configuration” in lines 14-16.

[Comment 7]
L24–25: The phrase "this superlinear scaling relationship contributes nearly one-quarter of total annual forest productivity" is unclear. It’s difficult to interpret how a relationship "contributes" to productivity. Consider rephrasing to clarify whether this means that large patches account for 25% of productivity or if fragmentation reduces productivity by this amount.
Response 7: We have rephrased this sentence as “A hectare embedded in a 100,000 km² forest is, on average, 38% more productive than an isolated hectare under comparable conditions, and the productivity boost associated with large patches accounts for nearly one-quarter of total annual forest productivity across CONUS” in lines 19-22.

[Comment 8]
L37: I recommend avoiding the abbreviation of "carbon" as "C" here and throughout the text.
Response 8: We have changed all “C” back to “carbon” as suggested.

[Comment 9]
L51: Instead of "the scale of the resulting carbon cycle impacts," consider specifying "the scale of the resulting carbon sequestration impacts" to make the statement more precise and aligned with the context of the study.

Response 9: We have rephrased the sentence in lines 51-53 “Despite evidence of reduced carbon stocks in smaller fragments at local and regional scales, the consequences of fragmentation for productivity (carbon sequestration rates) over short and long time horizons are still poorly understood”.

[Comment 10]
L75: The term "Features" is unclear in this context.

Response 10: We have removed this sentence because it provided information already conveyed in the preceding sentence.

[Comment 11]
L114: Why was the grassland biome excluded from the analysis? Clarifying the rationale.
Line 147: The phrase "a plausible causal element" is ambiguous. Could you clarify what is meant by this?

Response 11: The grassland biomes were initially excluded from the analysis mainly because they are not forest dominated biomes. Given there are still forest patches in these biomes, we now include them back in the main text analysis. And the positive patch size - productivity relationship still holds in these grassland biomes (Fig. 2-3).

Here "a plausible causal element" mean a causal interpretation under stated assumptions, not proof of causation. Specifically, if (i) unobserved confounding is negligible after conditioning on the 1° grid-cell random effect and observed covariates (Methods), (ii) the model is adequately specified, and (iii) interference across patches is limited, then the coefficient on $\log_{10}$(patch size) can be read as the average within-cell causal effect of increasing patch size on per-area productivity. This interpretation is consistent with (a) experimental and quasi-experimental studies showing that exogenous edge creation/fragmentation lowers growth and biomass[11,12], (b) our complementary specification that adjusts for high-resolution climate, soil, topography, management, forest age, and access, and (c) robustness to an independent productivity proxy (VOD) and to a global large-patch analysis (Extended Data Fig. 1-10). We have rephrased the text in lines 142-158 accordingly.

[Comment 12]
L180: The statement "Interior forest differs abiotically from edge area, which tends to be hotter and dryer" is somewhat confusing. Does this mean that edge forests are hotter and drier? If so, could you provide more explanation on why this occurs?

Response 12: We agree that the original sentence could be more explicit. We have now provide clarification in lines 200-203 as "Edge forests are typically hotter and drier than interior forests due to greater exposure to sunlight, wind, and temperature fluctuations[1]. In contrast, forest interiors are buffered by surrounding vegetation, which helps maintain more stable and humid microclimatic conditions[23]".

[Comment 13]
L180-196: Please elaborate on why some forests exhibit positive edge effects, while others show negative effects.

Response 13: We have rephrased the whole paragraph in lines 199-217 to further clarify the reasons for positive and negative edge effects.

[Comment 14]
Lines 197–198: The statement "large forest patches should have a greater diversity of species than small ones" requires clarification. Are you referring to forest plant species or animal species (related to the habitat)? If it is the latter, it would be helpful to explain how a greater diversity of animal species contributes to increased vegetation NPP. For example, does it involve ecological interactions like pollination, seed dispersal, or herbivory regulation? Providing more context would make this argument clearer and more robust.
Response 14: We have provided more context on how plant and animal diversity can promote productivity in lines 218-230.

[Comment 15]
Line 212: The term "potential drivers" is vague. Could you specify what these drivers refer to?

Response 15: We have rephrased this sentence in lines 236-238 as "Further studies are therefore needed to quantify how different drivers—such as edge effects and biodiversity—contribute to these patterns, recognizing that their relative importance may differ across biomes and spatial scales".

[Comment 16]
L273: Please elaborate on linear mixed effects models, and why they can control for large-scale scio-environmental variation.

Response 16: We have provided more clarification on linear mixed effects models in lines 302-321.

[Comment 17]
L301: Specify what is “C cycle consequences”. Is it the carbon consequence or NPP?

Response 17: We have rephrased the term as “carbon sequestration consequences” throughout the paper.

[Comment 18]
The author has shared their GitHub repository, but more information is needed to fully understand the connection between the code and the manuscript.

Response 18: We have provided the code to reproduce the main results in the manuscript in the GitHub Repository: “https://github.com/Yibiaozou/PatchSize_Productivity”.

RESPONSE TO REVIEWER 2

[Comment 1]
The manuscript entitled "Superlinear scaling of productivity with patch size across American forests " attempt to demonstrate a greater productivity per area (m2) associated with increases in tree patch sizes. They used the biome-specific grouped NPP/GPP/NDVI/tree cover as a function of patch size to show a positive relationship between productivity per area unit and patch size, terming superlinear scaling of productivity. **However, the positive relationships except for cover in Fig. 2h almost disappeared when looking into the relationships established at the level of individual patches (Fig. 2e-g), which actually can better reflect the real pattern. This makes the key result shaky.** Moreover, the finding of the lower productivity per area in smaller forest patches compared to large patches is well known and linked to “edge effects” of forest fragmentation (see relevant refs below). In addition, I have important concerns regarding the dependence of productivity (NPP/GPP/NDVI/tree cover calculated based on Landsat reflectance bands) on patch size quantified by Landsat bands as well in the linear mixed model, while the authors have mentioned this issue (line282-287). I suggest using independent productivity data sources (e.g., VOD).
Response 19: Thank you for the thoughtful comments. The weak visual trend in Fig. 2d–f reflects raw, unconditioned scatter across millions of patches with strong between-location heterogeneity, and over-plotting; our inference instead comes from within–grid-cell linear mixed-effects models that hold broad spatial context constant via a 1° cell random intercept. In these models, the $\log_{10}$(size) effect is consistently positive across CONUS and most biomes, with minimal residual spatial autocorrelation, and it remains positive in a stricter spatially explicit covariate specification that includes high-resolution climate, soil, topography, management, forest age, and access (Extended Data Fig. 2). We agree that edge effects can reduce productivity in small fragments (highlighted as a key mechanism in Table 2); our contribution is to quantify the continent-wide per-area size effect, demonstrate superlinear scaling of total NPP with size, and link it to the Pareto α configuration parameter to show how fragmentation alone (holding area fixed) alters total NPP. To address potential dependence on Landsat reflectance, we repeated the analysis with Vegetation Optical Depth (VOD)—independent of optical reflectance—and found the same pattern in CONUS ($R^2 \approx 0.19$) and across global large patches using NPP, GPP, and VOD. Notably, the strength of the size–NPP relationship varied across continents, with higher explanatory power in tropical-dominated regions—such as South America, Africa, Asia, and Oceania ($R^2 \approx 0.10$–

0.44)—compared to temperate regions like Europe and North America ($R^2 \approx 0.007–0.031$). We revised the manuscript (lines 171-196, 302–321, 358-428) to (i) clarify that model-based within-cell effects underpin inference, and (ii) include a consolidated "Addressing potential limitations" subsection reporting the covariate, VOD, and global robustness analyses.

[Comment 2]
It is not clear how to control for superlinear scaling in the mixed models.
I struggled to understand the relationship established between the power law parameter of α and productivity, does a higher α indicate a faster decrease in the number of large patches? and thus accompanied with a greater loss of NPP? It is not clear what this means in the current format.
Response 20: See Response 5, we have provided more clarification for the spatial random effect approach in lines 302-321.
Thank you for raising this—our original wording was too terse. In our paper, α is the Pareto shape parameter fitted to the empirical patch-size distribution, defined via the complementary CDF. Thus, a higher α means the upper tail decays faster, i.e., fewer very large patches and relatively more small ones—a more fragmented configuration. We then combine this size distribution with our estimated within-cell size–productivity relationship (per-area productivity increases with $\log_{10}$(size)) to compute total NPP while holding total forest area fixed. Intuitively, when α increases, the distribution shifts mass from large to small patches; because per-area productivity rises with size, the area-weighted mean productivity falls, so total NPP decreases even if total area is unchanged. Conversely, lowering α (more large patches) raises total NPP. To make this clearer, we have (i) defined α precisely at first mention in lines 152-158. (ii) add one sentence in the Figure 4 caption: "On a log–log plot, the slope of the patch-size CCDF equals −α; larger α indicates a steeper decline in large patches and, given a positive size–productivity slope, a lower total NPP at fixed area." We also add a brief intuitive example in the Methods to reinforce the sign of the effect.

[Comment 3]
L94 The units for GPP/NPP should be complete.
Response 21: "/" here refers to "or". We have rephrased the term as "annual NPP m-2 and GPP m-2".

[Comment 4]
L259 remove raster
Response 22: Done.

[Comment 5]
L266 please add subheadings to the section of Analytical workflow, which is now very confusing to read.
Response 23: We have divided the previous section of Analytical workflow into four sub-sections accordingly to improve the structure.

[Comment 6]
L287 specify Type II
Response 24: We have rephrased the sentence to "To calculate p-values, we used function Anova from R package car with Type II sum of squares, which tests each main effect after accounting for all other main effects but without assuming interaction terms" in lines 329-331.

[Comment 7]

L288 but the explanatory power (R2) can determine how importance of each predictor (e.g., patch size) contributes to the predicted productivity
Response 25: We have now reported R2, p-value and slope for Fig. 2d-f and all Extended Data Figures.

[Comment 8]
L385 add quantification information for the fitting lines on the right panel, m-2 to m-2.
Response 26: See Response 25.

[Comment 9]
L397 the quantification information is missing in figure 3b, it should be clearly clarified how the models run with and without the superlinear scaling being controlled in the method (line 301-308?). It is now difficult to understand this point or the design.
Response 27: We have added RMSE in Fig. 3b to show that model predictions with superlinear scaling matches better with observations than predictions without the superlinear scaling. We rephrased text in lines 343-355 to further clarify the counterfactual comparison. We have also added this information to Fig. 3 caption: "For each biome with ≥100,000 patches (rows), points and horizontal lines show predicted total annual NPP expressed as a percentage of the observed biome total (normalized to 100%) under two scenarios: with superlinear scaling (model includes the $\log_{10}$(patch size) term; right group as filled circle) and no superlinear scaling (counterfactual in which the size term is set to the biome-mean in log space for all patches; left group as open circle). Per-area predictions (NPP m^{-2} yr^{-1}) were multiplied by each patch's true area and summed to biome totals."

[Comment 10]
L402 B) to b)
Response 27: Done.

[Comment 11]
L412 please elaborate on the window size of tree patches on the left panel and add b) on the right.
Response 28: The left panel of Fig. 4 shows two hypothetical landscapes, so it could be any window size. We have now lighted those are "hypothetical". We have also added b) on the right.

RESPONSE TO REVIEWER 3

[Comment 1]
The current paper, "Superlinear scaling of productivity with patch size across American forests", studies the impact of forest patch size on productivity and, thus, the influence of forest fragmentation. The authors have invested an enormous effort to analyse 17 million forest patches (a forest patch means here a forest, not a gap or disturbed area in a forest) in the conterminous United States. While the study offers many interesting insights, the authors could better point out its novelty compared to existing studies, consider alternative explanations (as the mere forest size might not be the primary driver of productivity) and reconsider their counterfactual modelling approach.
Response 29: We thank the reviewer for the positive and constructive feedback on our paper. We also very much appreciate the concerns raised by the reviewer and believe we were able to address all of them as outlined below.

[Comment 2]
As the reviewer's expert opinion on the counterfactual modelling component was asked for, I will start with this aspect. Counterfactual models can be empirical (1) or optimisation (2)- or

agent-based (3). Counterfactual models are widespread in providing causal inference in land system science (4). However, caution is warranted when using empirical approaches, as here, the counterfactuals might not be independent of the observed empirical data (5,6), so the effect to be analysed may still be blurred. This aspect appears to apply to the current study. The authors replaced the original forest patch size with the mean patch size in log10 space for the corresponding biome to remove the impact of the patch size on productivity. However, as one may expect that the average patch sizes will vary across biomes with large average gap sizes representing, on average, also large forest patches, the empirical patch size and productivity relation does still influence the results. Thus, the effect to be analysed may not have been eliminated from the intended counterfactual model.

Response 30: Our counterfactual is a within-biome computation contrast designed to isolate the contribution of size–productivity scaling while holding area and environment fixed. For each biome b, we predict per-area NPP for every patch twice with the fitted mixed-effects model: (i) using the observed $\log_{10}(size)$ and (ii) setting $\log_{10}(size)$ to a single constant c_b for all patches in that biome (we use the biome log-mean to keep predictions near the center of the observed distribution and avoid extrapolation). We then multiply per-area predictions by each patch's true area and sum within the same biome. The difference between these two biome totals is the estimated change in NPP attributable to removing within-biome size heterogeneity. Because all calculations and comparisons are performed within each biome, differences in the mean constant c_b across biomes do not influence the effect estimate for any given biome, nor the CONUS total (which is the sum of the biome-specific contrasts). We have clarified this estimand and the within-biome nature of the comparison in the Methods lines 344-355 and Figure 3 caption to avoid the impression that between-biome differences in average patch size drive the result.

[Comment 3]

I want to add another modelling aspect here: The study uses a 1-degree by 1-degree grid (which covers well > 1 million hectares) to account for the compound impact of a bundle of variables not explicitly addressed by the model (using a random effect model). Aside from climate, this random effect shall account for differences in the socio-economic context, including the impacts of forest management and land-use change dynamics. However, considering such large grid cells are less suitable to account for differences in often small managed (inherently low density) or leftover forests (remnants which are necessarily heavily degraded) and extensive undisturbed contiguous natural forests (natural forests dominate the USA forest cover by size).

This brings me to suggest that one might also use an alternative explanation for the results found. The study might also show the impact of the forest type and quality and the influence of the (human) disturbance or management on the canopy cover (much lower in smaller-sized forests) on productivity, but less the impact of forest size. One may expect that extensive contiguous forests are commonly largely undisturbed and thus more productive.

Response 31: The 1° × 1° grid-cell random intercept is not intended to resolve fine-scale management differences; it absorbs broad, location-specific macro-gradients (regional climate, soils/potential vegetation, disturbance history, etc.) so that the size coefficient is identified from within-cell contrasts. We agree that management intensity and forest quality can vary at scales finer than 1° and could covary with patch size. To address exactly this concern, we implemented a spatially explicit specification with high-resolution covariates (48 variables summarized by PCA) that include proxies for management and human pressure (e.g., road density, protected-area share, human development/management axes), stand structure (forest age), as well as climate, soils, and topography (see Table S2). The positive size–productivity effect persists under this stricter control and when we standardize predictions by holding all covariates at biome means and removing random effects (Extended Data Fig. 2). We have included this new analysis in discussion lines 172-177 and methods lines 357-370.

[Comment 4]
The novelty aspect could also be justified better, given that previous publications have found similar results, e.g. ref. (7,8). In addition, one could perhaps better point out that a patch is a still-standing forest and not a forest gap caused by disturbances; see ref. (9) for using "patch" to refer to a disturbed forest area.

Response 32: We agree that prior studies have linked fragmentation to lower carbon storage (refs. 7, 8). Our contribution is still novel and extends this literature in several ways. First, we focus on productivity (flows: NPP, GPP) rather than carbon stocks, and we quantify a per-area size effect that produces superlinear scaling of total productivity with patch size across the entire CONUS, not just in individual landscapes. Second, our inference relies on within–grid-cell linear mixed-effects models (and a complementary spatially explicit covariate specification with high-resolution climate/soil/topography/management/age/access variables), which control for broad spatial structure and local socio-environmental gradients; the positive size effect persists under these stricter controls and with an independent microwave proxy (VOD), addressing dependence on optical reflectance. Third, we translate the effect into configuration-driven carbon consequences by coupling the estimated size–productivity slope with the Pareto α parameter of the patch-size distribution, showing how fragmentation alone (holding total forest area fixed) shifts total NPP—an aspect not treated in refs. 7–8. Finally, we demonstrate generality by extending the analysis to global large patches.

We also acknowledge the potential ambiguity in the term "patch." In our study, a patch is a contiguous forested area delineated from the Hansen et al. map using a 30% canopy threshold and 4-connected contiguity—i.e., an area of standing forest, not a disturbance "patch" or gap as used by Maroschek et al. (ref. 9). We have made this explicit to avoid confusion.

We have made the following manuscript edits:

- Introduction in lines 51-53: "Despite evidence of reduced carbon stocks in smaller fragments at local and regional scales (citing refs. 7–8), the consequences of fragmentation for productivity (carbon sequestration rates) over short and long time horizons are still poorly understood"
- Methods in lines 269-271: "Here 'patch' denotes a contiguous forested area (≥30% canopy; 4-connected) from the Hansen map; we do not use 'patch' to mean disturbance gaps (cf. ref. 9)."

References

1. Groom, B., Palmer, C. & Sileci, L. Carbon emissions reductions from Indonesia's moratorium on forest concessions are cost-effective yet contribute little to Paris pledges. PNAS 119; 10.1073/pnas.2102613119 (2022).
2. Babatunde, K. A., Begum, R. A. & Said, F. F. Application of computable general equilibrium (CGE) to climate change mitigation policy: A systematic review. Renewable and Sustainable Energy Reviews 78, 61–71; 10.1016/j.rser.2017.04.064 (2017).
3. Knoke, T. et al. Trends in tropical forest loss and the social value of emission reductions. Nat Sustain 6, 1373–1384; 10.1038/s41893-023-01175-9 (2023).
4. Magliocca, N. R., Dhungana, P. & Sink, C. D. Review of counterfactual land change modeling for causal inference in land system science. J. Land Use Sci. 18, 1–24; 10.1080/1747423X.2023.2173325 (2023).
5. Hoover, K. D. Econometrics as observation: the Lucas critique and the nature of econometric inference. Journal of Economic Methodology 1, 65–80; 10.1080/13501789400000006 (1994).
6. Moos, K. A. The Facts and the Values of the Lucas Critique. Review of Political Economy

31, 1–25; 10.1080/09538259.2019.1586363 (2019).
7. Ma, L., Shen, C., Lou, D., Fu, S. & Guan, D. Ecosystem carbon storage in forest fragments of differing patch size. Sci Rep 7, 13173; 10.1038/s41598-017-13598-4 (2017).
8. Islam, M., Deb, G. P. & Rahman, M. Forest fragmentation reduced carbon storage in a moist tropical forest in Bangladesh: Implications for policy development. Land Use Policy 65, 15–25; 10.1016/j.landusepol.2017.03.025 (2017).
9. Maroschek, M., Seidl, R., Poschlod, B. & Senf, C. Quantifying patch size distributions of forest disturbances in protected areas across the European Alps. Journal of Biogeography 51, 368–381; 10.1111/jbi.14760 (2024).

RESPONSE TO REVIEWER 1

[Comment 1]
The spatio-temporal pattern of forest fragmentation is a hot topic in ecological research. The authors analyzed the relationship between forest patches and productivity in the continental United States. Their results revealed that the larger the patch, the higher the vegetation reflectance, and the higher the per-area net primary productivity. The total productivity showed a super-linear relationship with the patch size. While this research has significant implications for the implementation of forest protection policies, I do not think it meets the criteria for publication at this stage.
Response 1: We thank the reviewer for the positive and constructive feedback on our paper. We also very much appreciate the concerns raised by the reviewer and believe we were able to address all of them as outlined below.

[Comment 2]
First and foremost, it is well known that the productivity per unit area of small forest patches is lower compared to large forest patches. What exactly is your contribution? You mentioned that "our contribution is to quantify the continent-wide per-area size effect, demonstrate superlinear scaling of total NPP with size, and link it to the Pareto α configuration parameter to show how fragmentation alone (holding area fixed) alters total NPP." However, I still have some confusion about this part and haven't clearly seen where your increment lies. A very important reason for this is that the authors might have mixed the methods and results together, and the description of the methods is overly mathematical (e.g., L431-450). It is suggested to separate the methods and results for explanation, and to express and explain the purpose, function and principle of the methods in a more understandable way.
Response 2: Thank you for this important comment. We agree that reduced functioning in small forest fragments—often discussed in terms of edge effects and degradation—is well established qualitatively. However, we believe our study makes two substantive contributions that have not been addressed explicitly in prior work, and we acknowledge that these were not sufficiently clear in the original manuscript.

First, most previous studies linking patch size or fragmentation to ecosystem function focus on carbon stocks or biomass density (*1-5*), not productivity (NPP/GPP). Biomass represents a standing stock integrated over decades, whereas productivity reflects current carbon sequestration rates, which can diverge from biomass patterns due to age structure, mortality, management, and disturbance history. To our knowledge, no previous study has quantified how per-area NPP scales with forest patch size across a large spatial extent, let alone at continental or global scale. A major contribution of our work is therefore to demonstrate—using independent, flux-based proxies (NPP, GPP, etc.)—that patch size exerts a systematic influence on carbon uptake rates, not only on standing carbon pools.

Second, we quantitatively show that this per-area size effect leads to superlinear scaling of total productivity with patch size. Prior work generally compares mean values among fragment classes or reports local contrasts (*4*), but does not frame the result as a scaling relationship or evaluate its aggregate implications. By explicitly estimating the slope of the per-area size–productivity relationship and propagating it across the full patch-size distribution (>17 million patches in CONUS), we show that hectares in large patches are not functionally equivalent to hectares in small patches, and that this nonlinearity accounts for a substantial fraction of total forest NPP.

We have rephrased lines 46–56 of the Introduction to more clearly distinguish our study from prior fragmentation research that has focused primarily on biomass, and to clarify why analysing productivity provides complementary and novel insight. We also acknowledge the reviewer's concern that the original manuscript intermingled methodological description with interpretation and that some methodological sections were overly mathematical in the main

text. In response, we have (i) streamlined the brief methodological summaries embedded within each Results section (lines 83–220), and (ii) revised the main Methods section to emphasize the rationale and conceptual purpose of each analytical step, rather than detailed mathematical formulation (lines 234–419).

We hope these changes clarify both the contribution of our study and the logic of the analytical framework, and we thank the reviewer for helping us sharpen this distinction.

[Comment 3]
Secondly, this study mainly explains the phenomenon of the superlinear relationship between forest patch size and productivity. However, the analysis of the underlying mechanisms is still relatively weak, as forest patch size alone may not be the main driver of productivity. Although the authors have added explanations of the potential mechanisms in this regard, this study has not conducted an analysis based on first hand data.
Response 3: We thank the reviewer for this important comment regarding the mechanistic interpretation of the superlinear patch size–productivity relationship. We agree that forest patch size is not a direct physiological driver of productivity, but rather an integrative landscape attribute that operates through a set of well-established biophysical and ecological processes.

To strengthen the mechanistic foundation of the study using first-hand data, we have added two complementary analyses based on the same ~240,000 randomly sampled forest pixels (Fig. S4) used in the main models. First, our random forest analysis explicitly evaluates the relative importance of patch size alongside climate, topography, and soil variables. We show that patch size consistently emerges as one of the strongest predictors of NPP (Fig. 4c) — second only to major climatic drivers—indicating that its effect is not a statistical artifact but reflects information not captured by environmental covariates alone.

Second, we conducted a spatially explicit edge-effect analysis at the pixel level. Using direct measurements of distance to forest edge, we demonstrate that (i) smaller patches are geometrically dominated by edge environments (having higher edge to area ratio, Extended Data Fig. 1), (ii) productivity increases systematically with distance from the edge within large patches (Extended Data Fig. 3a,b), and (iii) these interior–edge gradients persist after detrending NPP for topographic effects (Extended Data Fig. 3c,d). These analyses provide empirical support, based on first-hand spatial data, that edge exposure constitutes a key mechanistic pathway linking patch size to per-area productivity.

We have revised the main text to include the methods, results and interpretation of these two analyses in lines 123-137, 152-167, 340-397. We have also clarified that patch size acts as an indirect driver that integrates multiple direct processes (edge effects, hydrology buffering, and species-area relationship etc.) in lines 152-191, and we now include a conceptual pathway figure to explicitly illustrate these relationships (Extended Data Fig. 4). While we do not claim to isolate all individual mechanisms, the added analyses substantially strengthen the mechanistic interpretation and demonstrate that the observed scaling arises from measurable, spatially explicit processes operating within forest patches.

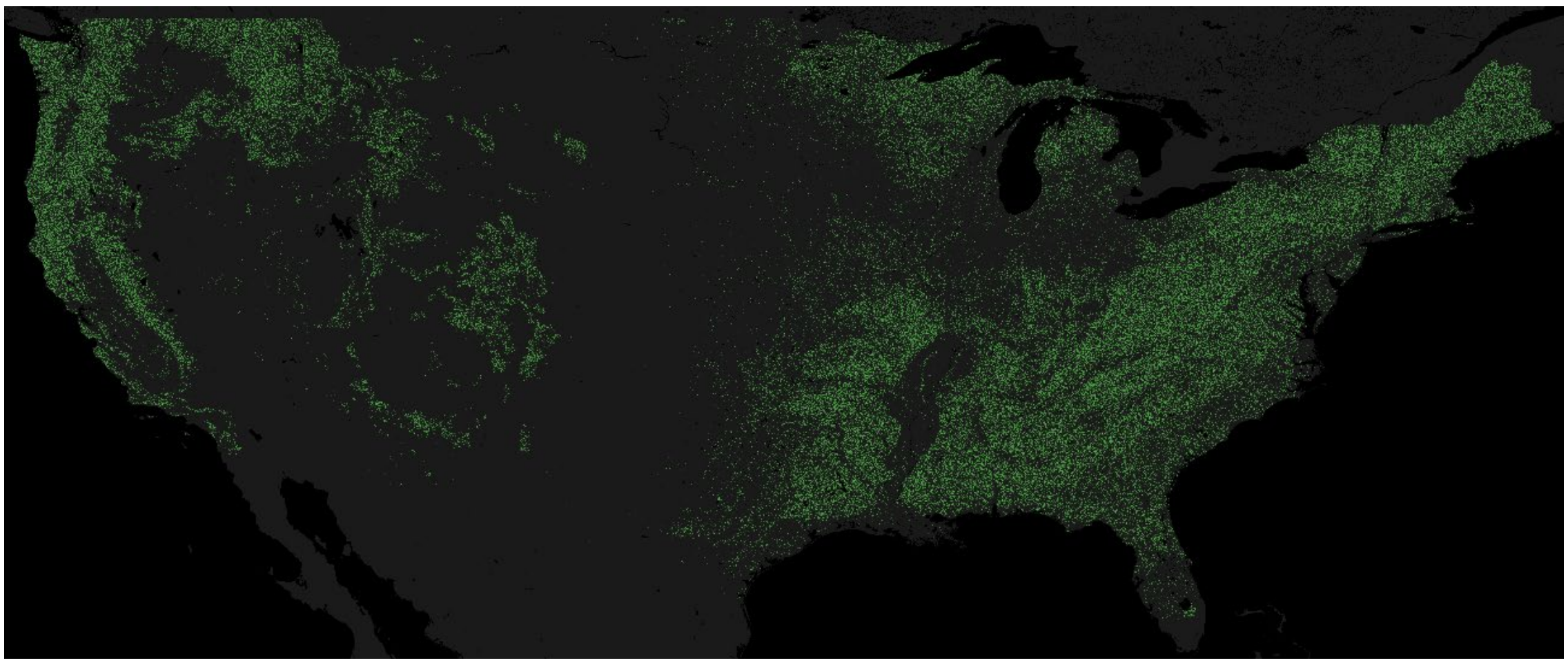

Fig. S4: Green points show the locations of the randomly sampled 239,633 30 m × 30 m forest pixels across the CONUS used in the random forest models and edge-effect analyses. Sampling spans all major forested regions, ensuring broad geographic and environmental coverage for model inference.

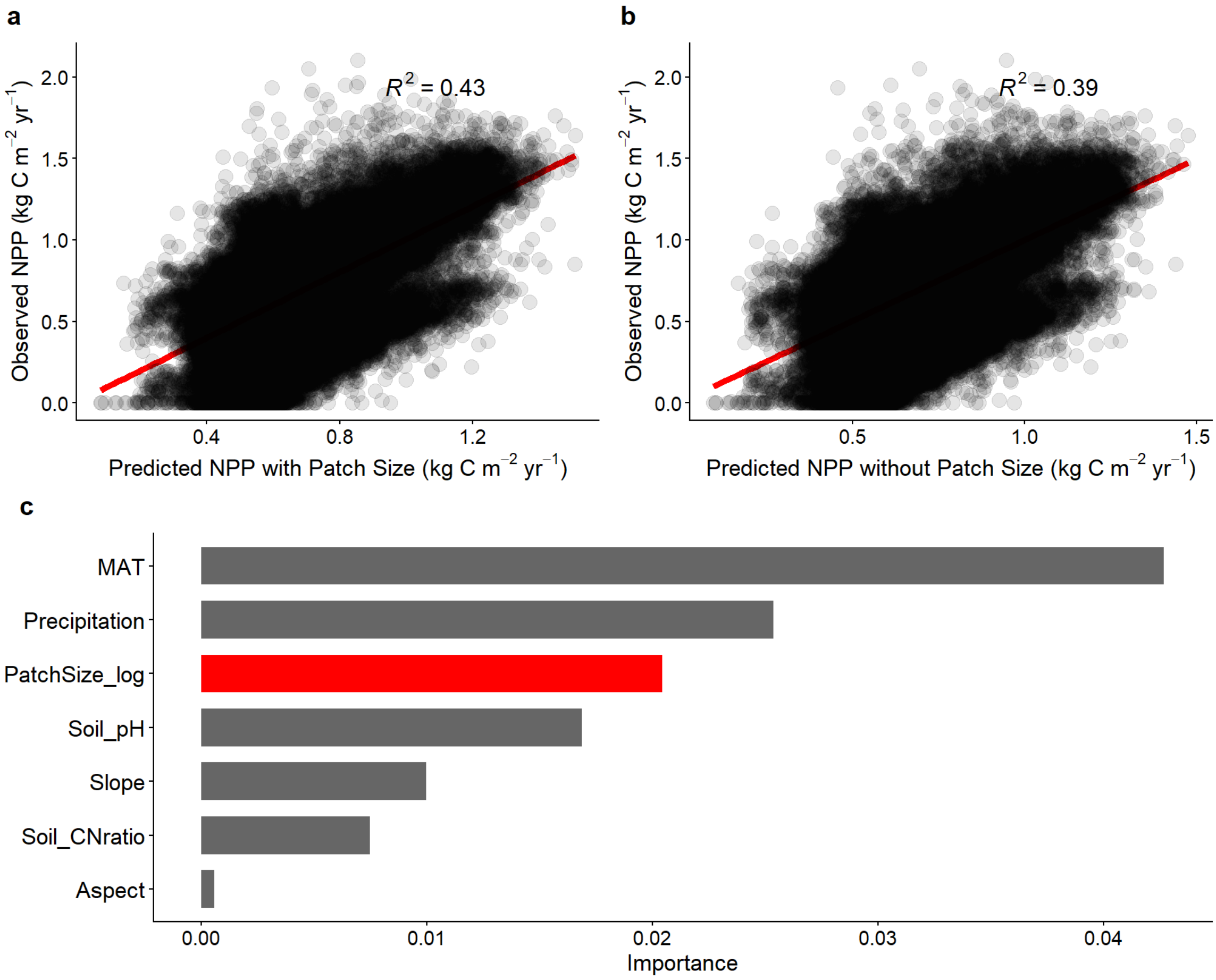

Fig. 4: Contribution of forest patch size to predicting forest productivity. a) Observed versus predicted net primary productivity (NPP) from a random forest model that includes forest patch size (log-transformed) together with climatic, topographic, and soil covariates. The red line shows the fitted linear relationship, and R^2 indicates overall model explanatory power. b) As in a, but for an otherwise identical random forest model excluding patch size as a predictor. Model performance is reduced relative to a, indicating that patch size contributes independent explanatory power beyond

environmental covariates. c) Permutation-based variable importance from the random forest model shown in a. Forest patch size (log_{10}-transformed; highlighted in red) ranks among the most influential predictors of NPP, comparable in magnitude to key climatic and edaphic variables. Random forest models are trained on a dataset of >230,000 randomly sampled forest pixels across the CONUS.

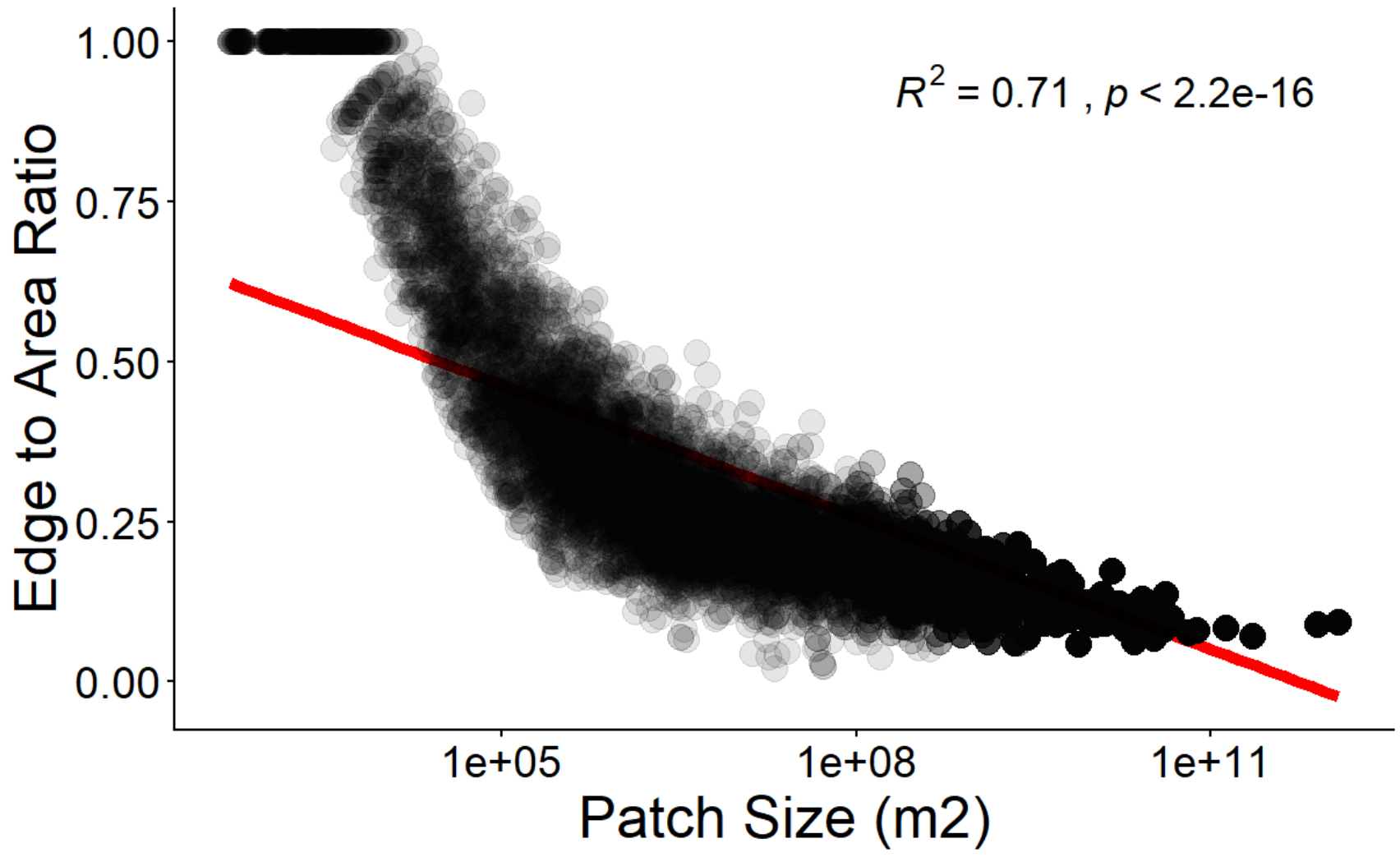

Extended Data Fig. 1: Relationship between forest patch size (m², log scale) and the edge-to-area ratio, a geometric measure of edge exposure, across forest patches in the conterminous United States. Larger patches exhibit a sharply lower proportion of edge per unit area, reflecting the fundamental geometry of fragmentation. The fitted regression line (red) highlights a strong negative relationship ($R^2 = 0.71$, $p < 2.2 \times 10^{-16}$), indicating that small patches are disproportionately dominated by edge environments. This scaling relationship provides a mechanistic link between fragmentation, elevated edge influence, and reduced per-area productivity in small forest patches.

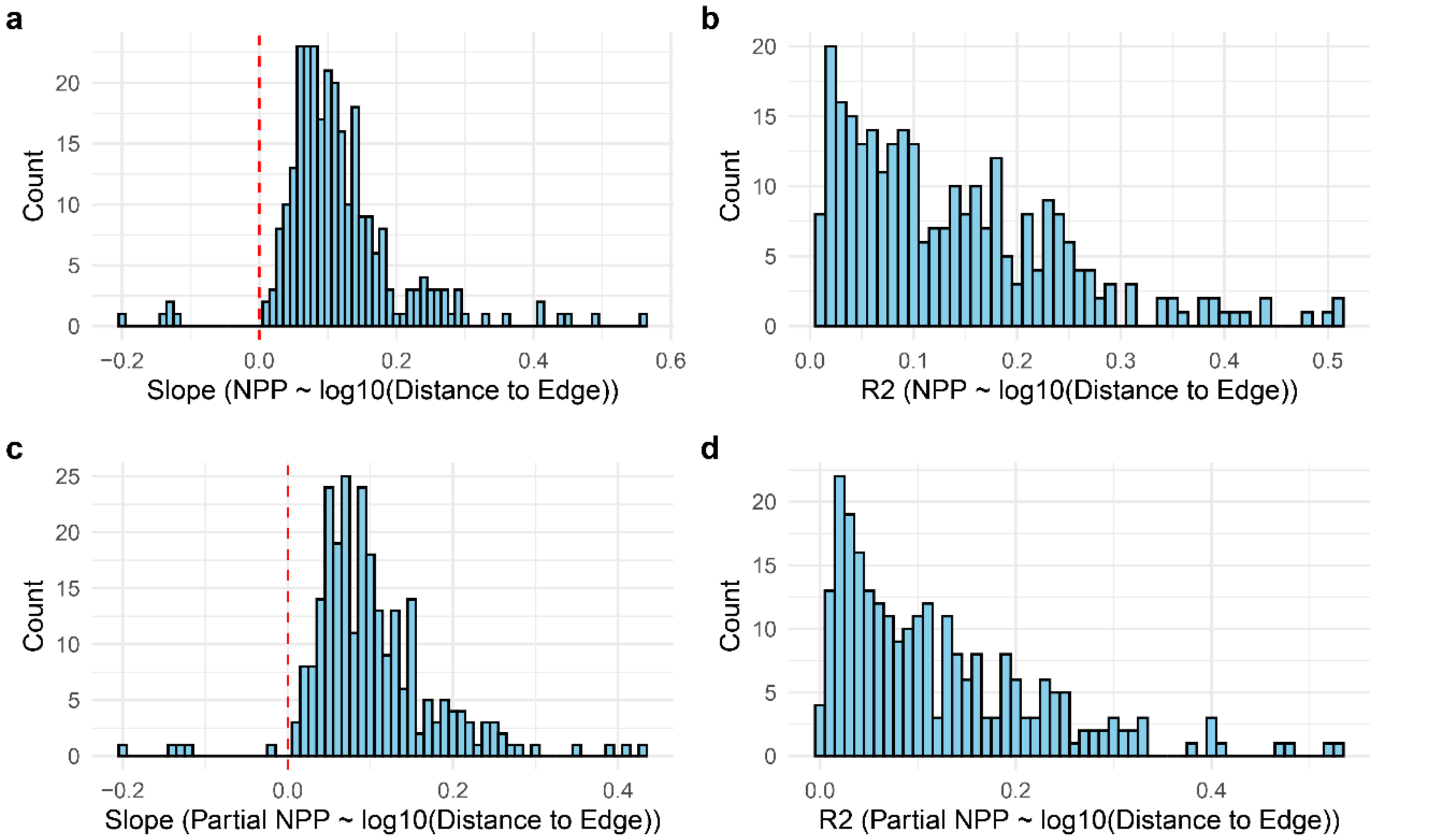

Extended Data Fig. 3: Histograms summarize the slope and coefficient of determination (R^2) from patch-level regressions of forest productivity against $\log_{10}$(distance to forest edge) for all forest patches with ≥50 sampled pixels across the conterminous United States. Panels a–b show results using raw annual NPP, while panels c–d show results after detrending NPP for topographic variation (slope, elevation, and aspect). Red dashed lines in panels a and c indicate zero slope. Across patches, estimated slopes are generally positive, indicating higher NPP in interior forest pixels than edge pixels.

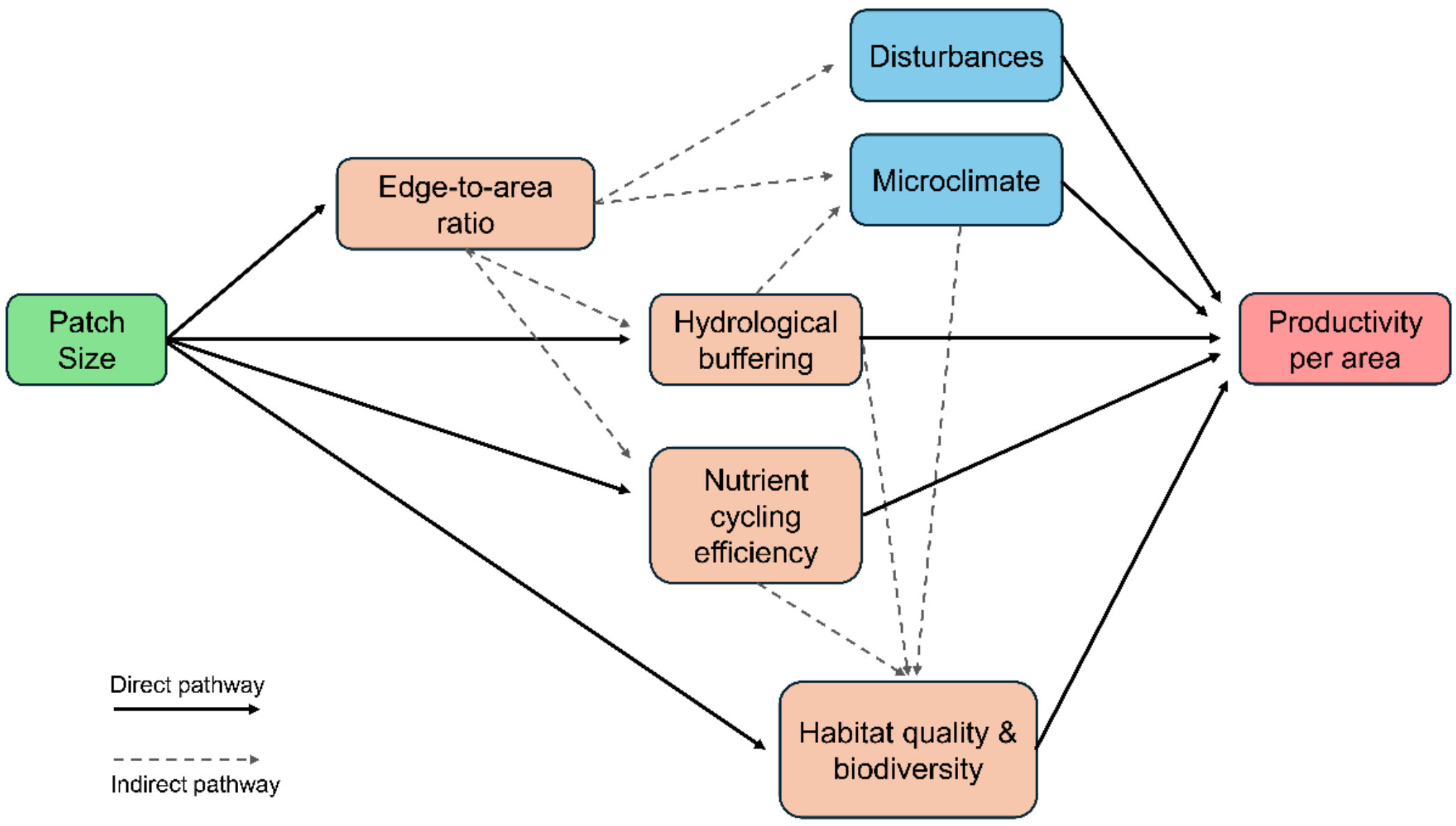

Extended Data Fig. 4: Conceptual pathways linking forest patch size to per-area productivity. The diagram summarizes direct and indirect mechanisms by which forest patch size influences productivity per unit area. Larger patches reduce the edge-to-area ratio, limiting exposure to edge-

related microclimatic stress and disturbances(*4, 6-9*). Patch size also directly enhances hydrological buffering(*10, 11*), nutrient cycling efficiency(*12-14*), and increases habitat quality and biodiversity(*15-20*), which together regulate moisture availability, nutrient retention, and ecosystem functioning. Dashed arrows indicate indirect or cascading pathways mediated by edge effects, microclimate, and disturbance, while solid arrows represent direct effects. Collectively, these pathways provide a mechanistic framework linking forest fragmentation to reduced productivity in small patches.

[Comment 4]
Furthermore, the spatial resolution of VOD is quite coarse. Within a 9-km range, it is highly likely that both forest and non-forest components (such as grassland and shrubland) are included. You also applied a screening rule based on forest coverage: "we applied an edge-exclusion filter by removing NPP/GPP/VOD pixels with <95% forest cover to reduce contamination from non-forest areas." Therefore, the VOD pixels you selected are more likely to be those of larger forest patches, as can be seen from the frequency distribution of patch sizes in Fig. 1 of the main text. It shows that NPP/GPP/NDVI are mostly within 1e06 (< 1e06), but VOD has a significant portion beyond 1e08. This could lead to inconsistencies. Thus, there is a possiblity that the inclusion of VOD may have limited explanatory power for the argument that "fragmentation and small patches of forests lead to a decline in productivity."
Response 4: We agree that the coarse spatial resolution of VOD (9 km) presents challenges, and we view the VOD analysis as a robustness check, not as the primary basis for our conclusions.

First, the reviewer is correct that at 9-km resolution, individual VOD pixels may include mixed land-cover types. This is precisely why we applied a strict forest-cover threshold (≥95%) and restricted the analysis to large forest patches (≥81 km²), see supplementary text in lines 11-29. This screening necessarily reduces the representation of small patches and shifts the size distribution toward larger forests. However, this was a deliberate and conservative design choice to ensure that retained VOD pixels are genuinely forest-dominated, rather than a by-product of the analysis. Importantly, all inferences drawn from VOD are therefore conditional on large patches, and we do not interpret the VOD results as evidence about very small fragments.

Second, because the VOD analysis is restricted to large, interior forest areas, it would tend to attenuate, not inflate, any size–productivity relationship driven by fragmentation or edge effects. Despite this conservative filtering, we still observe a fairly strong positive size–productivity relationship ($R^2 \approx 0.19$ in CONUS), indicating that the signal persists even when edge-dominated and heavily fragmented patches are excluded. This strengthens the interpretation that patch size is associated with productivity differences.

Third, the purpose of including VOD is not to balance the full patch-size distribution or replace Landsat-based productivity metrics. Instead, it serves to address a specific concern about shared dependence on optical reflectance between Landsat-derived patch size and productivity (NPP/GPP/NDVI). VOD is physically and algorithmically independent of optical data, and the fact that it reproduces the same qualitative scaling pattern—despite coarser resolution, a reduced sample, and more conservative filtering—demonstrates that our primary findings are not an artifact of Landsat reflectance.

Finally, our main conclusions regarding fragmentation and productivity rely on results derived from high-resolution (30 m) products covering the full patch-size spectrum and from

models that explicitly control for environmental and spatial structure. The VOD analysis is presented as supporting evidence, with its scope and limitations clearly stated, and not as the sole or dominant line of inference.

We have revised the manuscript to clarify this role of VOD more explicitly in lines 139-150, emphasizing that it provides an independent robustness check for large forest patches, rather than a comprehensive assessment of small-patch productivity effects.

[Comment 5]
L13: The first sentence of the Abstract does not place your research within a specific research context nor does it imply the value of your research on the forest fragmentation.
Response 5: We agree that the opening sentence of the Abstract should more clearly position our study within the broader research context on forest fragmentation and carbon cycling. We have revised the first sentence to explicitly frame forest fragmentation as a key uncertainty in forest-based carbon accounting: "Forest fragmentation is increasing globally and is widely recognized as a major threat to biodiversity, yet its consequences for forest carbon sequestration remain poorly quantified".

[Comment 6]
L65-87: These three paragraphs are more like the content of the method section.
Response 6: We have rephrased and condensed the relevant text into a single paragraph that more clearly summarizes how our study addresses this gap. The revised paragraph now reads in lines 74-81: "Here, we quantify forest productivity and patch size across the conterminous United States (CONUS) by analysing 17 million forest patches (Fig. 1a, Fig. S1). As observed in other regions(*21*), patch sizes follow a heavy-tailed Pareto distribution, where the vast majority of patches are small ($<10^4$ m^2) and collectively contribute little to total forest area (Fig. 1b). We then estimate how forest productivity varies with patch size, explicitly testing the magnitude and significance of the patch-size effect while controlling for spatial and environmental heterogeneity. Finally, we extend the analysis globally using coarser-resolution productivity products to assess whether the observed scaling relationship generalizes beyond CONUS.
".

[Comment 7]
L96: The number of the supplementary figure is 65 right from the beginning? Please renumber the figures in accordance with the logical sequence described in the main text.
Response 7: We have reordered the supplementary figures and removed redundant figures to improve clarity and focus.

[Comment 8]
L37: L107-109: What do you want to express if you put this sentence at the end of the paragraph?
Response 8: We have deleted this redundant sentence.

[Comment 9]
L471: Fig.1: it is recommended that you marked with X axis label. Besides, as the second reviewer mentioned, the fitting effect between original data of patch size and GPP/NPP/NDVI is very weak (R square) from density plot. Why can you then conclude your title of manuscript (superlinear scaling of productivity with forest patch size) when taking the logarithm of the patch size? In other words, what is the basis for your data transformation?

Can the line graph obtained from the transformed patch size data demonstrate superlinear correlation?
Response 9: X axis labels have been added to all figures with X axis as Patch Area. We thank the reviewer for this important question. Our conclusion of superlinear scaling does not rely on simple bivariate fits between raw patch size and productivity. Forest patch size spans more than seven orders of magnitude and follows a heavy-tailed (approximately Pareto) distribution (Fig. 1b), for which linear relationships on the raw scale are neither statistically appropriate nor biologically meaningful.

The $\log_{10}$ transformation of patch size is therefore theory-driven and standard in scaling analyses, where productivity is expected to follow a power-law relationship with size. Modeling productivity as a function of $\log_{10}$(patch size) allows us to test whether total productivity scales superlinearly with patch size, which implies increasing per-area productivity in larger patches.

Crucially, the positive size–productivity relationship is robust across multiple independent analyses, including biome-specific mixed-effects models (Fig. 2), random forest models controlling for climate, soil, and topography (Fig. 4; Fig. S7), and global analyses using MODIS NPP and GPP (Fig. 5). The global results indicate that superlinear scaling is a widespread feature of forest landscapes, with stronger relationships in tropical-dominated regions (South America, Africa, Asia, and Oceania; $R^2 \approx 0.10–0.44$) than in temperate regions such as Europe and North America ($R^2 \approx 0.007–0.031$). Potential reasons for these continental differences are discussed in lines 209-220.

Our inference is therefore based on consistent slopes and effect sizes across datasets, regions, and modeling frameworks, rather than on raw-scale R^2 values alone.

RESPONSE TO REVIEWER 2

[Comment 1]
Thank you for the substantial revisions. The authors have basically addressed my concerns in the revised manuscript. I believe that the result is robust with regard to the positive relationships between NPP/GPP and forest patch sizes, although this relationship is much clearer at the biome-level than at the patch level.
Response 10: Thank you for this constructive assessment. We appreciate the reviewer's careful evaluation and agree that the size–productivity relationship is most clearly expressed at aggregated (biome-level) scales, while remaining detectable but noisier at the patch level due to fine-scale heterogeneity. We are grateful for the reviewer's acknowledgment of the robustness of our results and believe the revisions have helped clarify the remaining concerns.

[Comment 2]
Regarding potential mechanisms, this table 2 added to the understanding of the empirical relationship NPP-patch, where respiration might be worth mentioning due to the lack of mechanism analysis. The patch size is an indirect factor that affects carbon uptake by forcing these direct processes such as changes in hydrological activities/nutrients, microclimate conditions that affect carbon cycles. The edge effect is also an indirect factor. A pathway depicting these direct and indirect relationships between these variables would be thus helpful. Moreover, it would be better to refine the highlighted points, as the issue of fragmentation-induced carbon loss has been overused in existing literatures. btw, the x-axis titles are missing in the figure 2 and the similar ones.

Response 11: Thank you for these insightful suggestions. We agree that forest patch size is an indirect driver of carbon uptake and that its effects operate through direct biophysical processes. In response, we have clarified and expanded the discussion of potential mechanisms in the revised manuscript. Following the reviewer's recommendation, we have added a new conceptual figure (Extended Data Fig. 4) that explicitly depicts the direct and indirect pathways linking patch size, edge exposure, intermediate ecological processes (e.g., microclimate, hydrology, nutrients, biodiversity), and per-area productivity. This schematic clarifies the proposed causal structure of our hypothesis and situates edge effects as a mediating mechanism rather than a direct driver.

In addition, we have refined the highlighted points to more clearly distinguish our contribution from prior fragmentation–carbon studies in lines 46-56 of the introduction. Specifically, we now emphasize that our analysis focuses on per-area productivity scaling and configuration effects independent of forest-area loss, rather than reiterating general statements about fragmentation-induced carbon stock loss.

Finally, we thank the reviewer for noting the missing x-axis labels. These have now been added to Fig. 2 and all similar figures to improve clarity and consistency.

We appreciate these suggestions, which have significantly improved the clarity, rigor, and presentation of the manuscript.

[Comment 3]
The authors properly addressed the major weaknesses raised. They summarised several aspects of the potential mechanisms underlying the superliner scaling and clarified the rationale behind using CONUS to exploit the relationships between carbon sinks and patch sizes. They further strengthened the superliner scaling relationships by extending the forest extents from CONUS to a global scale. This is a worthwhile test. Here, additional analysis is suggested, including quantitative information that explains the superliner scaling effects or shows the contribution that superliner scaling makes to future studies. For example, a potential test could be conducted to see if a model including the superliner effect could improve the prediction of forest carbon sequestration. This is because, at this stage, the section on mechanism is highly narrative-based, making it seem very trivial compared to the primary documentation of the superliner relationship. I further suggest including these relevant maps/figures (such as global forest cover, patch distributions and biome divisions, etc) in addition to the extended figs.5-10 in the supplementary documents to make it easier to interpret these results visually.
Response 12: We agree that quantitative evidence linking the observed superlinear scaling to predictive performance and underlying processes is essential.

In response, we have strengthened the mechanistic section by adding two complementary quantitative analyses (see also Response 3). First, we explicitly test whether accounting for patch size improves predictions of forest carbon uptake using random forest models trained on ~240,000 randomly sampled forest pixels across CONUS. These models show that including patch size significantly improves predictive performance relative to otherwise identical models that include only climatic, topographic, and soil covariates (Fig. 4a–b), and that patch size ranks among the most influential predictors of NPP (Fig. 4c), only second to climatic variables. This provides a quantitative demonstration that the size-dependent (superlinear) effect contributes meaningful explanatory and predictive power beyond environmental controls.

Second, to move beyond narrative mechanisms, we conducted pixel- and patch-level edge-effect analyses that directly quantify one key pathway linking fragmentation to productivity. We show (i) a strong geometric relationship between patch size and edge-to-area ratio

(Extended Data Fig. 1), and (ii) consistently positive relationships between productivity and distance to edge across patches nationwide (Extended Data Figs. 2–3). These results provide first-hand, spatially explicit evidence that interior–edge gradients in productivity are widespread and help explain why per-area productivity increases with patch size.

Together, these analyses quantitatively link superlinear scaling to both improved carbon prediction and specific ecological processes, strengthening the mechanistic foundation of the study while keeping the primary focus on documenting a robust, large-scale scaling relationship. We have also added maps of forest patch distributions (CONUS and global), biome classifications, and sampled forest pixels to improve transparency and interpretation (Fig. S1, S3-5).

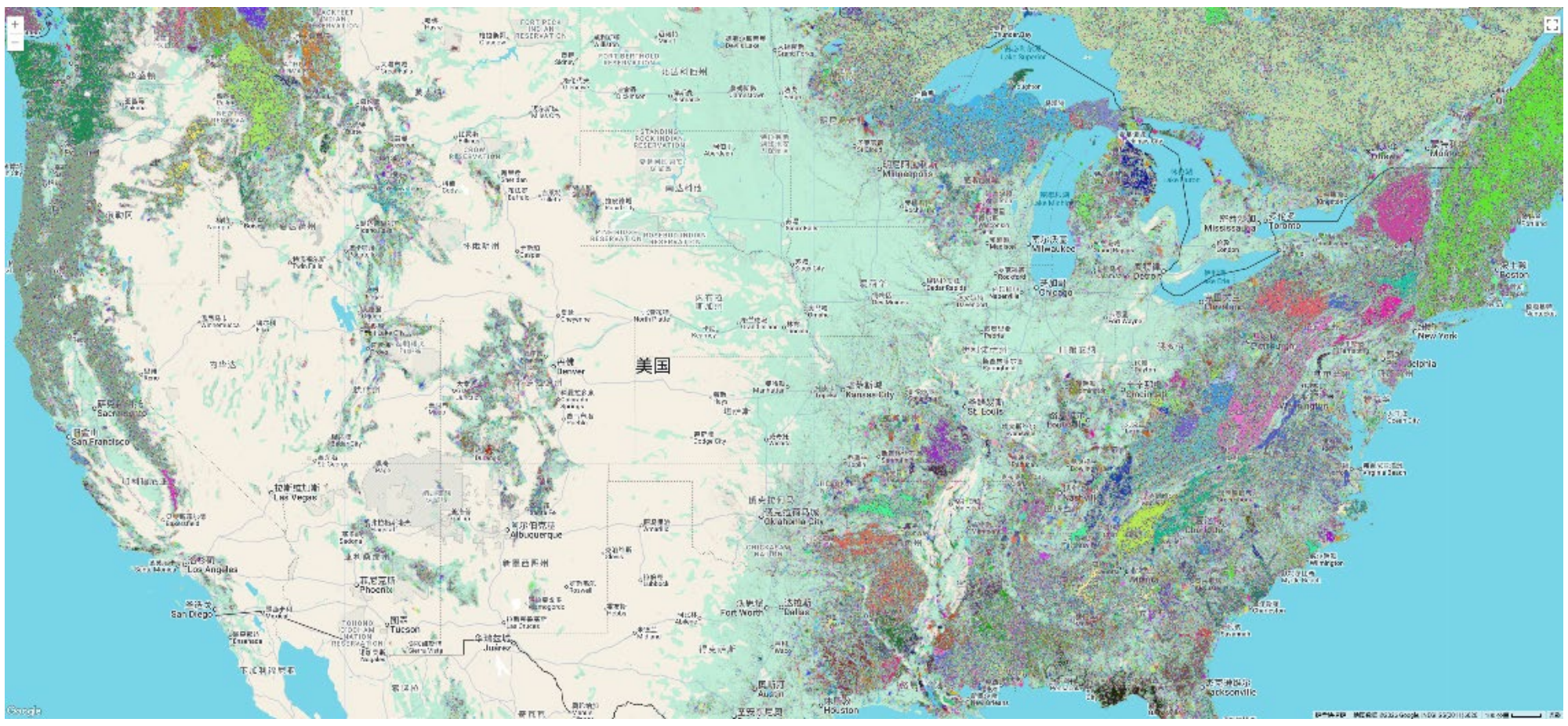

Fig. S1: Spatial distribution of forest patches across the conterminous United States (CONUS) derived from Google Earth Engine. Each color denotes an individual forest patch.

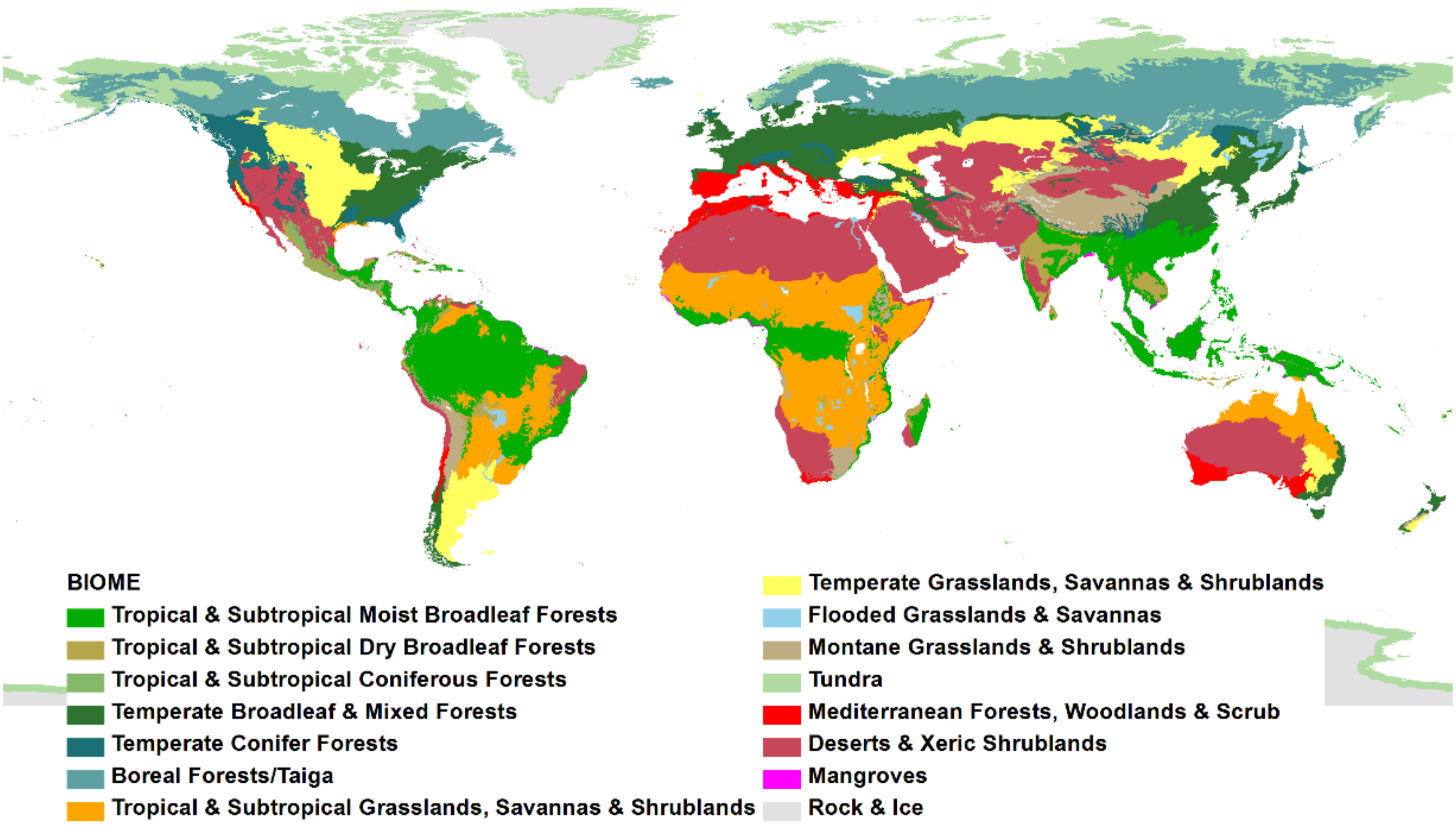

Fig. S3: Global biome classification used in this study, based on the terrestrial biome framework of Olson et al. (2001)(*22*).

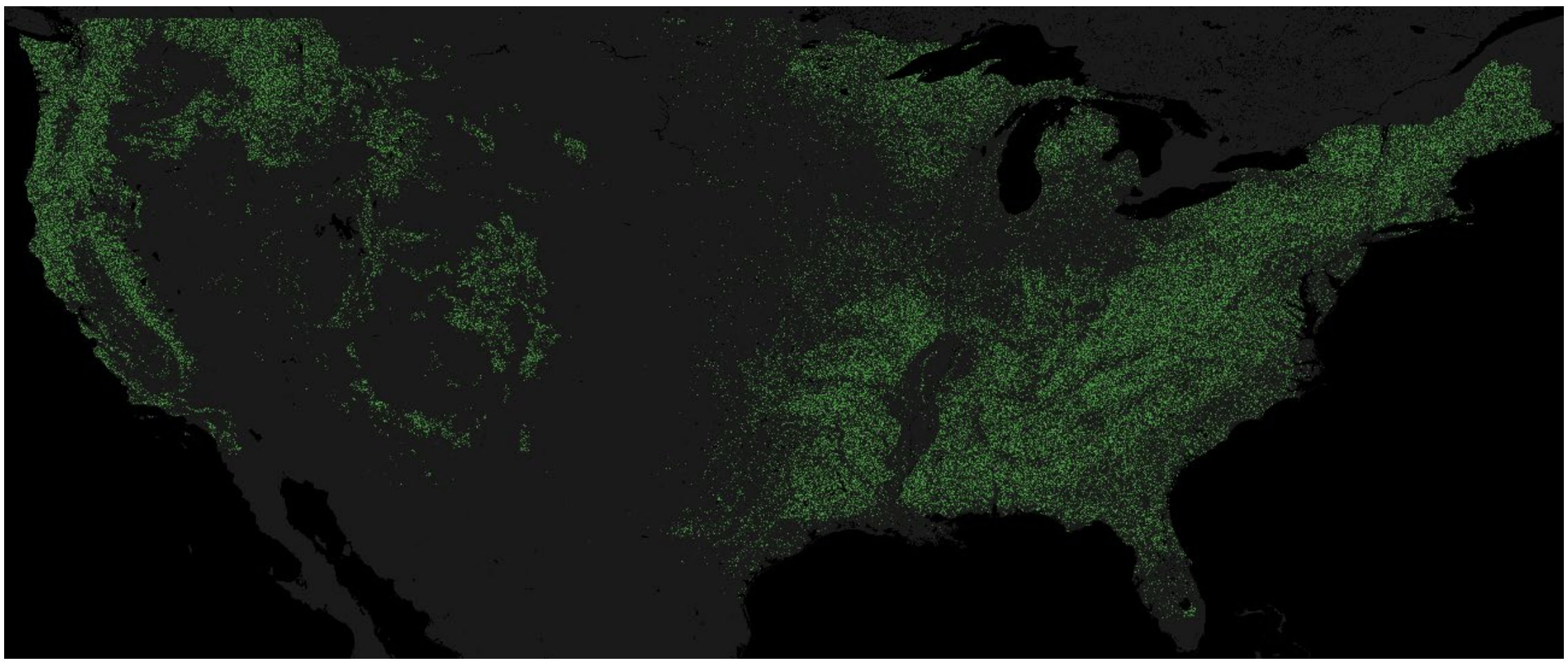

Fig. S4: Green points show the locations of the randomly sampled 239,633 30 m × 30 m forest pixels across the CONUS used in the random forest models and edge-effect analyses. Sampling spans all major forested regions, ensuring broad geographic and environmental coverage for model inference.

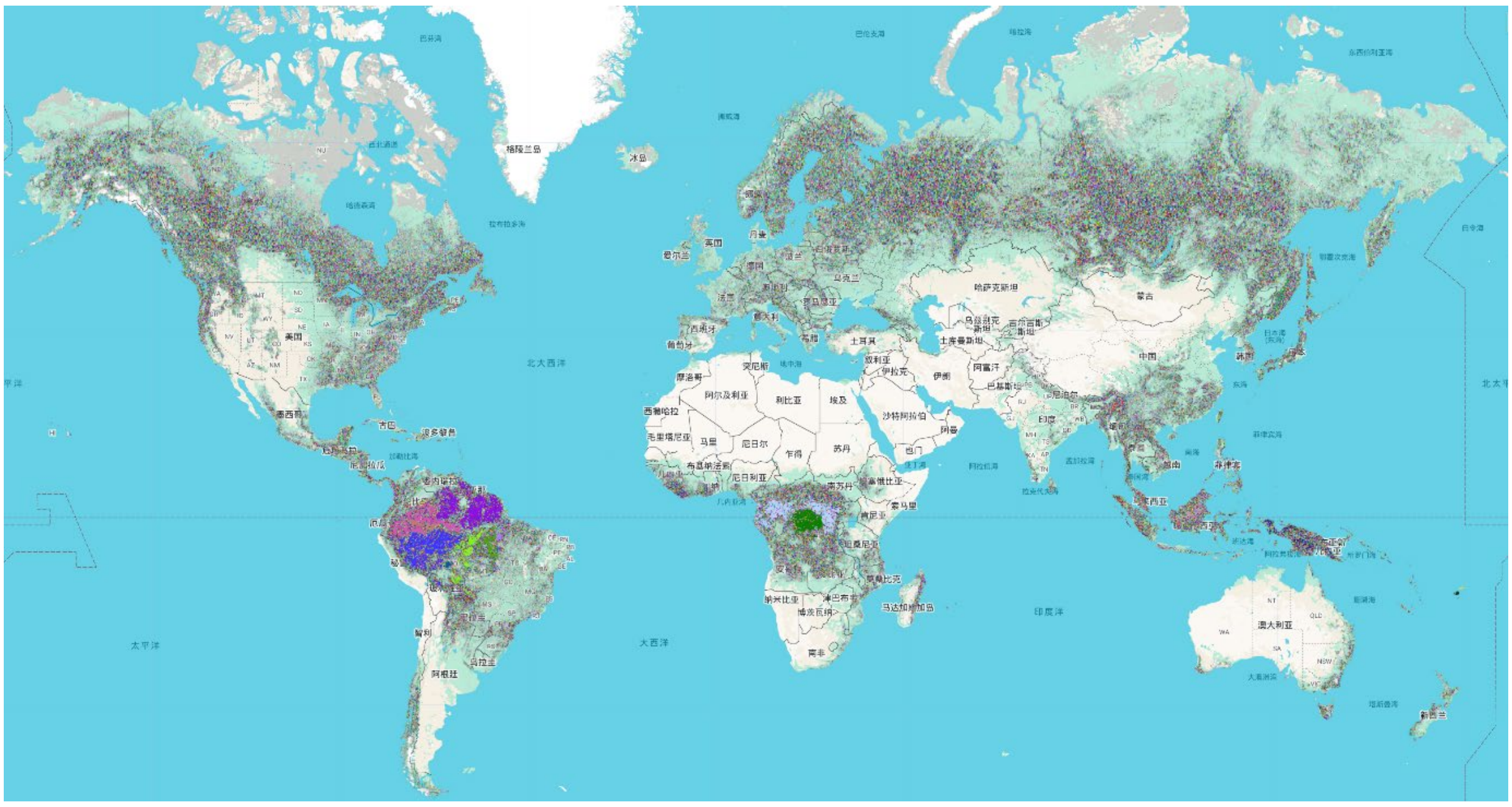

Fig. S5: Global distribution of forest patches derived from Google Earth Engine. Each color represents an individual forest patch. Patches shown are filtered according to the criteria described in *Methods and Materials* for the global analysis.

RESPONSE TO REVIEWER 3

[Comment 1]
The revision of the paper, “Superlinear scaling of productivity with patch size across American forests", addressed many of my comments nicely, while the consistency of the conclusions and the choice of the counterfactual still do not convince the reviewer fully.
Response 13: We thank the reviewer for their careful re-evaluation of the revised manuscript and for acknowledging that many of the original concerns have been addressed.

We appreciate that questions remain regarding the consistency of the conclusions and the choice of the counterfactual, and believe we were able to address all of them as outlined below.

[Comment 2]
The choice of a counterfactual is always subjective and depends on what one would like to investigate. Here, the authors use a $\log_{10}$(patch size) coefficient to create a counterfactual and interpret this as an average within-cell causal effect of increasing patch size on per-area productivity. The aim is to quantify the extra carbon uptake attributable to the superlinear size-productivity relationship. This is scientifically interesting and, of course, not wrong. However, by using the average effect of increasing patch size, this counterfactual does not remove the effect of the patch size on the productivity fully, as the cumulative NPP is still the one affected by patch size. Consequently, the counterfactual does not solely refer to NPP values without any fragmentation. A counterfactual to support the policy statements of the study, i.e. that the NPP loss incurred by fragmentation shall be considered in future forest protection policies, requires a counterfactual without any impact of fragmentation.
The simplest such counterfactual would be the maximum NPP of 0.8 kg m-2 for large and unfragmented forests. Compared to this counterfactual, the highest fragmentation and the smallest patches are associated with an NPP of 0.58 kg m-2 (Tab. 1), which we shall interpret as a reduction of NPP by 27.5% (1-0.58/0.8). This number already constitutes a strong difference compared with the reported increase of NPP by 37.83% by comparing maximum with minimum productivity. However, the demonstrated consideration assumes that all forests would be maximally fragmented, while very small forest patches cover only around 1% of the total analysed area (Fig. 1b). When taking the area covered by the different patch sizes (blue line in Fig. 1b), one may roughly find a total reduction of NPP by 13% caused by fragmentation effects. However, my consideration is not exact, as I read the numbers needed from Figures 1b and 2a. Nevertheless, I would consider this type of effect interesting to inform policy concerning the impact of fragmentation, which is quite different compared to the effect of 32% as resulting from the author's counterfactual. It would be more suitable to support the policy statements of the study.
Response 14: We thank the reviewer for this careful and constructive assessment of the counterfactual framework. We fully agree that counterfactuals are inherently subjective and must be closely aligned with the specific question they are intended to address.

In the revised manuscript, we have therefore adopted precisely the type of counterfactual the reviewer recommends and clarified its interpretation accordingly. Rather than relying on an average marginal effect derived from the $\log_{10}$(patch size) coefficient, our revised counterfactual analysis (Fig. 3) holds total forest area constant and removes the influence of fragmentation entirely by assigning uniform per-area productivity values across all forest area.

Specifically, the least fragmented scenario assumes that all forest area functions as large, contiguous patches and is assigned a per-area NPP of 0.80 kg C m^{-2} yr^{-1}, corresponding to the empirically observed mean NPP of the largest patch size class listed in Table 1. The most fragmented scenario assigns all forest area a per-area NPP of 0.58 kg C m^{-2} yr^{-1}, corresponding to the smallest patch size class. The realized scenario integrates the observed patch-size distribution by weighting patch-specific productivity by area. This approach explicitly removes fragmentation effects in the upper-bound scenario and therefore directly addresses the reviewer's concern.

Under this configuration-based counterfactual, we find that existing fragmentation reduces total annual NPP across CONUS by 0.16 Gt C yr^{-1} or ~14% relative to an unfragmented upper bound, closely matching the reviewer's own back-of-the-envelope estimate based on

Fig. 1b. We have revised the text in lines 21-23, 103-121, 310-338 to reflect the updated counterfactual analysis in abstract, results, interpretations and methods.

We believe this revised counterfactual is both conceptually aligned with policy questions and empirically grounded in the observed patch-size distribution, and we thank the reviewer for prompting this important clarification.

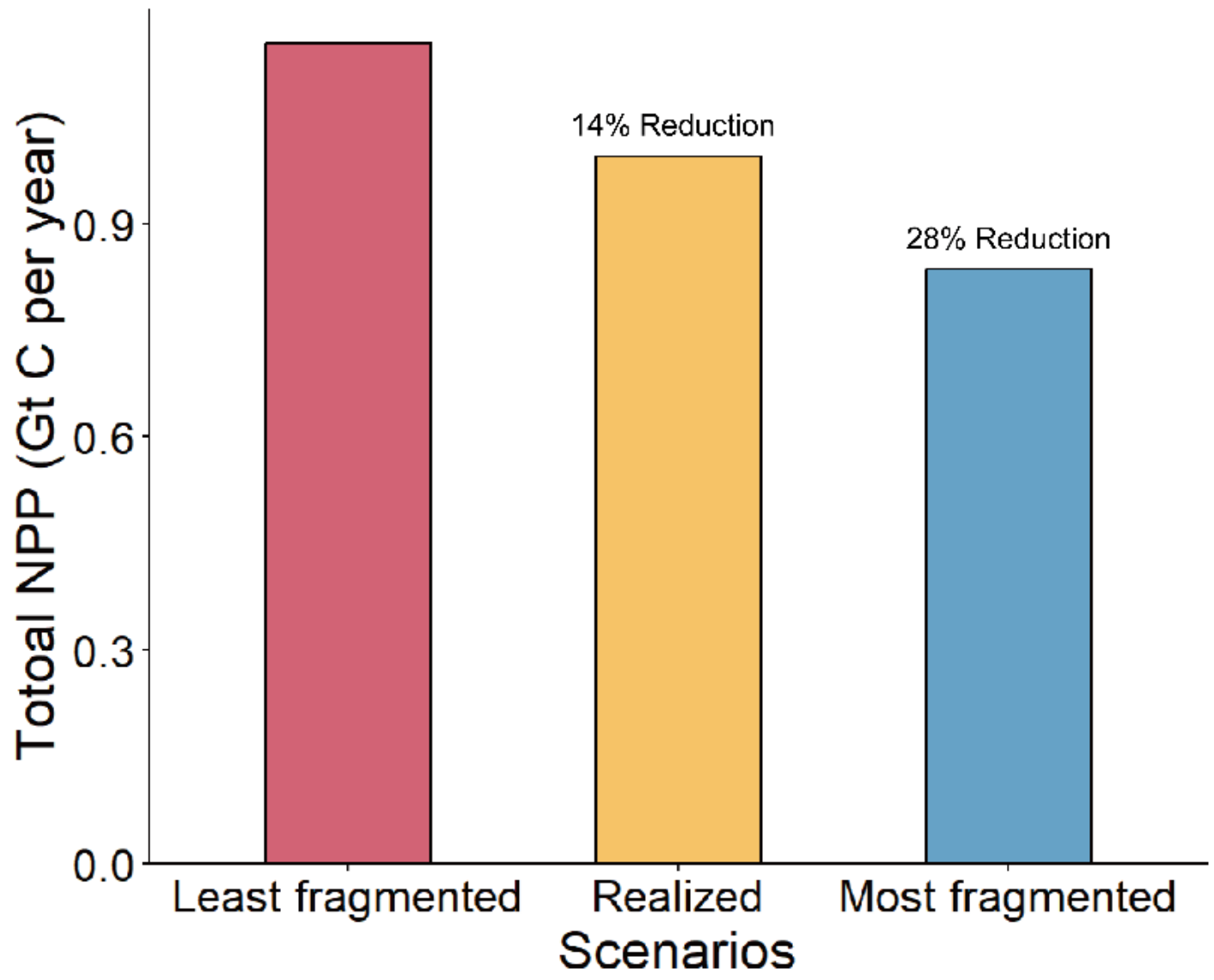

Fig. 3: Counterfactual effects of forest fragmentation on total annual NPP across CONUS. Bars show total forest net primary productivity (NPP; Gt C yr^{-1}) under three fragmentation scenarios while holding total forest area constant. Least fragmented represents an upper-bound configuration in which all forest area is assumed to function as large, contiguous patches and is assigned a per-area productivity of 0.80 kg C m^{-2} yr^{-1}, corresponding to the mean NPP of the largest patch size class ($\approx 10^{11}$ m^2; Table 1). Realized represents the observed landscape configuration, where total NPP is computed by summing patch-specific area multiplied by each patch's empirically estimated per-area NPP. Most fragmented represents a lower-bound configuration in which all forest area is assumed to function as small, highly fragmented patches and is assigned a per-area productivity of 0.58 kg C m^{-2} yr^{-1}, corresponding to the mean NPP of the smallest patch size class ($\approx 10^4$ m^2; Table 1). Percentage labels indicate reductions in total NPP relative to the least fragmented scenario, highlighting the potential carbon sequestration losses attributable to fragmentation alone.

[Comment 3]
The main policy paper cited as a justification for the study's policy statements is Lyons-White et al. (2020), where I struggle to find the direct connection to forest fragmentation issues. It could be made more explicit how Lyons-White's study applies to the study under consideration.
References: Lyons-White J, Pollard EH, Catalano AS, Knight AT (2020) Rethinking zero deforestation beyond 2020 to more equitably and effectively conserve tropical forests. One Earth 3:714–726. https://doi.org/10.1016/j.oneear.2020.11.007.
Response 15: We appreciate the reviewer's careful reading and agree that Lyons-White et al. (2020) does not address forest fragmentation explicitly. The purpose of citing this paper in

our policy discussion is not to argue that it directly analyzed fragmentation, but rather to highlight a broader point that is highly relevant to fragmentation outcomes: current “net zero deforestation” policy frameworks can permit forest loss and reconfiguration in ways that preserve total forest area while altering forest structure and connectivity.

Lyons-White et al. critically evaluates global “zero net deforestation” commitments and shows that, under many existing definitions, deforestation can be offset by reforestation or afforestation elsewhere: “Under zero net deforestation, land identified as forest may be cleared under some circumstances, provided any forest losses are compensated by forest regeneration, rehabilitation, or restoration.”. This net approach can unintentionally obscure landscape configuration changes, including fragmentation, even when gross forest loss is reduced. In this sense, their normative critique of policy frameworks supports our motivation to think beyond forest area alone and consider how forest configuration influences ecological function and carbon sequestration.

We have revised the manuscript in lines 57-73 to make this connection more explicit, clarifying that while Lyons-White et al. does not analyze fragmentation directly, its policy insights motivate the need to account for configuration effects like fragmentation in carbon and conservation policy. We have also added additional relevant policy literature that discusses limitations of net deforestation targets and the ecological importance of forest connectivity.

[Comment 4]
Finally, the authors find some negative relationships between forest patch size and productivity, for example, in the South American dry forest. How can those be explained?
Response 16: We thank the reviewer for highlighting this important point. While the dominant pattern across regions is a positive patch size–productivity relationship, we indeed observe weak or negative relationships in a small number of specific regions and biomes, including tropical dry forests in South America, temperate forests in Europe, and temperate broadleaf–mixed forests in Oceania. These exceptions suggest that patch size is not the primary constraint on productivity in all forest systems. In dry forests, productivity is often strongly limited by water availability and highly variable disturbance regimes (*23*) (e.g., fire, drought, grazing), which may override or even counteract fragmentation-related effects. Similarly, in some temperate regions, land-use history (*24*), forest management (*25*), or legacy effects of past disturbances may decouple present-day productivity from patch configuration. Because our analysis is designed to detect broad, large-scale scaling patterns rather than diagnose region-specific mechanisms, we do not attempt to attribute these negative relationships to a single causal process. We now explicitly acknowledge these cases in the Discussion in lines 202-208 and emphasize that they highlight important contexts in which fragmentation effects may be weak or reversed, underscoring the need for targeted regional studies to identify when and why patch size ceases to enhance forest productivity.

1. L. Ma, C. Shen, D. Lou, S. Fu, D. Guan, Ecosystem carbon storage in forest fragments of differing patch size. *Scientific Reports* **7**, 13173 (2017).
2. M. Islam, G. Deb, M. Rahman, Forest fragmentation reduced carbon storage in a moist tropical forest in Bangladesh: Implications for policy development. *Land Use Policy* **65**, (2017).
3. G. Yang, T. W. Crowther, T. Lauber, C. M. Zohner, G. R. Smith, A globally consistent negative effect of edge on aboveground forest biomass. *Nature Ecology & Evolution* **9**, 2036-2045 (2025).
4. N. M. Haddad *et al.*, Habitat fragmentation and its lasting impact on Earth's ecosystems. *Science Advances* **1**, e1500052.
5. R. Chaplin-Kramer *et al.*, Degradation in carbon stocks near tropical forest edges. *Nature Communications* **6**, 10158 (2015).
6. W. F. Laurance *et al.*, Biomass Collapse in Amazonian Forest Fragments. *Science* **278**, 1117-1118 (1997).
7. W. F. Laurance, P. Delamônica, S. G. Laurance, H. L. Vasconcelos, T. E. Lovejoy, Rainforest fragmentation kills big trees. *Nature* **404**, 836-836 (2000).
8. J. E. Reek *et al.*, Forest edges are globally warmer than interiors and exceed optimal temperatures for vegetation productivity. *Communications Earth & Environment* **6**, 635 (2025).
9. Y. Zou *et al.*, Fragmentation increased in over half of global forests from 2000 to 2020. *Science* **389**, 1151-1156 (2025).
10. G. B. Bonan, Forests and Climate Change: Forcings, Feedbacks, and the Climate Benefits of Forests. *Science* **320**, 1444 (2008).
11. D. Ellison *et al.*, Trees, forests and water: Cool insights for a hot world. *Global Environmental Change* **43**, 51-61 (2017).
12. P. M. Vitousek, R. L. Sanford, NUTRIENT CYCLING IN MOIST TROPICAL FOREST. *Annual Review of Ecology, Evolution, and Systematics* **17**, 137-167 (1986).
13. J. D. Aber, K. J. Nadelhoffer, P. Steudler, J. M. Melillo, Nitrogen Saturation in Northern Forest Ecosystems: Excess nitrogen from fossil fuel combustion may stress the biosphere. *Bioscience* **39**, 378-386 (1989).

14. A. McNeill, M. Unkovich, in *Nutrient Cycling in Terrestrial Ecosystems,* P. Marschner, Z. Rengel, Eds. (Springer Berlin Heidelberg, Berlin, Heidelberg, 2007), pp. 37-64.
15. H. Zhang, J. M. Chase, J. Liao, Habitat amount modulates biodiversity responses to fragmentation. *Nature Ecology & Evolution* **8**, 1437-1447 (2024).
16. R. H. Macarthur, E. O. Wilson, *The Theory of Island Biogeography*. (Princeton University Press, ed. REV - Revised, 1967).
17. T. Gonçalves-Souza *et al.*, Species turnover does not rescue biodiversity in fragmented landscapes. *Nature*, (2025).
18. J. Liang *et al.*, Positive biodiversity-productivity relationship predominant in global forests. *Science* **354**, aaf8957 (2016).
19. Y. Zhang, H. Y. H. Chen, P. B. Reich, Forest productivity increases with evenness, species richness and trait variation: a global meta-analysis. *Journal of Ecology* **100**, 742-749 (2012).
20. C. Bello *et al.*, Defaunation affects carbon storage in tropical forests. *Science Advances* **1**, e1501105.
21. F. Taubert *et al.*, Global patterns of tropical forest fragmentation. *Nature* **554**, 519-522 (2018).
22. D. M. Olson *et al.*, Terrestrial Ecoregions of the World: A New Map of Life on Earth: A new global map of terrestrial ecoregions provides an innovative tool for conserving biodiversity. *Bioscience* **51**, 933-938 (2001).
23. A. C. Staver, S. Archibald, S. Levin, The global extent and determinants of savanna and forest as alternative biome states. *Science* **334**, 230-232 (2011).
24. C. M. Kennedy, J. R. Oakleaf, D. M. Theobald, S. Baruch-Mordo, J. Kiesecker, Managing the middle: A shift in conservation priorities based on the global human modification gradient. *Glob Chang Biol* **25**, 811-826 (2019).
25. P. G. Curtis, C. M. Slay, N. L. Harris, A. Tyukavina, M. C. Hansen, Classifying drivers of global forest loss. *Science* **361**, 1108-1111 (2018).
